# Allopatric speciation of *Oroperipatus* velvet worms driven by Andean orogenesis and paleoclimate

**DOI:** 10.1016/j.isci.2026.118189

**Published:** 2026-11-10

**Authors:** José Pablo Barquero-González, V. Morley St. J. Read, Griselda Montiel-Parra, Gerardo Contreras-Félix, Arianna Tartara, Fernando Villagomez, Michael Heethoff, Georg Mayer, Ivo de Sena Oliveira

**Affiliations:** 1 Department of Zoology, Institute of Biology, University of Kassel, 34132 Kassel, Germany; 2 Centro Jambatu de Investigación y Conservación de Anfibios, Fundación Otonga, Geovanni Farina 566 y Baltra, San Rafael 170503, Quito, Ecuador; 3 Colección Nacional de Ácaros (CNAC), Departamento de Zoología, Instituto de Biología, Universidad Nacional Autónoma de México, 3er Circuito exterior s/n, Ciudad Universitaria, Coyoacán, Ciudad de México 04510, Mexico; 4 Colección Entomológica del Centro de Estudios en Zoología (CZUG), CUCBA, Universidad de Guadalajara, Camino Ramón Padilla Sánchez No. 2100, Zapopan 45200, Jalisco, México; 5 Animal Evolutionary Ecology, Technical University of Darmstadt, Schnittspahnstr. 2, 64287 Darmstadt, Germany

**Keywords:** andes biodiversity, island hopping, mountain range, neopatida, taxonomy

## Abstract

The role of Andean orogeny in the diversification of ancient, low-vagility terrestrial invertebrates remains poorly understood. Herein, we examined Andean onychophorans (*Oroperipatus*) to evaluate how climatic change and vicariant processes associated with Andean uplift influenced speciation and their disjunct distribution across the Americas. Morphological and molecular analyses of 13 species sampled across the taxon’s range revealed strong concordance between geography and phylogenetic structure, identifying two main lineages gathering eastern (Amazonian) and western (Pacific) Andean species, respectively. Divergence-time analyses suggest that these two lineages predate major Andean uplift and were likely shaped by paleoclimatic and vegetational changes. Later diversification was probably driven by climatic, ecological, and vicariant processes linked to the diachronous rise of the Andes. Close relationship among Ecuadorian, Galápagos, and Mexican species suggests stepwise dispersal via transient Pacific islands or independent Miocene-Pliocene overwater dispersal from Ecuador. Ten new species are described, increasing known *Oroperipatus* diversity by ∼50%.

## Highlights

• Phylogeny and biogeography suggest two lineages within *Oroperipatus*
• Later diversification was influenced by Andean uplift
• Ecuador-Galápagos-Mexico links suggest stepwise or overwater dispersal
• Ten new *Oroperipatus* species are described

## Graphical abstract

**Figure:**
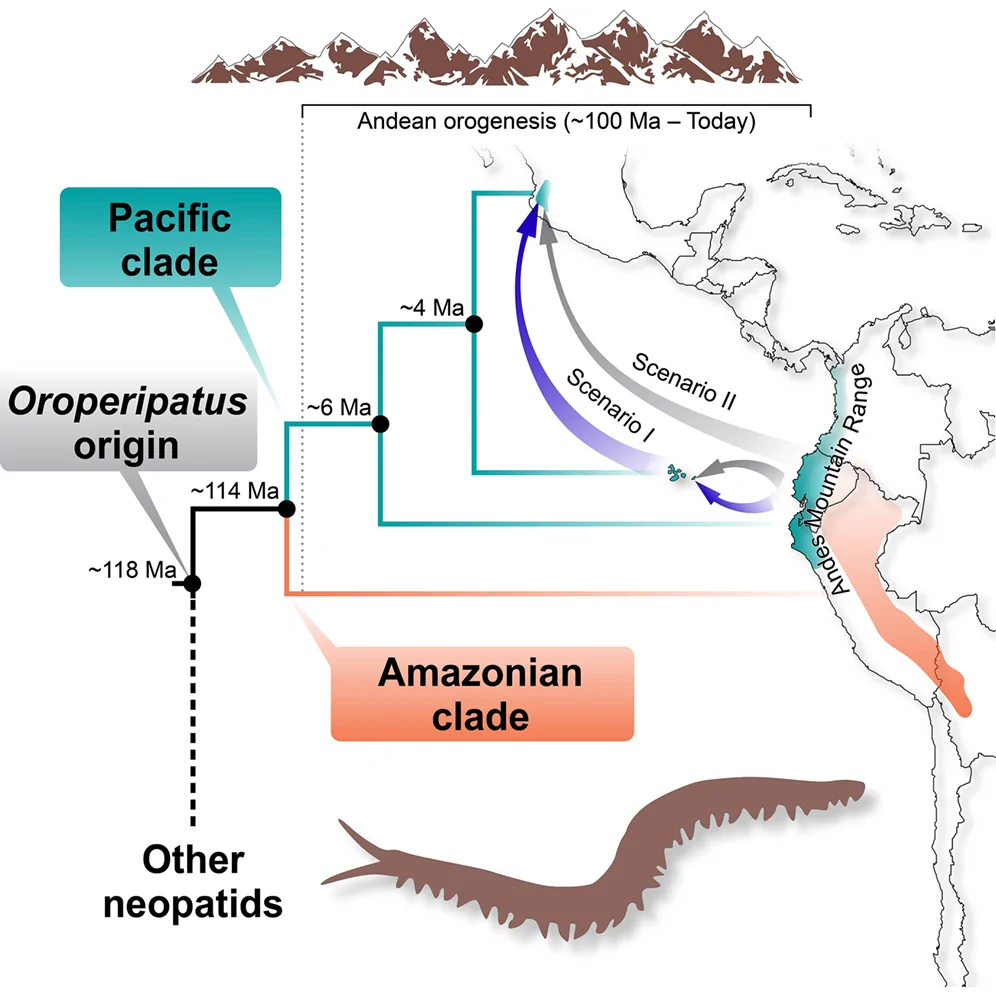

## Teaser

Biological sciences; Zoology; Evolutionary biology; Evolutionary ecology

## Introduction

Many taxa undergo radiation when heterogeneous, isolated geological systems rapidly emerge. These systems form “biological islands” with ecologically vacant habitats that provide temporal and spatial opportunities for adaptation, promoting cladogenesis through allopatric speciation and enhancing overall biodiversity.^1^^,^^2^^,^^3^ Mountains are particularly important in this process,^4^ harboring roughly one-third of all land species, making them critical biodiversity hotspots.^5^ The Andes exemplify this—a 7,000-kilometer South American mountain range hosting one of the world’s richest assemblages of endemic species.^6^ These mountains consist of three temporally distinct segments: the Southern Andes in Argentina and Chile (∼100 million years old [Ma]), the Northern Andes in Venezuela, Colombia, and Ecuador (∼80 Ma), and the Central Andes in Peru and Bolivia (∼70 Ma).^7^^,^^8^^,^^9^

Andean orogeny caused major regional transformations, including increased rainfall east of the mountain range, greater sediment flux into the Amazon region, and arid conditions to the west.^8^^,^^10^^,^^11^^,^^12^^,^^13^ These shifts promoted diversification across many lineages.^8^^,^^14^^,^^15^^,^^16^^,^^17^^,^^18^^,^^19^^,^^20^ However, limitations of previously studied taxa constrain our ability to assess the full impact of Andean uplift on diversification: some lineages are too young to reflect major tectonic influences, while others possess high dispersal ability that masks underlying biogeographic patterns.^21^ These limitations can be mitigated by examining taxa that retain strong phylogeographic signatures and are particularly suitable for biogeographic inference, such as Onychophora (velvet worms)—exclusively terrestrial, predatory invertebrates with limited mobility,^22^ narrow endemism,^23^ pronounced population genetic structure,^24^ small population sizes,^25^ and ancient evolutionary history.^26^

Extant onychophorans fall into two major subgroups: Peripatopsidae, distributed across temperate and tropical zones of the Southern Hemisphere, and Peripatidae, which inhabit pantropical regions including Tropical Africa, Southeast Asia, and the Neotropics.^26^^,^^27^ Based on early morphological observations, Bouvier^28^ subdivided the Neotropical peripatids (Neopatida *sensu* Oliveira et al.^29^) into a Caribbean group, distinguished by three distal foot papillae and nephridial tubercle between the third and fourth spinous pads, and an Andean group, defined by at least four distal foot papillae and nephridial tubercle within the third spinous pad. The latter group, mainly distributed along both flanks of the Andes with a few species in Mexico (Figures 1A and 1B),^28^ was subsequently formalized as *Oroperipatus*.^30^^,^^31^ Bouvier^28^ also suggested additional species groupings within *Oroperipatus* solely based on morphology. Currently, 21 nominal species are assigned to *Oroperipatus* (18 valid and three *nomina dubia*^32^^,^^33^) and the discovery of an undescribed species on the Galápagos islands expanded the geographical distribution of this taxon.^34^ Nevertheless, much of our understanding about *Oroperipatus* still derives from Bouvier’s^28^ early foundational study.

**Figure 1:**
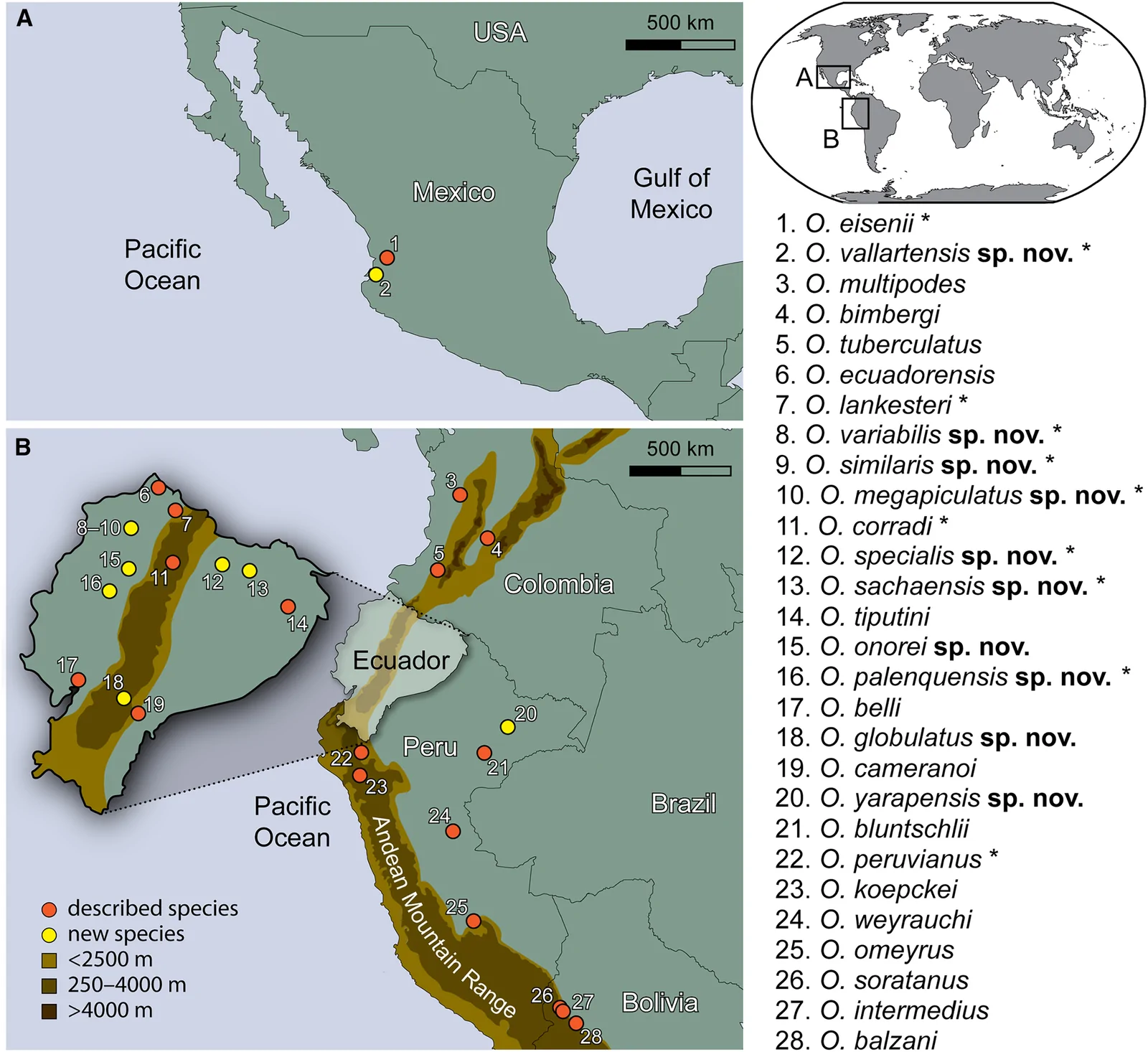
Geographic distribution of valid *Oroperipatus* species (A) Northernmost distribution of *Oroperipatus* in southern Mexico. (B) Distribution of *Oroperipatus* species in mainland South America. Note that Ecuador (inset) now harbors the highest number of described species. ∗Molecular data available. Overview maps. Circles indicate the type localities of previously (orange) and newly (yellow) described species.

Recent molecular studies corroborate Bouvier’s^28^ division of Neopatida into Caribbean and Andean groups but indicate that subclades within *Oroperipatus* do not match his morphology-based groupings.^23^^,^^26^^,^^29^^,^^35^^,^^36^^,^^37^^,^^38^^,^^39^^,^^40^^,^^41^ This suggests that diversification in *Oroperipatus* may have been driven by as-yet unidentified ecological, climatic and/or vicariant factors that did not produce corresponding morphological changes. The geographical distribution of *Oroperipatus* implies a potential role of Andean orogeny in cladogenesis, though the extent of this influence remains unclear.^40^

To address these questions, we studied museum and freshly collected material representing 13 *Oroperipatus* species, including their type species *O. lankesteri*, as well as *O. peruvianus*, *O. eisenii*, and ten new species described herein (Figures 1A and 1B). Using integrated morphological and molecular approaches, we characterized the phylogeographic structure, estimated divergence times, and assessed how Andean uplift shaped genetic and morphological diversification within this taxon. Our findings shed fresh light on the evolutionary history of *Oroperipatus* and the biogeographic processes underlying their diversity and disjunct distribution.

## Results

### Phylogenetic relationships and divergence-time estimates

Phylogenetic analyses included sequences from either 67 or 68 specimens (Table S2) and considered five datasets with final alignments ranging from 2,470 to 2,863 positions (Table S3). Translated amino acid sequences of the *COI* gene yielded no stop codons, confirming their protein-coding functionality. Maximum likelihood analyses produced consistent topologies supporting the monophyly of Peripatopsidae and Peripatidae, as well as Neopatida within the latter (Figures S1–S9). Neopatida comprises two well-supported subclades: one corresponding to *Oroperipatus* and another larger monophyletic assemblage including the remaining Neotropical peripatids (Figure 2). However, *Oroperipatus* is weakly recovered as paraphyletic when outgroup selection is expanded (Figures S5–S9); herein, we regard *Oroperipatus* as monophyletic. In our analyses, *Oroperipatus* was represented by 23 specimens assigned to 18 species, which were recovered in two distinct, well-supported lineages (Figures 2 and S1–S9). The first includes species from the eastern Andes, with samples from Peru and eastern Ecuador, and is here referred to as the Amazonian clade. The second comprises species distributed across the western Andes (Colombia and western Ecuador), Mexico, and the Galápagos, and is herein designated as the Pacific clade (Figures 2 and S1–S9).

**Figure 2:**
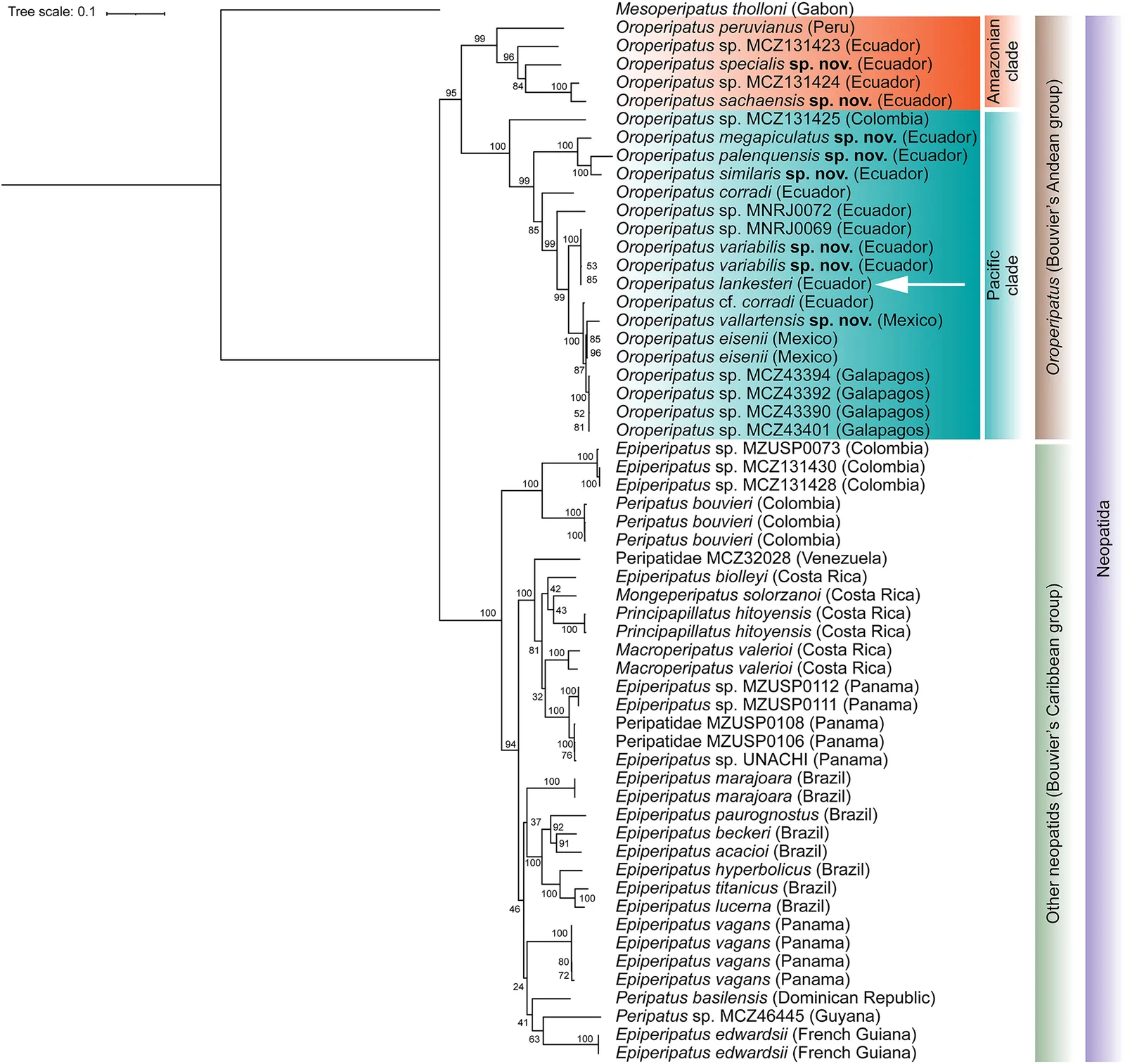
Phylogenetic relationships among studied *Oroperipatus* species Maximum likelihood topology combining nucleotide sequences of the *12S rRNA*, *16S rRNA*, *18S rRNA*, and *COI* genes. Numbers indicate bootstrap support values. Note the well-supported Amazonian (orange) and Pacific (blue) subclades of *Oroperipatus*, and the position of the type species *O. lankesteri* (arrow) within the latter. See also Figures S1–S9.

Molecular diagnostic analyses based on *18S rRNA* sequences identified six unique nucleotide positions characterizing *Oroperipatus*, whereas the Pacific and Amazonian clades were distinguished by seven and five exclusive nucleotide sites, respectively (Table 1). The type species, *Oroperipatus lankesteri*, was recovered within the Pacific clade (Figure 2). Across all phylogenetic reconstructions, each examined species was consistently resolved as a distinct evolutionary lineage (Figures 2 and S1–S9). Species delimitation analyses based on the single-locus markers *COI* and *16S rRNA* further corroborated the recognition of these taxa as distinct, non-conspecific lineages, in agreement with the phylogenetic analyses, whereas results derived from the *12S rRNA* dataset were incongruent with both the species delimitation outcomes from the other markers and the phylogenetic evidence (see Document S1 for details).

**Table 1:** Diagnostic molecular characters in the *18S rRNA* gene distinguishing *Oroperipatus* and its two major subclades

Diagnostic positions	Outgroup		*Oroperipatus*		
	Peripatopsidae	Other peripatids	Genus	Amazonian clade	Pacific clade
1045	–	A	G/A	**G**	A
1048	–	A	C/A	**C**	A
1082	–	G	G/A	G	**A**
1091	–	C	C/T	C	**T**
1175	–	C	**T**	T	T
1177	–	A/T	T/C	T	**C**
1231	–	C	**T**	T	T
1265	–	C/T	C/T/A	C/T	**A**
1266	–	G	G/A	G	**A**
1273	–	T	**C**	C	C
1289	–	C	**T**	T	T
1303	–	T	T/C	T	**C**
1304	–	G	G/C	G	**C**
1925	C/G/T	G	**A**	A	A
1946	C/G	A/G	T/A	**T**	A
2169	C/G/T	G	A/G	**A**	G
2268	T	G/T	**A**	A	A
2328	G	A/T	C/T	**C**	T

Position numbering follows the final *18S rRNA* gene alignment. Each diagnostic character is highlighted in bold; n-dashes indicate gaps in the alignment.

Divergence-time estimates within Peripatidae (Figures 3A and S10) suggest an initial separation between the Southeast Asian (*Eoperipatus*) and West Gondwanan peripatids (Neopatida and *Mesoperipatus*) during the Permian (Capitanian stage, ∼261 Ma; 195–314 Ma, 95% highest posterior density [HPD] interval). The subsequent divergence between tropical African *Mesoperipatus* and Neopatida occurred during the Pliensbachian stage (∼187 Ma; 123–250 Ma, 95% HPD interval), in the Early Jurassic. Within Neopatida, the Caribbean and Andean lineages diverged in the Early Cretaceous (Aptian stage, ∼118 Ma; 83–153 Ma, 95% HPD interval), while the split between the Amazonian and Pacific clades of *Oroperipatus* occurred 4 million years later (Early Cretaceous, Aptian stage, ∼114 Ma; 80–151 Ma, 95% HPD interval).

**Figure 3:**
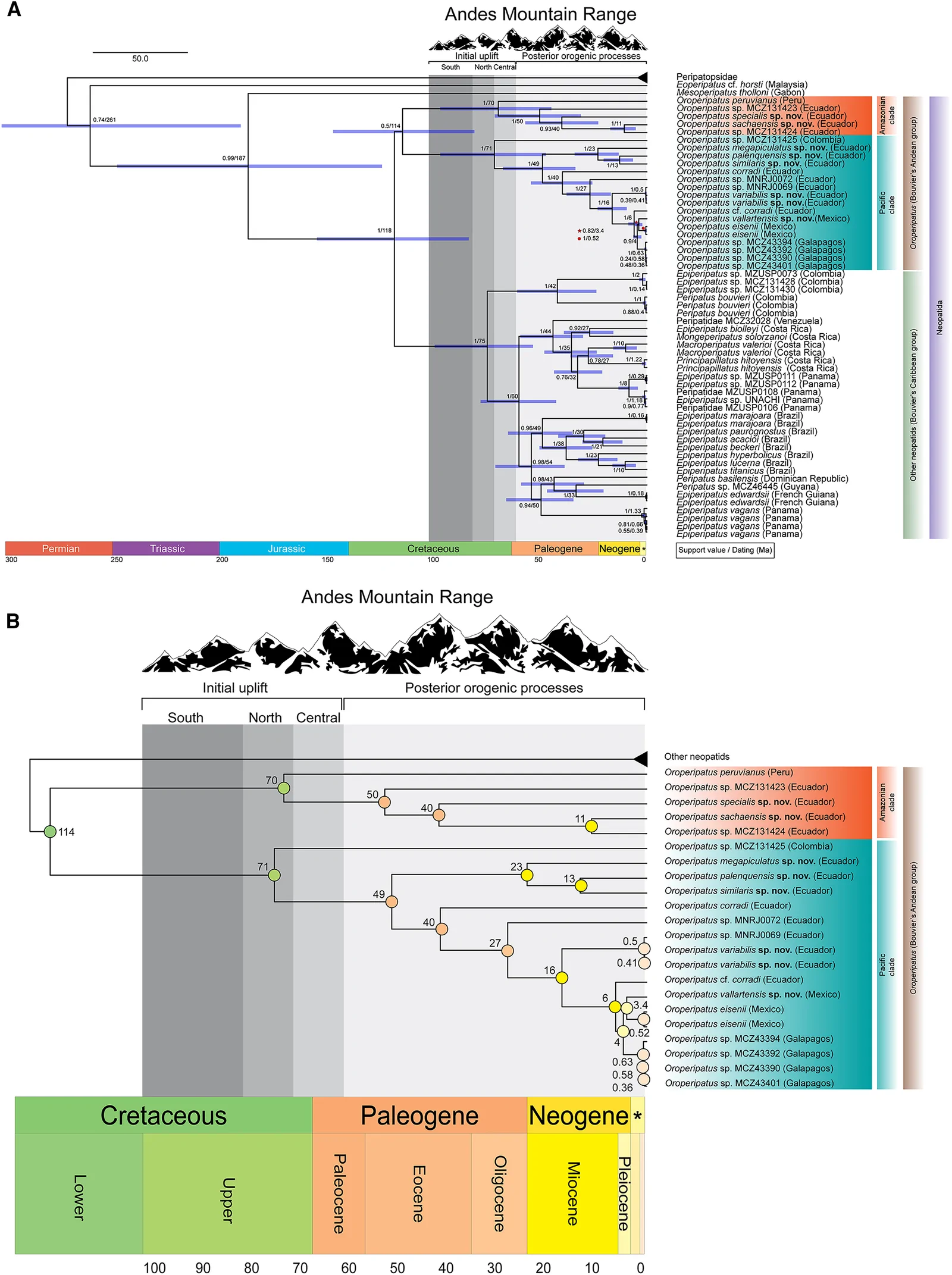
Chronogram of the Peripatidae dataset inferred in BEAST (A) Overview of estimate divergence times within Peripatidae. (B) Detail of estimate divergence times within the Amazonian (orange) and Pacific (blue) subclades of *Oroperipatus*. Circles mark cladogenetic events, color-coded by geological period. Values indicate the mean node age. See also Figure S10. Geological scale shown below, with an asterisk marking the Quaternary period; timing of Andean orogeny *sensu* Pérez-Escobar^17^ is indicated above. Horizontal bars represent the 95% highest probability density (HPD) intervals for divergence times.

Cladogenetic events within both the Amazonian and Pacific clades of *Oroperipatus* span from the Late Cretaceous through the Pleistocene (Figure 3B). In the Amazonian clade, the earliest divergence occurred between the Peruvian (*O. peruvianus*) and eastern Ecuadorian lineages during the Late Cretaceous (Maastrichtian; ∼70 Ma; 44–96 Ma, 95% HPD interval). Diversification of eastern Ecuadorian species was subsequently shaped by three additional cladogenetic events during the Eocene (Ypresian; ∼50 Ma; 31–71 Ma, 95% HPD interval and Bartonian; ∼40 Ma; 23–57 Ma, 95% HPD interval), and the Miocene (Tortonian; ∼11 Ma; 5–17 Ma, 95% HPD interval), respectively. Within the Pacific clade, the earliest major split occurred during the Late Cretaceous (Maastrichtian; ∼71 Ma; 47–96 Ma, 95% HPD interval), separating a Colombian lineage from a larger clade comprising western Ecuadorian, Galápagos, and Mexican taxa. In this larger clade, numerous divergence events span from the Eocene (Ypresian; ∼49 Ma; 33–67 Ma, 95% HPD interval) to the Pleistocene (Chibanian; ∼0.36 Ma; 0.0117–0.9 Ma, 95% HPD interval), with Mexican and Galápagos taxa diverging from western Ecuadorian species during the Miocene (Messinian; ∼6 Ma; 3–9 Ma, 95% HPD interval) and subsequently from each other during the Pliocene (Zanclean, ∼4 Ma; 3–7 Ma, 95% HPD interval).

### Taxonomy

The material analyzed comprises 13 distinct species, including *O. eisenii*, *O. peruvianus*, *O. lankesteri*, and 10 species new to science. All specimens analyzed unambiguously exhibited the principal diagnostic character of *Oroperipatus*: two or more anterior and two or more posterior distal foot papillae, totaling 4–7 distal foot papillae. In this taxonomic account, we reassess the diagnostic features of nominal taxa (excluding *O. lankesteri*) and formally describe the new species following the provisions of the International Code of Zoological Nomenclature (ICZN). To maintain conciseness, expanded etymology, description of non-diagnostic features, and the differential diagnosis of each species are provided separately (Document S1, Figures S11–S35). Nominal and newly described species are presented in alphabetical order, respectively.

ONYCHOPHORA Grube, 1850^42^

urn:lsid:zoobank.org:act:42A5A404-012F-4CCA-803B-1B216DB47A1B.

PERIPATIDAE Audouin and Milne-Edwards, 1832^43^

urn:lsid:zoobank.org:act:7EBCC744-DC25-4E15-9B83-2EEC530DC71F.

*Oroperipatus* Cockerell, 1908^30^

urn:lsid:zoobank.org:act:022A6A07-44D4-4573-B13D-91401A20277.

Type species: *Oroperipatus lankesteri* (Bouvier, 1899^44^)

Holotype: Deposited in The Natural History Museum, London, UK.

Genus diagnosis: Each foot with 4–7 distal foot papillae, ≥ 2 anterior and ≥ 2 posterior (Figures 4 and S12A–S12L).

**Figure 4:**
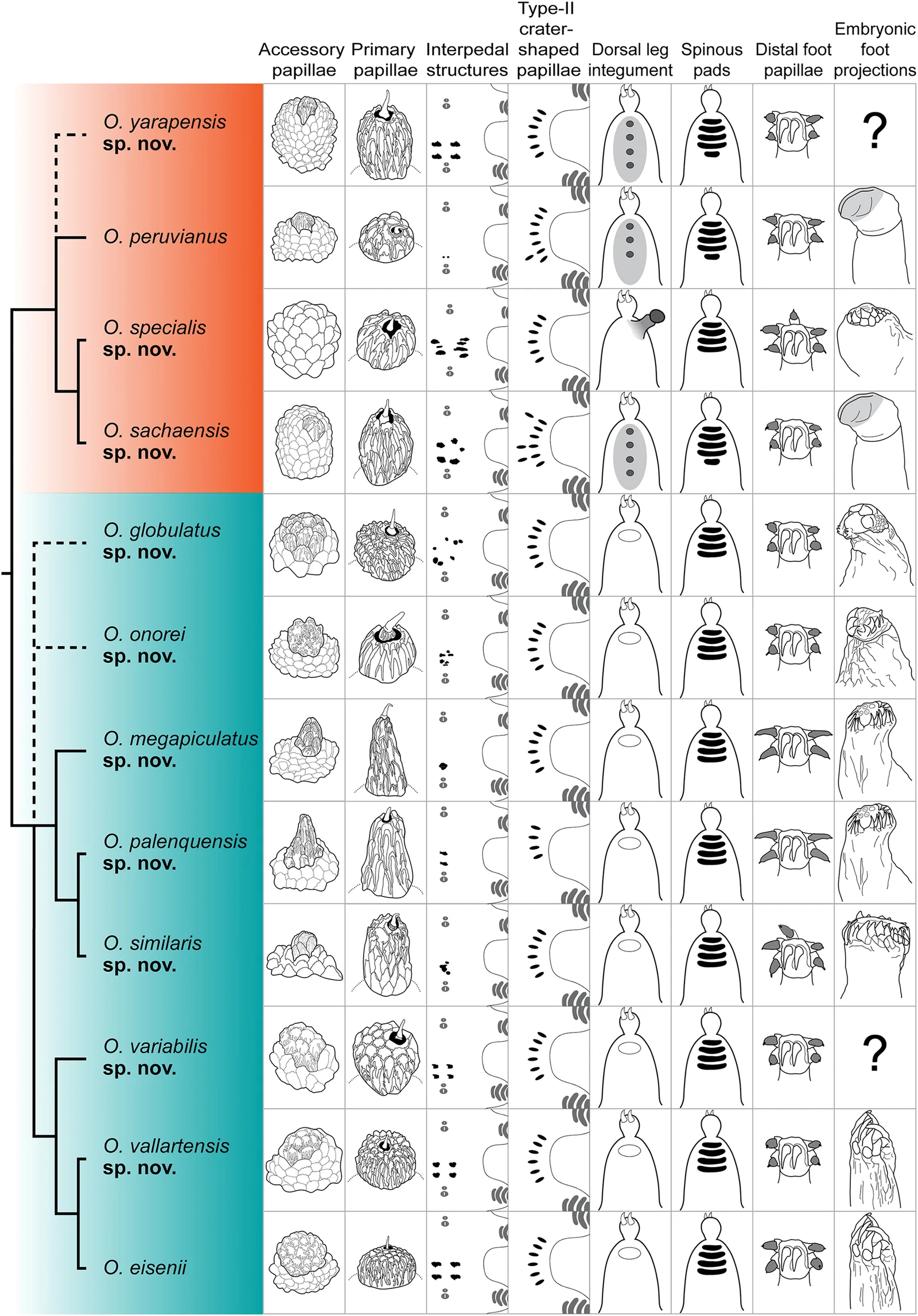
Phylogenetic relationship and morphological diagnostic features of studied *Oroperipatus* species Dashed lines in phylogenetic tree (left) indicate the inferred position of species not represented in the molecular dataset, see Document S1 for inference criteria. The Amazonian subclade is indicated in orange; the Pacific subclade in blue. Simplified drawings of diagnostic features based on SEM images. See also Document S1.

*Oroperipatus eisenii* (Wheeler, 1898^45^)

urn:lsid:zoobank.org:act:2EB763EF-6639-4830-948F-2FC09116A282.

Synonyms: None.

Holotype: Not designated. Syntype deposited in the Museum of Comparative Zoology, Harvard University, Cambridge, USA.

Type locality: Mexico, Nayarit, outskirts of Tepic, ∼1,220 m above sea level (ASL).

Material examined: ZMH-ARA-A00320101, one ♀ in 70% ethanol (partially prepared for SEM); MEXICO, Guadalajara, Colonia del Sur, Nicaragua 2036, 20°39′2.51″N, 103°22′30.98″W, 1,586 m ASL, 17.X.2007, M.E. González Hernández Col. (collector).

Diagnosis: Dorsal primary papillae with two apical piece types; large type similar to basal piece in size, asymmetrical, elliptical, wider than tall, with six scale ranks anteriorly and three posteriorly; small type symmetrical, smaller than basal piece, with 3–4 scale ranks (Figures 4, S13A, and S14A); apical-most scales on apical piece irregular, with wide, smooth end; other scales keel-like (Figures 4, S13A, and S14A). Accessory papillae similar in shape to primary ones, with apical-most scales forming spherical, smooth tip (Figures 4 and S15A). Interpedal structures round, with 4–7 large, finely granulated scales (Figures 4 and S16A); two pairs of interpedal structures per segment. Embryonic foot projections large, claw-like, smooth, taller than wide, with polygonal to round basal bulks (Figures 4 and S17A).

*Oroperipatus peruvianus* (Brues, 1917)^46^

urn:lsid:zoobank.org:act:D9AA25E7-C505-4093-B115-9EC411D27FF8.

Synonyms: None.

Holotype: Deposited in the Museum of Comparative Zoology, Harvard University, Cambridge, USA.

Type locality: Peru, Cajamarca, Tabaconas, near Huancabamba, ∼1,830 m ASL.

Material examined: ZMH-ARA-A0032094-97, three ♀♀ and one ♂ in 100% ethanol (one ♀ and one ♂ partially prepared for SEM); PERU, Cajamarca, San Ignacio, Santuario Nacional Tabaconas Namballe, 5°9′25.28″S, 79°18′45.92″W, 2,848 m ASL, 2009, Maik Dobiey Col.

Diagnosis: Dorsal primary and accessory papillae with flat, smooth, blunt-ended scales (Figures 4, S13B, S14B and S15B). Apical piece of dorsal primary papillae spherical, asymmetrical, as wide as tall, with four scale ranks anteriorly and two posteriorly (Figures 4, S13B and S14B); apical-most scales around sensory bristle serrated, with sharp edge. Accessory papillae similar in shape to primary, with four modified, flattened apical-most scales (Figures 4 and S15B). Interpedal structures small, round, with 5–10 polygonal, finely structured scales in the middle and 7–9 regular scales around (Figures 4 and S16B). Six to eight type-II crater-shaped papillae in ventral and ventro-lateral rows (Figures 4 and S18A). Dorsal leg integument lacking dermal papillae (Figures 4, S18B and S19B), with smooth to finely textured flat scales (Figure S18B) and a longitudinal row of 3–4 round bumps (Figures 4, S18B and S18C); bean-shaped papillae absent (Figures 4, S18B, S18C and S19B). Embryonic foot projections absent (Figures 4, 5A and S17B).

**Figure 5:**
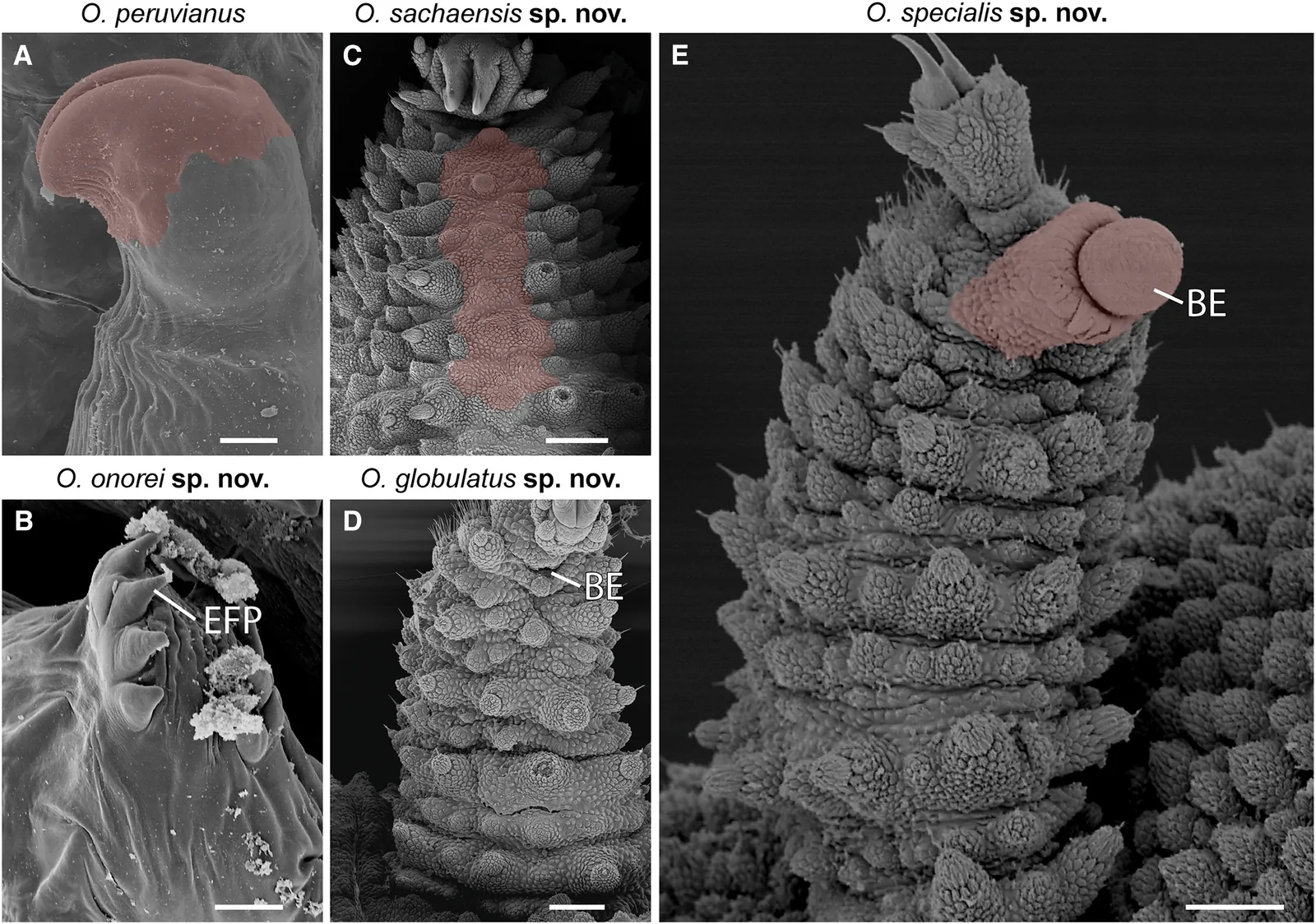
Scanning electron micrographs of selected characteristic features identified herein (A and B) Embryonic foot projections may be absent in some representatives of the Amazonian subclade, as exemplified for *O. peruvianus* (red in A), but present in other species, such as *O. onorei* sp. nov. (B). Scale bars, 40 μm and 15 μm, respectively. (C–E) Bean-shaped papilla may be either absent, as in *O. sachaensis* sp. nov.(C), or reduced in size, as in *O. globulatus* sp. nov., or modified into an eversible structure, as in *O. specialis* sp. nov. (red in E). Scale bars, 200 μm and 100 μm, respectively. Note the reduction of dermal papillae on the dorsal leg surface of *O. sachaensis* sp. nov. (red in C). Scale bars, 100 μm. BE, bean-shaped papilla; EFP, embryonic foot projection. See also Figures S17–S19, and S21. Distal is up in all images.

*Oroperipatus globulatus* sp. nov.

urn:lsid:zoobank.org:act:3CF51A2C-4B24-4D38-B992-90D65B721BE1.

Holotype: QCAZI-282080, one ♀ in 70% ethanol; Ecuador, Azuay, Mazan River Valley, 1986, V.M. ST.J. Read Col.

Paratype: ZMH-ARA-A00320100, one ♀ in 70% ethanol (partially prepared for SEM); as for holotype.

Etymology: The species name derives from the Latin word *globulus* (diminutive for “ball”) and the suffix *atus* (“having the nature of”, “state or condition of”), in reference to the characteristic globular shape of apical pieces in dorsal primary papillae (Document S1).

Diagnosis: Apical piece of dorsal primary papillae well-developed, larger than basal piece, globular, asymmetrical, with 5–7 anterior and 2–3 posterior scale ranks (Figures 4, S13C and S14C). Scales on apical piece keel-like, with smooth outer surface (Figures 4, S13C and S14C). Basal piece of dorsal primary papillae asymmetrical, small, with 1–3 scale ranks (Figure S14C). Accessory papillae similar to primary papillae in shape, with blunt-ended apical-most scales forming a globular tip (Figures 4 and S15C). Interpedal structures variable in number, shape, and arrangement, small, with 1–2 flat granulated scales (Figures 4 and S16C); 6–7 interpedal structures per segment (Figure S16M). Bean-shaped papillae reduced in size, inconspicuous (Figure 5D). Embryonic foot projections few in number (∼four pairs), short, smooth, as high as wide, restricted to small distal foot portion (Figures 4 and S17C).

*Oroperipatus megapiculatus* sp. nov.

urn:lsid:zoobank.org:act:63641C06-B477-49EA-8AC9-929CC6740CD0.

Holotype: QCAZI-282081, one ♀ in 70% ethanol (partially prepared for SEM); Ecuador, Esmeraldas, La Yuca, private reserve Canandé, 0°29′51.50″N, 79°13′28.25″W, 350 m ASL, 17.V.2022, A. Tartara and F. Villagomez Col.

Etymology: The species name comes from the Greek word *mega* (“large”) combined with the Latin word *apiculus* (diminutive for “apex”, “apical”) and the suffix *atus* (having the nature of, state or condition of), in reference to the prominent apical piece in dorsal primary papillae (Document S1).

Diagnosis: Apical pieces of dorsal primary papillae symmetrical, rod-like to conical, taller than wide, with 4–5 ranks of pointy scales (Figures 4, S13D and S14D); apical piece particularly long in dorsal leg region (Figure S19D). Accessory papillae similar to primary papillae in shape, with apical-most scales forming conical, taller than wide, tip (Figures 4 and S15D). Distal foot papillae semi-ovoid, with large, taller than wide, rod-like to cylindrical apical pieces, and short blunt-ended sensory bristle (Figures 4 and S12D). Interpedal structures round, with smooth textured scales, surrounded by four polygonal structured scales (Figure S16D); one pair of interpedal structures per segment. Embryonic foot projections thorn-shaped, blunt-ended, smooth, taller than wide, forming ring around claws (Figures 4 and S17D).

*Oroperipatus onorei* sp. nov.

urn:lsid:zoobank.org:act:76A520F8-6AE9-4A45-8FD9-70B8A1E56903.

Holotype: QCAZI-282082, one ♀ in 100% ethanol; Ecuador, Santo Domingo de los Tsáchilas, environs of San José de Alluriquín, near the Otonga and the Otongachi Reserves, 0°19′S, 78°58′W, 920 m ASL, V. 1985, G. Onore Col.

Paratype: ZMH-ARA-A0032093, one ♀ in 100% ethanol (partially prepared for SEM); as for holotype.

Etymology: This species is named after Giovanni Onore, an Italian entomologist based in Ecuador and founder of the Quito Catholic Zoology Museum (QCZM), who collected the examined specimens (Document S1).

Diagnosis: Apical piece of dorsal primary papillae short, symmetrical, semi-elliptical, as wide as tall, with two scale ranks (Figures 4, S13E and S14E); apical piece scales keel-like on basal-most rank, paddle-like on apical-most rank (Figures S13E and S14E). Large accessory papillae round, similar in size to primary ones (Figures S15E and S30E). Interpedal structures finely granulated, with 1–6 flat, granulated scales (Figure S16E); six interpedal structures per segment, anterior- and posterior-most interpedal structures unpaired, bar-like or roundish, separated by two pairs of irregular or bar-like ones (Figures 4 and S16N). Embryonic foot projections few in number, smooth, pointy, taller than wide (Figures 4, 5B and S17E).

*Oroperipatus palenquensis* sp. nov.

urn:lsid:zoobank.org:act:A72AC677-F8D6-4415-80A1-95CC789BD517.

Holotype: QCAZI-282083, one ♀ in 70% ethanol; Ecuador, Los Rios, Centro Científico Río Palenque, 175 m ASL, 10.VII.1977, K. Miyata Col.

Paratypes: ZMH-ARA-A0032090-92, three ♀♀ in 70% ethanol (one ♀ partially prepared for SEM); as for holotype, 10.VII.1980, R. Castello Col.

Etymology: The species is named after its type locality along the banks of the river Palenque (Document S1).

Diagnosis: Apical pieces of dorsal primary papillae symmetrical, taller than wide, conical to cylindrical, with three (rarely 2–4) ranks of keel-to paddle-like scales (Figures 4, S13F and S14F). Dorsal accessory papillae with apical-most scales forming a conical, taller than wide, tip (Figures 4 and S15F). Type-II crater-shaped papillae irregular in number, shape and arrangement; 4–6 papillae in ventral and ventro-lateral rows (Figures 4 and S18D). Interpedal structures variable, with four flat granulated scales (Figure S16F). Distal foot papillae with large, cylindrical apical pieces, taller than wide, and thorn-shaped sensory bristle (Figures 4 and S12F). Embryonic foot projections numerous, pointy, smooth, higher than wide, forming a ring around claws; apical-most foot region lacks projections, with variable round swellings (Figures 4 and S17F).

*Oroperipatus sachaensis* sp. nov.

urn:lsid:zoobank.org:act:1A0569E4-3032-4ED1-9A62-596F3D48CF3A.

Holotype: QCAZI-282084, one ♀ in 70% ethanol (partially prepared for SEM); Ecuador, Orellana, La Joya de los Sachas, 02.VI.1983, J. Boos Col.

Etymology: The species is named after its type locality, La Joya de los Sachas (short Sacha), which means “forest” in the Quechua language (Document S1).

Remarks: Species previously identified as *Oroperipatus quitensis* (Read 1988^47^: 210)

Diagnosis: Apical pieces of dorsal primary papillae asymmetrical or symmetrical, semi-ovoid to ovoid, as tall as wide, with 2–6 scale ranks (Figures 4, S13G and S14G); apical-most scales on apical piece paddle-like, as wide as high, remaining scales keel-like (Figure S13G). Dorsal accessory papillae with apical-most scales paddle-like, forming a conical, taller than wide, tip (Figures 4 and S15G). Type-II crater-shaped papillae variable in number; 6–9 papillae in ventral and ventro-lateral rows (Figures 4 and S18G). Two to five interpedal structures per segment (Figures 4 and S16O), each oval in shape, with 8–34 flat, finely granulated scales (Figures 4 and S16G). Dorsal leg integument lacking dermal papillae (Figures 4, 5C, S18E and S19G), covered with scales, with a longitudinal row of 3–4 globular bumps (Figures 4, S18E and S18F). Bean-shaped papillae absent (Figures 4, S18E and S19G). Embryonic foot projections absent (Figures 4 and S17G).

*Oroperipatus similaris* sp. nov.

urn:lsid:zoobank.org:act:2E6BE972-77ED-49D9-8479-0DD713946533.

Holotype: QCAZI-282085, one ♀ in 70% ethanol (partially prepared for SEM); Ecuador, Esmeraldas, La Yuca, private reserve Canandé, 0°30′42.45″N, 79°12′12.93″W, 377 m ASL, 17.III.2022, A. Tartara and F. Villagomez Col.

Etymology: The Latin term *similaris* translates to “like” or “resembling”, referring to the morphological similarity of this species to *Oroperipatus megapiculatus* sp. nov. (Document S1).

Diagnosis: Apical pieces of dorsal primary papillae symmetrical, cylindrical to rod-like, taller than wide, with 3–6 scale ranks (Figures 4, S13H and S14H); apical-most scales on apical piece paddle-like, remaining scales pointy (Figures 4, S13H and S14H). Apical pieces particularly long on dorsal leg (Figure S19H) and anal region. Accessory papillae similar to primary papillae in shape, with apical-most scales forming semi-elliptical, taller than wide, tip (Figures 4 and S15H). Four to five distal foot papillae: two anterior, two posterior and none or one dorsal; five papillae in midbody legs (Figures 4 and S12H). Distal foot papillae cylindrical, with large, taller than wide, conical to rod-like apical pieces, and short blunt-ended sensory bristle (Figures 4, S12H and S19H). Interpedal structures variable in shape, flat, with 1–12 granulated, irregular smooth scales, fused or separated (Figures 4 and S16H); 1–2 pairs of interpedal structures per segment. Embryonic foot projections numerous, long, thorn-shaped, smooth, taller than wide (Figures 4 and S17H).

*Oroperipatus specialis* sp. nov.

urn:lsid:zoobank.org:act:B99FC819-7E68-4259-AAB9-4CB2B02288F3.

Holotype: QCAZI-282086, one ♂ in 70% ethanol (partially prepared for SEM); Ecuador, Napo, San Rafael, environs of San Rafael Falls, 1,500 m ASL, I.1985, L. Onore Col.

Paratypes: ZMH-ARA-A0032088-89, two ♀♀ in 70% ethanol (one ♀ partially prepared for SEM); as for holotype.

Etymology: The species epithet is derived from the Latin term *specialis*, meaning “special” or “particular”, in reference to the modified bean-shaped papilla that, to date, is unique to this species (Document S1).

Diagnosis: Apical piece of dorsal primary papillae symmetrical, ovoid to globular, with 1–3 scale ranks (Figures 4, S13I and S14I); apical-most rank with paddle-like scales, as wide as high, remaining scales keel-like (Figures S13I and S14I). Four to five distal foot papillae, two anterior, two posterior, and one dorsal; five papillae in midbody legs (Figures 4 and S12I). Interpedal structures irregular in arrangement, each with 1–10 large, flat, granulated scales (Figure S16I); seven interpedal structures per segment (Figures 4 and S16P). Bean-shaped papilla modified, eversible, stalked, located proximally to foot on dorsal region of all legs (Figure 4); retracted: enclosed within integumentary pouch (Figure S21A); everted: prominent, as high as foot length, extending anteriorly, with conical stalk separated from spherical apex by a constriction (Figures 4, 5E, S19I, S21B and S21C); apex with or without sensory bristle (Figure S21D). Embryonic foot projections few in number and small, smooth, thorn-shaped, higher than wide, with polygonal basal bulks (Figures 4 and S17I).

*Oroperipatus vallartensis* sp. nov.

urn:lsid:zoobank.org:act:036D18FB-3CBF-481B-B98E-7173B8414AEB.

Holotype: CNAC/Ony00086, one ♀ in 70% ethanol; Mexico, Jalisco, Puerto Vallarta, 20°35′47.93″N, 105°14′4.68″W, 300 m ASL, 13.V.2013, R. García de Quevedo Col.

Paratypes: ZMH-ARA-A0032098-99, one ♀ in 70% ethanol (partially prepared for SEM); as for holotype, 18.V.2013, F. Cupul-Magaña Col; one ♀ in 70% ethanol; as for holotype, 26.V.2013, P. Medina Col.

Etymology: The species is named after its type locality at Puerto Vallarta, Jalisco State, Mexico (Document S1).

Remarks: Species previously identified as *Oroperipatus eisenii* (Cupul-Magaña and Navarrete-Heredia^48^: 546).

Diagnosis: Apical piece of dorsal primary papillae well-developed, globular, as large as basal piece, symmetrical (Figures 4 and S14J) or asymmetrical (Figures S13J and S14J); symmetrical apical pieces with 3–4 scale ranks (Figures 4 and S14J), asymmetrical with 4–5 scale ranks anteriorly and 2–4 scale ranks posteriorly (Figures S13J and S14J). Apical-most scales on apical piece irregular, serrated or with wide, smooth end; remaining scales keel-like (Figures S13J and S14J). Accessory papillae similar to primary papillae in shape, with apical-most scales forming globular, smooth tip (Figures 4 and S15J). Interpedal structures oval (Figure 4), with 1–13 unevenly distributed scales; scales with short villus-like to roundish, smooth projections (Figure S16J). Embryonic foot projections claw-like, smooth, higher than wide, with polygonal to round basal bulks (Figures 4 and S17J).

*Oroperipatus variabilis* sp. nov.

urn:lsid:zoobank.org:act:0E78646B-2EC9-42BA-81DA-660778F2E1E7.

Holotype: QCAZI-282087, one ♀ in 100% ethanol; Ecuador, Esmeraldas, La Yuca, private reserve Canandé, 0°30′39.80″N, 79°12′43.59″W, 372 m ASL, 28.IV.–10.VI.2022, A. Tartara and F. Villagomez Col.

Paratypes: ZMH-ARA-A00320102-103, two ♀♀ in 100% ethanol (one ♀ partially prepared for SEM); as for holotype.

Etymology: The Latin word *variabilis* translates to “changeable” or “variable”, referring to the morphological variability in the apical pieces of dorsal primary papillae (Document S1).

Diagnosis: Dorsal primary papillae variable, of four distinct size and morphology classes: (I) largest papillae with globular, asymmetrical apical piece, larger than basal piece, with 2–3 scale ranks anteriorly and 4–5 posteriorly (Figure S21E); (II) large papillae with semi-ovoid, asymmetrical apical piece, as large as basal piece, with 2–3 scale ranks anteriorly and 4–5 posteriorly (Figures 4, S13K and S21F); (III) mid-sized papillae with symmetrical, round apical piece, as large as basal piece, with 2–3 scale ranks (Figures S14K and S21G); (IV) small papillae with inconspicuous, asymmetrical apical piece nearly indistinguishable from basal piece, with 1–3 scale ranks (Figure S21H). Class I and II primary papillae on alternating plicae; class III and IV primary papillae on all plicae (Figure S30K).

*Oroperipatus yarapensis* sp. nov.

urn:lsid:zoobank.org:act:65B2BE0C-7F94-4B71-B53D-F50E0229F182.

Holotype: MZUCR76, one ♂ in 70% ethanol (partially prepared for SEM); Peru, Loreto, downstream of Yarapa River, 117 m ASL, 15.XI.1993, W. Lamar Col.

Etymology: The species is named after its type locality along the banks of Yarapa River, in the Department of Loreto, Peru (Document S1).

Diagnosis: Apical piece of dorsal primary papillae semi-ovoid, small, symmetrical, about 1/3–2/3 of basal piece size, with 3–4 scale ranks (Figures 4, S13L and S14L). Accessory papillae similar to primary papillae in shape, with apical-most scales blunt-ended, smooth, forming a semi-ovoid tip (Figures 4 and S15L). Male crural tubercle subdivided into two regions: base with flat scales and few to several anterior sensory bristles; tip ∼ half base size, with irregular keel-like scales and slit or T-shaped opening surrounded by smooth surface (Figure S20L). Dorsal leg integument lacking dermal papillae (Figures 4, S18H and S19L), with finely textured scales and longitudinal row of 3–4 round bumps (Figures 4, S18H and S18I); bean-shaped papillae absent (Figures 4 and S19L).

### Species delimitation

Single-locus species delimitation analyses based on the *COI*, *12S rRNA*, and *16S rRNA* markers support the hypothesis that the proposed new species are not conspecific (Figure S37). Nevertheless, several taxa, particularly unidentified specimens retrieved from online databases, exhibited inconsistent delimitation patterns depending on the molecular marker analyzed (Figure S37). For example, Mexican specimens assigned to *O. eisenii* and *O. vallartensis* sp. nov. were recovered as conspecific with additional unidentified taxa (MCZ43390, MCZ43392, MCZ43394, and MCZ43401) in *COI*-based analyses. Comparable results were obtained using the *16S rRNA* marker, although *O. vallartensis* sp. nov. was recovered as non-conspecific in this dataset. Among the newly proposed taxa, potential conspecificity was inferred between: (1) *O. peruvianus* and either *O. similaris* sp. nov. and/or *O. megapiculatus* sp. nov. in *12S rRNA* analyses; (2) *O. similaris* sp. nov. and *O. megapiculatus* sp. nov. in *16S rRNA* analyses; and (3) *O. variabilis* sp. nov. and *O. megapiculatus* sp. nov. in *12S rRNA* analyses.

## Discussion

*Oroperipatus* is among the most species-rich taxon of Peripatidae, yet its evolutionary history has remained poorly resolved. Herein, we bridged this gap by using an integrative, multimethod framework to investigate the evolutionary processes shaping its diversity and biogeography. Our results reveal that *Oroperipatus* exhibits substantially greater morphological diversity than previously recognized, with numerous diagnostic characters identified across all examined species. The expanded character set described herein underscores the taxonomic value of dorsal and ventral integumentary structures, as well as embryonic traits, for species delimitation. This approach led to the discovery and description of ten new species, increasing the known diversity of *Oroperipatus* from 18 to 28 valid species (∼50% increase) and substantially improving taxon sampling for evolutionary analyses. Molecular evidence was largely congruent with the morphological data: the morphological delimitation of *Oroperipatus* is consistent with the monophyly of the group, and the recognized morphotypes are recovered as distinct evolutionary lineages in phylogenetic reconstructions and as separate entities in single-locus species delimitation analyses (see Remarks on morphological characters and species delimitation in Document S1). Comparisons with previously described species were hindered by the limited availability of morphological information and, particularly, molecular data in the literature. Nevertheless, the unique combination of characters identified for each newly described species strongly suggests that conspecificity with any currently recognized nominal species is unlikely, as detailed in the respective differential diagnoses (Document S1).

Despite this morphological diversity, our results indicate that geographic proximity provides a stronger predictor of lineage relationships than morphological similarity—an idea previously proposed for other members of Neopatida.^27^^,^^41^ The phylogeny of *Oroperipatus* recovered here does not align with any discernible morphological pattern, nor does it conform to Bouvier’s^28^ taxonomic framework (Figure S36). Instead, *Oroperipatus* comprises two well-supported subclades geographically structured along the Pacific and Amazonian flanks of the Andes. A notable exception to the geographic-proximity pattern is the close relationship between geographically disjunct Mexican and Galápagos species within the Pacific subclade—a pattern consistently recovered across multiple molecular studies,^38^^,^^39^^,^^40^ suggesting it is not an analytical artifact.

A major obstacle to understanding the characteristic distribution of *Oroperipatus* has been the poorly resolved divergence times among its lineages, partly due to limited taxa representation.^38^^,^^39^ Consequently, whether Andean orogeny was the main driver of cladogenesis has remained unclear.^40^ Our molecular clock estimates indicate that *Oroperipatus* diverged from other Neopatida more recently than previously thought, likely during the Early Cretaceous in the Aptian ∼118 Ma, rather than the Late Triassic (237–201 Ma^40^) or Middle Jurassic (174–162 Ma^39^). This divergence predates the onset of Andean orogeny by ∼18 Ma, suggesting that Aptian global perturbations—characterized by shifts in plate tectonics, the carbon cycle, and ocean-climate systems, as well as a pronounced rise in global mean temperatures followed by cooling^49^—may have initiated diversification within this lineage rather than the Andean uplift. Notably, this interval coincides with the early diversification of angiosperms during the Early Cretaceous (139–93 Ma^50^^,^^51^), implicating vegetation restructuring as an additional driver of diversification.

Within *Oroperipatus*, we identify multiple cladogenetic events from the Early Cretaceous through the Pleistocene, each potentially associated with climatic, ecological, and/or geological transitions characteristic of its periods. Despite the strong phylogeographic structure of *Oroperipatus*, divergence between the Amazonian and Pacific subclades dates to ∼114 Ma, still predating the earliest mid-Cretaceous Andean uplift (∼100 Ma^9^). Thus, genetic differentiation between these subclades occurred at least 14 Ma prior to their geographic isolation by Andean orogenesis, possibly under the same Aptian-driven climatic and vegetation changes. In contrast, the earliest diversification events within each subclade were likely more directly influenced by uplift of the Northern and Central Andes.

The Andean uplift occurred diachronously—beginning in the Southern Andes (∼100 Ma), followed by the Northern (∼80 Ma) and Central Andes (∼70 Ma).^17^ This non-uniform orogeny complicates attempts to apply a single uplift timeline to explain diversification and biogeography in *Oroperipatus*, a challenge also recognized in various Andean plant groups.^17^ In Ecuador—which harbors the highest number of described *Oroperipatus* species—vicariant processes were likely major drivers of diversification. Uplift there commenced between 80 and 70 Ma,^17^ closely corresponding to the earliest divergences among Ecuadorian species within both Amazonian and Pacific subclades. However, the modern structure of the Ecuadorian Andes formed much later, over the past 20–30 million years,^8^^,^^15^ and our results likewise show an increasing number of cladogenetic events during this interval. Even Ecuadorian species that currently occur in sympatry, including *O. variabilis* sp. nov., *O. similaris* sp. nov., and *O. megapiculatus* sp. nov., exhibit deep divergence times (23–13 Ma) corresponding to this interval, indicating that their speciation was more likely driven by the Andean uplift than by alternative evolutionary mechanisms associated with sympatric speciation.

However, many *Oroperipatus* lineages only diversified during the Neogene (16–5 Ma), especially in Late Miocene, aligning with the accelerated Andean uplift over the past ∼10 Ma.^15^ Comparable Late Miocene radiations are observed in other Andean taxa, such as lizards, where rapid speciation reflects a complex interplay of vicariant, climatic, and ecological forces during the final stages of Andean orogeny.^15^ Late-stage uplift especially reshaped the Amazonian flank, altering landscapes, drainage systems, and sediment influx into the Amazon Basin^8^—geomorphological changes that profoundly impacted regional ecosystems and influenced diversification of various taxa.^17^ Although the Amazonian subclade of *Oroperipatus* remains poorly sampled^33^ and includes only five species in our dataset, the scenario outlined above suggests that this subclade contains substantial undocumented diversity, potentially exceeding that of the Pacific subclade.

One of the most intriguing aspects of *Oroperipatus* evolution is the disjunct distribution of lineages in the Galápagos and Mexico, and their close phylogenetic affinity despite the vast geographic distance. The occurrence of *Oroperipatus* in the Galápagos has been attributed to overwater dispersal,^38^ as the archipelago remained isolated from South American mainland since its emergence ∼5 Ma.^52^^,^^53^ Although the number of species on the islands and the details of colonization remain unknown, dispersal from mainland Ecuador—only ∼1,000 km away—is plausible and supported by the shared ancestry of Galápagos (plus Mexican) and Ecuadorian taxa.^38^ However, their estimated divergence from Ecuadorian lineages at ∼6 Ma slightly predates the Galápagos formation, potentially reflecting incomplete sampling, particularly along Ecuador’s western coast, the probable source of the colonizing population.

The evolutionary history of Mexican *Oroperipatus* is even more challenging to reconstruct. If northwestern South America—where *Oroperipatus* shows the greatest diversity and deepest divergence times—is considered the clade’s center of origin, the Mexican lineage represents a northward dispersal event. Two non-mutually exclusive dispersal scenarios may be hypothesized: (1) a continuous overland expansion through Central America, potentially associated with the Great American Biotic Interchange (GABI) approximately 3 Ma,^54^ and (2) an overwater dispersal along the Pacific margin of the Americas. The overland dispersal hypothesis is complicated by the presumable absence of *Oroperipatus* throughout most of Central America. Extensive onychophoran surveys in the region,^32^^,^^38^^,^^55^^,^^56^^,^^57^ have failed to document *Oroperipatus* from the eastern side of the Isthmus of Panama (i.e., the Central American side of the Panama Canal) northward to Guatemala. Although a single record exists from Belize,^58^ it is likely based on a misidentification.^38^^,^^39^

Consequently, the overland scenario would require either that *Oroperipatus* populations have remained undetected in Central America or that they underwent local extinction. Such extirpation could have resulted from ecological shifts over geological time that negatively affected habitat suitability, or even from competitive interactions with other ancient neopatid lineages that persist in the region.^38^ Nevertheless, the hypothesis remains plausible, as both incomplete sampling and regional extinction are common factors complicating the reconstruction of historical biogeographic patterns. The only confirmed Central American records of *Oroperipatus* are from the western side of the Isthmus of Panama (i.e., the South American side of the Panama Canal),^56^^,^^59^ indicating that *Oroperipatus* possibly reached this region through overland dispersal from Colombia, but since these species have not yet been examined phylogenetically, their evolutionary relationship to the Mexican species remains unresolved.

Alternatively, *Oroperipatus* could have colonized Mexico via overwater dispersal along the Pacific margin of the Americas. Our divergence-time estimates indicate that Mexican and Galápagos *Oroperipatus* diverged ∼4 Ma, a timepoint not corresponding to any known vicariant event involving these two areas. Consequently, this hypothesis would require dispersal either (1) via a series of ecologically suitable, closely spaced habitat patches (i.e., a stepping-stone or island-hopping model), or (2) through a long-distance colonization event (i.e., sweepstakes dispersal).^40^ The latter would entail transport on transient mobile ecosystems that act as vessels for ecological connectivity, such as floating vegetation mats or islands. While short-term persistence of onychophorans on such rafts cannot be entirely excluded, prolonged exposure to elevated temperatures and seawater conditions, both lethal to onychophorans,^60^ renders sustained long-distance dispersal improbable.

Even though continuous overland dispersal between South and North America became possible only after the closure of the Isthmus of Panama, evidence suggests earlier biotic exchanges between them. Panbiogeographical models propose an eastern “Greater Antillean arc”—foreland connections across the eastern Pacific—that could have enabled taxa exchanges between western South and North America ∼90–76 Ma.^52^ This model was previously used to explain disjunct yet closely related plant (e.g., *Gossypium*, *Galvezia*, *Gambelia*) and animal groups (e.g., the flightless *Rothium* beetles, iurid scorpions) that, like *Oroperipatus*, occur in Ecuador, the Galápagos, and Mexico.^52^^,^^61^^,^^62^^,^^63^^,^^64^^,^^65^ However, its timescale clearly predates the geological age of the Galápagos, while recent molecular clock estimates for *Gossypium* indicate that divergence times among Galápagos (*G. darwinii*), Mexican (*G. hirsutum*), and Ecuadorian (*G. barbadensis*) species are incompatible with dispersal via a “Greater Antillean arc”.^66^

Besides the Late Cretaceous (90–76 Ma), further biotic exchange between South and Central/North America may have occurred during the Oligocene-Miocene transition (∼23–20 Ma) and from the Late Miocene (∼8–6 Ma) to the Early Pliocene (5–3 Ma).^67^ The latter roughly coincides with the divergence of Galápagos and Mexican *Oroperipatus* lineages from their Ecuadorian relatives (∼6 Ma) and from each other (∼4 Ma). Late Miocene dispersal was likely driven by climatic fluctuations and eustatic sea-level changes, and facilitated by transient island chains or narrow land connections formed by emergent volcanic arcs along the Pacific Ring of Fire (scenario I in Figure 6).^67^ However, geological evidence supporting this insular system remains limited. Alternatively, independent overwater dispersal from mainland Ecuador to both the Galápagos^38^ and Mexico (scenario II in Figure 6) cannot be excluded, although a Late Miocene stepping-stone dispersal scenario appears comparatively more parsimonious.

**Figure 6:**
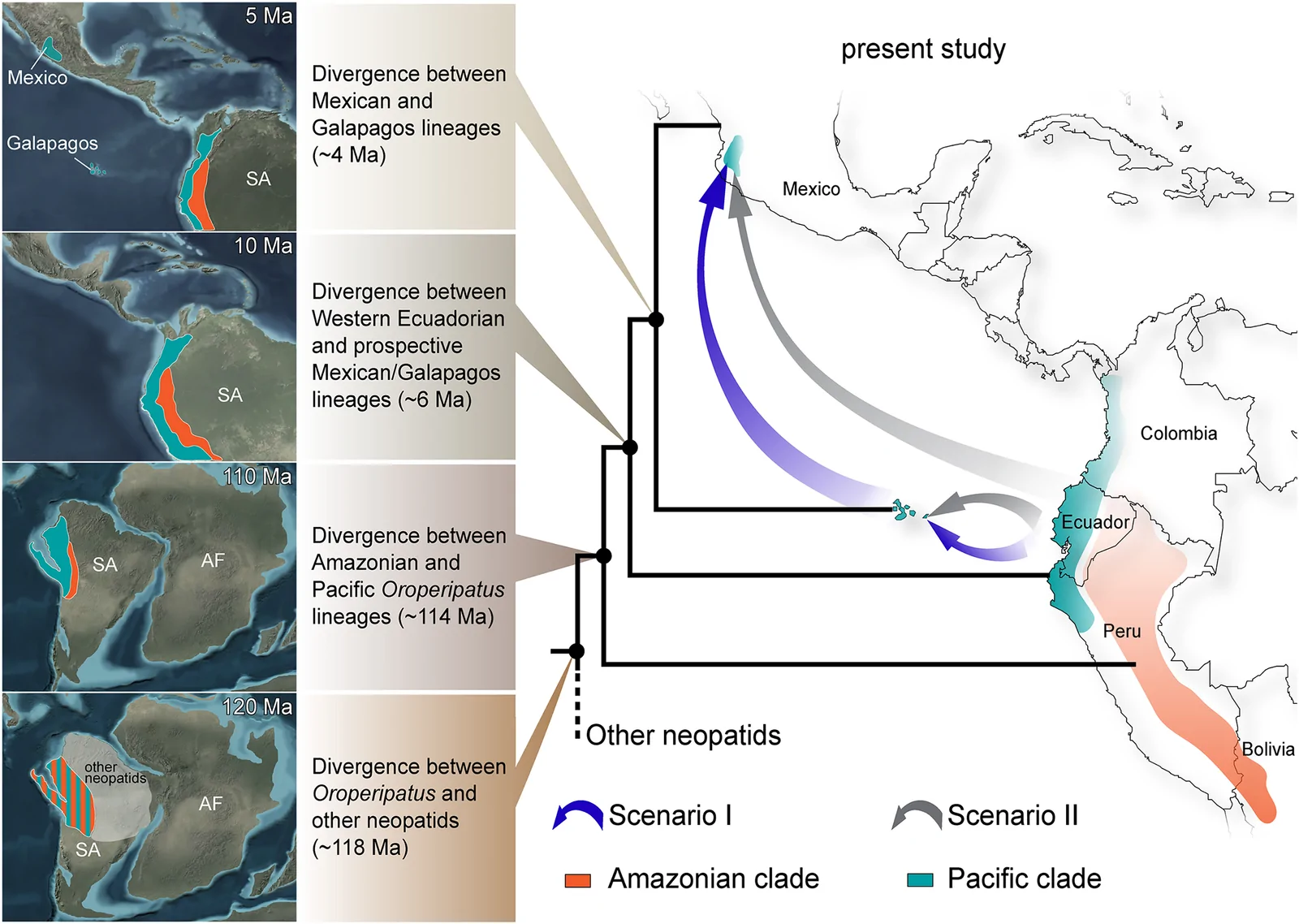
Proposed biogeographic scenarios based on divergence estimates Paleogeographic maps (left) indicate the likely geographic distribution of the Amazonian (orange) and Pacific (blue) subclades during main cladogenetic events within *Oroperipatus*. Present-day map (right) shows two possible colonization scenarios for the Galápagos and Mexico from western Ecuador. Scenario I (purple arrows) implies stepwise northward dispersion to Mexico via the Galápagos, while scenario II (gray arrows) assumes the independent colonization of these regions. AF, Africa; SA, South America.

Assuming this Late Miocene event shaped the present-day distribution of *Oroperipatus* in the Galápagos and Mexico, a pioneer Ecuadorian lineage may have colonized the Galápagos soon after the islands emerged at the onset of the Pliocene (5–3 Ma). As Bacon et al.^10^ suggest, the Late Miocene dispersal pulse likely continued into the Early Pliocene, potentially enabling further stepwise movement from the Galápagos northwestward to southern Mexico. Although the timing and position of ephemeral Pacific islands and possible land bridges remain uncertain, such an island-hopping scenario is broadly consistent with divergence estimates recovered herein and in previous studies.^38^^,^^39^ Nevertheless, the apparent phylogenetic affinity between the Mexican and Galápagos *Oroperipatus* lineages may also reflect incomplete taxon sampling, with more closely related extant or unsampled lineages absent from current datasets. Expanded finer-scale geographic sampling across *Oroperipatus*’ distribution range will therefore be essential to test this hypothesis, refine divergence time estimates, and evaluate alternative biogeographic scenarios. At present, the hypotheses proposed here represent plausible explanations for the inferred sister-group relationship between the Mexican and Galápagos species.

In conclusion, *Oroperipatus* diversity and biogeography have been shaped by a complex interplay of climatic fluctuations, ecological and vegetational shifts, and vicariant events spanning the Early Cretaceous to the present. Early cladogenetic events were likely driven by paleoclimatic oscillations, while Andean uplift promoted allopatric speciation within both subclades on South American mainland. Simultaneously, sea-level changes and climatic shifts, along with transient island chains and land bridges during the Miocene-Pliocene, facilitated the stepwise dispersal of Pacific *Oroperipatus* to the Galápagos and southern Mexico. Though masked by morphology, these processes remain evident in the molecular diversity of *Oroperipatus*, highlighting the importance of an integrative, multi-method analytical framework, still underused in evolutionary studies of Onychophora.

### Limitations of the study

Morphological and molecular data remain limited for most nominal taxa currently assigned to *Oroperipatus*, constraining robust comparisons with the new species described herein. Comprehensive characterization of these taxa using integrative morphological and molecular approaches is needed to improve species delimitation, clarify phylogenetic relationships, and refine inferences of their biogeographic history and divergence times. However, such analyses are currently difficult to undertake using historical type material because of specimen age, restrictions on invasive sampling imposed by museum collections, and the limited availability of specimens. The exclusive reliance on collection-based material in the present study also imposed substantial limitations on morphological and molecular assessments. Preservation artifacts occasionally obscured key morphological features, while molecular analyses were constrained by DNA degradation and, in some cases, low extraction yields. These limitations emphasize the challenges inherent to incorporating historical specimens into integrative systematic studies, particularly when both morphological and molecular data are required. Future studies should therefore prioritize targeted field sampling at the type localities of *Oroperipatus* species and the collection of topotypical material. Access to freshly collected specimens would facilitate comprehensive morphological and molecular characterization using modern approaches in onychophoran research.

## Resource availability

### Lead contact

Requests for further information and resources should be directed to and will be fulfilled by the lead contact, José Pablo Barquero-González (jopbgon@student.uni-kassel.de).

### Materials availability

This study did not generate new unique reagents.

### Data and code availability

• DNA gene sequence data have been deposited at the National Center for Biotechnology Information (NCBI) as GenBank: accession number (key resources table and Table S2 of Document S1) and are publicly available as of the date of publication.
• This paper analyzes existing, publicly available data, accessible at GenBank: accession number (key resources table and Table S2 of Document S1).
• Electronic Scanning Microscope micrograph data reported in this paper will be shared by the lead contact upon request.
• This paper does not report original code.
• Any additional information required to reanalyze the data reported in this paper is available from the lead contact upon request.

## Acknowledgments

G.M. thanks Maik Dobiey for kindly providing specimens of *Oroperipatus peruvianus*. We are grateful to Lars Hering for his assistance with molecular data and analyses, and to Janet Beccaloni for granting access to the Onychophora collection at the Natural History Museum of London (United Kingdom). We also thank Katrina Karemoov (@Kareemov1000) for kindly allowing the use of her Andes artwork in Figures 3 and S10. This study was supported by the 10.13039/100021828German Academic Exchange Service (DAAD: 91799767) to J.P.B.-G. and by the 10.13039/501100003593Conselho Nacional de Desenvolvimento Científico e Tecnológico (CNPq, Brazil: 290029/2010–4) to I.d.S.O. This work was further supported by the 10.13039/501100001659Deutsche Forschungsgemeinschaft (DFG), Research Unit REASSEMBLY (FOR 5207; sub-project SP2 HE 4593/12-1). The research and collection permits were granted through Contrato Marco MAE-DNB-CM-2021-0187 by the 10.13039/100019129Ministry of Environment of Ecuador. We thank Martin Schaefer and Citlalli Morelos-Juarez for allowing research in their reserves, Juan Ernesto Guevara Andino and Sebastián Escobar for handling export permits, and Karla Neira and MO Rödel for collaboration in SP2.

## Author contributions

J.P.B.-G., conceptualization, resources, methodology, formal analysis, data curation, writing – original draft, and writing – review and editing. V.M.S.J.R., G.M.-P., G.C.-F., A.T., F.V., and M.H., resources and writing – review and editing. G.M., conceptualization, project administration, resources, funding acquisition, supervision, and writing – review and editing. I.d.S.O., conceptualization, formal analysis, methodology, data curation, supervision, writing – original draft, and writing – review and editing. All authors discussed the results, read, and approved the final version of the manuscript.

## Declaration of interests

The authors declare no competing interests.

## Declaration of generative AI and AI-assisted technologies in the manuscript preparation process

No AI or AI-assisted technologies were used during the preparation of this manuscript.

## STAR★Methods

### Key resources table

**Table:** 

REAGENT or RESOURCE	SOURCE	IDENTIFIER
**Bacterial and virus strains**		

*Escherichia coli* KJ100 strain	Universität Kassel, Kassel; Germany	N/A

**Biological samples**		

*Oroperipatus megapiculatus*; *COI*	This study	GenBank: PX876303
*Oroperipatus megapiculatus*; *18S*	This study	GenBank: PX746784
*Oroperipatus megapiculatus*; *16S*	This study	GenBank: PX746774
*Oroperipatus megapiculatus*; *18S*	This study	GenBank: PX880762
*Oroperipatus lankesteri*; *16S*	This study	GenBank: PX972671
*Oroperipatus palenquensis*; *COI*	This study	GenBank: PX876300
*Oroperipatus palenquensis*; *12S*	This study	GenBank: PX746779
*Oroperipatus palenquensis*; *16S*	This study	GenBank: PX746769
*Oroperipatus palenquensis*; *18S*	This study	GenBank: PX880758
*Oroperipatus peruvianus 12S*	This study	GenBank: PX746780
*Oroperipatus peruvianus 16S*	This study	GenBank: PX746770
*Oroperipatus peruvianus 18S*	This study	GenBank: PX880759
*Oroperipatus sachaensis; COI*	This study	GenBank: PX876298
*Oroperipatus sachaensis; 12S*	This study	GenBank: PX746777
*Oroperipatus sachaensis; 16S*	This study	GenBank: PX746767
*Oroperipatus sachaensis; 18S*	This study	GenBank: PX880756
*Oroperipatus similaris; COI*	This study	GenBank: PX876302
*Oroperipatus similaris; 12S*	This study	GenBank: PX746783
*Oroperipatus similaris; 16S*	This study	GenBank: PX746773
*Oroperipatus similaris; 18S*	This study	GenBank: PX880761
*Oroperipatus specialis; COI*	This study	GenBank: PX876297
*Oroperipatus specialis; 12S*	This study	GenBank: PX746776
*Oroperipatus specialis; 16S*	This study	GenBank: PX746766
*Oroperipatus specialis; 18S*	This study	GenBank: PX880755
*Oroperipatus vallartensis; COI*	This study	GenBank: PX876299
*Oroperipatus vallartensis; 12S*	This study	GenBank: PX746778
*Oroperipatus vallartensis; 16S*	This study	GenBank: PX746768
*Oroperipatus vallartensis; 18S*	This study	GenBank: PX880757
*Oroperipatus variabilis; COI*	This study	GenBank: PX876301
*Oroperipatus variabilis; 12S*	This study	GenBank: PX746781
*Oroperipatus variabilis; 16S*	This study	GenBank: PX746772
*Oroperipatus variabilis; 18S*	This study	GenBank: PX880760
*Oroperipatus variabilis; COI*	This study	GenBank: PX956827
*Oroperipatus variabilis; 12S*	This study	GenBank: PX972669
*Oroperipatus variabilis; 16S*	This study	GenBank: PX972670
*Oroperipatus variabilis; 18S*	This study	GenBank: PX957499
*Oroperipatus eisenii; COI*	This study	GenBank: PX876296
*Oroperipatus eisenii; 12S*	This study	GenBank: PX746775
*Oroperipatus eisenii; 16S*	This study	GenBank: PX746765
*Oroperipatus eisenii; 18S*	This study	GenBank: PX880754

**Critical commercial assays**		

DNeasy Blood and Tissue extraction kit	Qiagen, Hilden, Germany	69504
pGEM®-T Vector System	Promega Corporation, Madison, WI, USA	A3600

**Deposited data**		

Publicly available Onychophora data	NCBI GenBank	Refer to Table S2 (Document S1)

**Software and algorithms**		

RAxML 7.3.0 PTHREADS-SSE3	https://github.com/stamatak/standard-RAxML	RRID:SCR_006086
MAFFT	https://mafft.cbrc.jp/alignment/server/	RRID:SCR_011811
Gblocks 0.91b	http://phylogeny.lirmm.fr/phylo_cgi/one_task.cgi?task_type=gblocks	RRID:SCR_015945
bPTP server: a Bayesian implementation of the PTP model for species delimitation	https://species.h-its.org/ptp/	RRID: SCR_003415
ASAP Species Delimitation	https://spartexplorer.mnhn.fr/delimitation	RRID: SCR_001849
BEAUti	https://github.com/CompEvol/beast2/releases	RRID:SCR_017307
BEAST 2.4.7	https://github.com/CompEvol/beast2/releases	RRID:SCR_017307
Adobe Photoshop 2023	Adobe, San Jose, CA, USA	RRID:SCR_014199
Adobe Illustrator 2023	Adobe, San Jose, CA, USA	RRID:SCR_010279
Google Earth Pro	https://www.google.com/earth/about/versions/#download-pro	RRID:SCR_023224

**Other**		

Stereomicroscope	Stemi 508, Carl Zeiss, Jena, Germany	N/A
Critical point dryer	CPD 030 BAL-TEC AG, Balzers, Liechtenstein; and K850, Emitech Ltd., Kent, England	N/A
SCD 050 Sputter Coater	BALZERS UNION, Balzers, Liechtenstein	N/A
Hitachi S-4000 field emission scanning electron microscope	Hitachi High-Technologies Europe GmbH, Krefeld, Germany	N/A
Agilent 2100 Bioanalyzer	Agilent Technologies Inc., Santa Clara, CA, USA	N/A
Genome Analyzer IIx platform	Illumina, San Diego, CA, USA	N/A
CLC Genomics Workbench 4.7.2	CLC Bio, Aarhus, Denmark	N/A

### Experimental model and study participant details

The analyzed specimens originated either from the scientific collections of the University of Kassel in Germany, the Natural History Museum in London, UK and the Museo de Zoología de la Universidad de Costa Rica in Costa Rica, or were collected from Ecuador in 2022 under the permit number MAATE-DBI-CM-2021–0187 issued by the Ministerio del Ambiente, Agua y Transición Ecológica, and from Mexico under the permit number FAUT-0209 issued by the Subsecretaría de Gestión para la Protección Ambiental, Dirección General de Vida Silvestre (Table S1). In total, 27 specimens from 11 distinct localities were examined (Figure 1; Table S1). Missing elevation data were retrieved using Google Earth Pro based on recorded locality information. Type specimens, including scanning electron microscopy (=SEM) stubs containing their parts, were deposited in the scientific collections of the Museo de Zoología de la universidad Católica de Ecuador (QCAZI), Quito, Ecuado; the Colección Nacional de Ácaros de la Universidad Nacional Autónoma de México (CNCA), Mexico City, Mexico; the Museo de Zoología de la Universidad de Costa Rica (MZUCR), San José, Costa Rica; and the Aracnology Collection of the Museum of Nature Hamburg (ZMH-ARA), Hamburg, Germany (Table S1).

### Method details

#### Morphological data

Preserved specimens were measured with calipers and examined under a stereomicroscope (Stemi 508, Carl Zeiss, Jena, Germany) to assess diagnostic characters of *Oroperipatus* and to determine the number of leg pairs (Table S1). A topotype of the genus type species *Oroperipatus lankesteri* deposited in The Natural History Museum, London was analyzed exclusively under a stereomicroscope due to the museum’s restrictions. For scanning electron microscopy, selected specimens were cut into smaller parts containing taxonomically relevant features; late-stage embryos found inside dissected females were extracted and prepared as well. Sample preparation protocol followed Oliveira et al.,^35^ including dehydration in a graded ethanol series (70, 80, 90, 95, 2 × 100%; 15 min each), drying with a critical point dryer (CPD 030 BAL-TEC AG, Balzers, Liechtenstein; and K850, Emitech Ltd., Kent, England), mounting on standard sample holders, and coating with gold-palladium using an SCD 050 Sputter Coater (BALZERS UNION, Balzers, Liechtenstein). For species represented by a single available specimen, a midbody piece containing several segments was preserved in ethanol as a voucher. Imaging was conducted using a Hitachi S-4000 field emission scanning electron microscope (Hitachi High-Technologies Europe GmbH, Krefeld, Germany). Morphological terminology is used according to Reid^63^ and Oliveira et al.^68^^,^^69^

#### Molecular data

Total genomic DNA was extracted from muscle tissue of the studied specimens using a DNeasy Blood and Tissue extraction kit (Qiagen, Hilden, Germany), following the manufacturer’s protocol. The final concentration of each DNA sample was measured using either a NanoDrop 2000 (Thermo Fisher Scientific Inc., Waltham, MA, USA) or a Qubit 3.0 Fluorometer (Invitrogen, Life Technologies, Waltham, MA, USA). The extracted DNA was processed for either whole-genome shotgun library preparation or Sanger sequencing of the target genes: the mitochondrial *COI*, *12S rRNA* and *16S rRNA* genes, and the nuclear *18S rRNA* gene.

Whole-genome shotgun sequencing was performed after assessing DNA quality using an Agilent 2100 Bioanalyzer (Agilent Technologies Inc., Santa Clara, CA, USA). Libraries were prepared using a multiplex protocol with double-indexing modifications,^70^^,^^71^ and sequenced on a Genome Analyzer Iix platform (Illumina, San Diego, CA, USA). The sequences were assembled *de novo* using CLC Genomics Workbench 4.7.2 (CLC Bio, Aarhus, Denmark) as described elsewhere.^35^^,^^72^ Mitochondrial gene sequences for target genes were retrieved from the assembled libraries using BLAST searches.^73^ For Sanger sequencing, the following primers were used to amplify gene fragments: ony_COI_F63 (GRWTRTTYTCWACWAATCATAARGATATTGGDAC), ony_COI_R63 (CWGGATAATCHGAATAYCGHCGNGGYA), LCO1490_COI (GGTCAACAAATCATAAAGATATTGG), and HCOoutout_COI (GTAAATATATGRTGDGCTC) for *COI*; ony_12S_F69 (TTCGTGCCAGCAGYWGCGGTTAWAC) and ony_12S_R63 (TTTCCAKTRNDYCTACTWTGTTWCGACTT) for *12S rRNA*; ony_16S_F63 (TTCAWCTGTTTAWCAAAAACATWGYTTT) and ony_16S_R65 (GATAGAAACCAAYCTGGYTYWMRYCG) for *16S rRNA*; and ony_18S_F68 (CATACGCTCGTCTCAAAGATTAAGCCAT) and ony_18S_R69 (GGTAGTAGCGACGGGCGGTGTGTA) for *18S rRNA*. Gaps in the *18S rRNA* fragments were closed using the primers 18S_seq_F2 (GCATGGCCGTTCTTAGTTGG) and 18S_seq_R2 (CCAACTAAGAACGGCCATGC). The PCR products were cloned into the *Escherichia coli* KJ100 strain using the pGEM-T Vector System (Promega Corporation, Madison, WI, USA). Viable clones ranging from *n* = 5 to *n* = 60 were selected and sent to Eurofins Genomics Europe Shared Services GmbH (Ebersberg, Germany) for sequencing. Corresponding sequences of additional species were retrieved from GenBank (Table S2). Sequences were aligned using the online interface of MAFFT^74^ with the E-INS-i strategy for the mitochondrial genes and the G-INS-i strategy for the nuclear gene, and the resulting alignments were trimmed with Gblocks 0.91b.^75^

Molecular diagnostic characters for *Oroperipatus* and its two major clades were inferred from *18S rRNA* gene sequences following Sato et al.,^76^ using the most inclusive outgroup configuration. The final *18S rRNA* sequence alignment was examined in BioEdit v. 7.0.9.0^77^ using the “shade identities and similarities in alignment window” option, and sites of interest were identified *a posterior* based on the screening of the alignment. Newly generated sequences were deposited in GenBank and assigned with specific accession numbers (Table S2).

#### Phylogenetic analyses

Maximum likelihood inference was conducted on concatenated alignments, varying only the treatment of the *COI* gene partition as either (i) all nucleotide sites, or (ii) nucleotide sites with third codon position removed, or (iii) nucleotide sites translated to amino acids (Table S3). *COI* amino acid alignments were verified *a priori* with TranslatorX.^78^ Since only *16S rRNA* sequences were obtained from *O. lankesteri*, each matrix was analyzed twice, with and without this species (Table S3). Two alternative outgroup configurations were employed. The more inclusive dataset comprised representatives of Peripatopsidae, along with peripatid taxa from Southeast Asia (*Eoperipatus*), tropical Africa (*Mesoperipatus*), and multiple representatives of the Caribbean clade of Neopatida. To evaluate the potential impact of long-branch attraction associated with highly derived taxa (e.g., *Eoperipatus*), as well as biases arising from the limited and incomplete sampling of peripatopsids, a reduced dataset was constructed. This simplified dataset included only Caribbean neopatids and their immediate sister taxon (*Mesoperipatus*) as outgroup. Maximum likelihood analyses were performed in RAxML 7.3.0 PTHREADS-SSE3,^79^ using GTR+G+I substitution model for nucleotide and MTART+G+I for amino acid partitions.^80^ Node support was assessed with 1,000 bootstrap pseudoreplicates.^81^ Trees were visualized online with the Interactive Tree of Life (iTOL) platform.^82^

#### Species delimitation

To provide additional line of evidence supporting the proposed species designation, we conducted single-locus species delimitation analyses using the Bayesian implementation of the Poisson Tree Processes model^83^ (bPTP) on both maximum likelihood (bPTP-ML) and Bayesian inference (bPTP-BI) trees, as well as Assemble Species by Automatic Partitioning (ASAP).^84^ Maximum-likelihood (ML) phylogenetic reconstructions were generated separately for each mitochondrial marker (*12S rRNA*, *16S rRNA*, and *COI*), and the resulting gene trees were used as input for species delimitation analyses implemented on the PTP online server (http://species.h-its.org/ptp/). For each topology, analyses were run for 500,000 generations using rooted trees, and both ML and BI species delimitation outputs were obtained. The ASAP analyses were conducted on the Spart Explorer online server (https://spartexplorer.mnhn.fr/delimitation) with default parameters and using the nucleotide alignments of each gene as input; the highest supported partition resulting from the best 10 reported by the analysis was selected.

#### Divergence-time estimates and fossil calibration

Divergence times were estimated using a calibrated molecular clock following Giribet et al.^38^ and considered the more inclusive outgroup configuration. The analyses were based on Bayesian inference using concatenated nucleotide sequences of all target genes for 67 species, without *O. lankesteri*. Calibration was performed in BEAUti 2,^85^ using the oldest known onychophoran fossil †*Antennipatus montceauensis*^86^ as the first calibration point. This fossil constrained the divergence between Peripatidae and Peripatopsidae to a minimum age of 298.9 ± 0.15 Ma,^87^ we assigned a normal distribution with a mean = 298.75 Ma and a sigma of ten (=30 Ma) to the root of Onychophora. A second calibration point was set using the peripatid fossil †*Cretoperipatus burmiticus*^88^ from the Cenomanian of Myanmar (maximum age: 98.79 ± 0.62 Ma^89^). Herein used to constrain the divergence between the Southeast Asian and Western Gondwanan Peripatidae clades, by applying a log-normal distribution with an offset at 98.79 Ma and S = 2.2 (97.5% quartile extending to 300 Ma). Convergence, burn-in parameters, posterior probabilities, and effective sample size (ESS) values were assessed using Tracer 1.7.2.^80^ Post-burn-in trees from multiple runs were merged in LogCombiner 1.10.4, and a summary tree was generated in TreeAnnotator 1.10.4 (both part of the BEAST package), using median node heights and the maximum clade credibility tree. Node dating was conducted using the Bayesian Evolutionary Analysis Sampling Trees 2.4.7 software (BEAST)^86^ using the substitution model averaging from bModelTest,^90^ a relaxed clock log-normal model, and a birth-death speciation model. The analysis ran for 50,000,000 generations, sampling one tree every 50,000 generations; 10% of the sampled trees were discarded as burn-in. Stratigraphic nomenclature follows the International Chronostratigraphic Chart (v 2023/9) provided by the International Commission on Stratigraphy.^91^

#### Graphic design

Brightness and contrast adjustments for scanning electron micrographs were performed using Adobe Photoshop 2023 (Adobe, San Jose, CA, USA). Maps and final panels were created using Adobe Illustrator 2023.

#### Nomenclatural acts

This study and its nomenclatural acts have been registered in ZooBank as recommended by the International Commission on Zoological Nomenclature (ICZN). ZooBank Life Science Identifiers (LSIDs) can be accessed by adding the LSID to the URL prefix “http://zoobank.org/”. The LSID for this publication is: urn:lsid:zoobank.org:pub:8CF9D215-0586-476A-82AF-C1E37AE774D7.

### Quantification and statistical analysis

Quantitative analyses are descriptive, as detailed in the method details section. The final alignments used for molecular analyses comprised 2,470–2,863 positions. Divergence times were estimated using a Bayesian framework implemented in BEAST v2.4.7. Maximum-likelihood phylogenetic analyses were conducted using RAxML v7.3.0 (PTHREADS-SSE3), species delimitation analyses were performed using the Bayesian Poisson Tree Processes (bPTP) model and Assemble Species by Automatic Partitioning (ASAP), both implemented through their respective online servers. Maximum-likelihood analyses employed the selected nucleotide substitution model, and node support was evaluated using 1,000 bootstrap pseudoreplicates. All bootstrap support values are reported on the phylogenetic trees. All results are presented in the main article and the accompanying Document S1.
