# Supplementary material for: Allopatric speciation of Oroperipatus velvet worms driven by Andean orogenesis and paleoclimate
Source: iScience. 2026 Nov 10;29(11):118189. doi: 10.1016/j.isci.2026.118189 (PMC13770711; doi:10.1016/j.isci.2026.118189)
Supplement: Supplementary file 1 [file mmc1.pdf]

iScience, Volume *29*

Supplemental information

Allopatric speciation of *Oroperipatus* velvet worms driven by Andean orogenesis and paleoclimate

José Pablo Barquero-González, V. Morley St. J. Read, Griselda Montiel-Parra, Gerardo Contreras-Félix, Arianna Tartara, Fernando Villagomez, Michael Heethoff, Georg Mayer, and Ivo de Sena Oliveira

Document S1

Supplementary information

SUPPLEMENTARY TAXONOMY

This section supplements the descriptions in the main text by providing expanded etymologies, characterizations of non-diagnostic features, and differential diagnoses for all described species. The differential diagnoses exclude the nomina dubia *O. goudoti*, *O. peruanus*, and *O. quitensis* (see Oliveira [S1]).

Oroperipatus eisenii **(Wheeler, 1898** [S2]**)**

Coloration: Preserved specimen without pattern, dorsally dark greyish brown, ventrally pale; cephalic white collar absent.

Measurements: Body size in 70% ethanol: length 331 mm, width 29.5 mm, height 32 mm.

Antennae: Thirty-six complete antennal rings, including terminal button. Antennal body with 24 rings; antennal tip with 12 rings + terminal button; 8th, 10th, and 12th rings of tip thinner, lacking chemoreceptors. Chemoreceptors round, surrounded by 4–5 scales (Figure S22A). Type I antennal sensillae in all rings except terminal button, with semi-elliptical, two-ranked apical piece and sensory bristle with structured base (Figure S23A). Type II antennal sensillae restricted to antennal tip, with conical structured base (Figure S24A). Antennal sensory field on 20 proximal-most, complete rings and five interpolated incomplete rings. Spindle-shaped sensillae elliptical, with 2–3 ranks of short, textured scales equal in width and length; terminal bristle blunt-ended (Figure S25A). Frontal organs elongated, ventrolateral at each antennal base; with long modified scales at internal surface (Figure S26A).

Head: One pair of slime papillae; smaller median lobes with one prominent and 1–2 smaller dermal papillae, or lacking dermal papillae; larger lateral lobes with three prominent dermal papillae each (Figure S27A). Eyes pigmented, well-developed, ~80 µm diameter (Figure S28A). Mouth with two lip rows: internal row with seven large lip pairs + one unpaired anterior lip; external row with 12 small lip pairs, variable shape/size (Figure S29A).

Dorsal integument: Twelve complete plicae per segment; seven extend ventrally between leg pairs. Dorsomedian furrow distinct throughout body. Hyaline organs paired, irregular, covered with structured cuticle. Primary and accessory papillae in repeated pattern along dorsal midline: one accessory papilla flanked by one primary papilla on each side of dorsomedian furrow (Figure S30A). Dorsal primary papillae similar in shape/size, round bases with 2–3 scale ranks (Figure S14A); basal and apical pieces separated by inconspicuous constriction. Apical piece with terminal bristle thorn-shaped, size variable. One to four accessory papillae between primary papillae. Peripheral accessory papillae, small, round or rosette-like (Figure S30A).

Ventral integument: Type I crater-shaped papillae oval, with or without rudimentary apical piece; rudimentary apical piece lacking sensory bristle; apical collar with 5 scales (Figure S31A). Type II crater-

shaped papillae oval, similar in size, without rudimentary apical piece; arranged in ventral and ventro-lateral rows of six papillae between adjacent legs; apical collar with 9–10 scales (Figure S32A). Preventral and ventral organs between leg pairs along ventral midline (except genital segment), separated by one row of papillae. Genital pad between penultimate leg pair, covered with scales; genital opening slit-like in female (Figure S33A). Terminal anus.

Leg: Examined female with 28 leg pairs (Table S1). First and last leg pairs reduced; last pair rotated posteriorly. Penultimate leg pair larger than last, smaller than others. Four spinous pads on developed legs (Figure S34A); two on last pair. Spinous pads nearly straight, increasing proximally, covering about half ventral leg surface; fourth pad thinner, smaller, about 2/3 of third pad. Spines needle-shaped; conical, textured base about half spine length (Figure S34A with inset). Five transverse rings per leg, separated by thinner, incomplete rings (Figure S19A). Bean-shaped papillae dorsodistal on legs, in pouch of two tegumental folds; pouch closed in all legs. Nephridial tubercle on fourth, fifth leg pairs, on third spinous pad, dividing it into small anterior and large posterior parts (Figure S11A); tubercle attached to posterior part, not indenting adjacent pads. Eversible coxal vesicles on all legs except 4th, 5th, penultimate and last pairs. Four distal foot papillae per foot: two anterior, two posterior; distal papillae with tall, cylindrical-oval apical piece covered with fine, point scales and equipped with short blunt-ended sensory bristle (Figure S12A). Proximal setiform ridges with two bristles; distal ridges with one (Figure S35A).

Differential diagnosis: *Oroperipatus eisenii* can be distinguished from the *Oroperipatus* species examined in the present study by the unique combination of characters outlined in the diagnostic section. It can additionally be distinguished from previously described species of *Oroperipatus* as follows. The presence of 12 complete dorsal plicae per segment separates *O. eisenii* from *O. bimbergi*, *O. cameranoi, O. intermedius*, *O. koepckei*, *O. lankesteri*, *O. soratanus*, *O. tiputini*, *O. tuberculatus* and *O. weyrauchi*. The number and arrangement of distal foot papillae, consisting of two anterior and two posterior papillae, distinguish it from *O. bluntschlii*, *O. ecuadorensis*, and *O. lankesteri*. Females of *O. eisenii* exhibit more leg pairs than *O. omeyrus* but fewer than the remaining congeners, whereas the number of leg pairs in males overlaps only with that of *O. belli*, *O. bimbergi*, and *O. corradi*. Finally, the presence of only four complete spinous pads distinguishes *O. eisenii* from *O. bimbergi*, *O. bluntschlii*, *O. ecuadorensis*, *O. lankesteri*, *O. tiputini*, *O. tuberculatus*, and *O. weyrauchi*.

***Oroperipatus peruvianus* (Brues, 1917 [S3])**

Coloration: Preserved specimens dorsally dark brown without a pattern, ventrally beige; cephalic collar white, complete.

Measurements: Body size in 100% ethanol: length 315–531 mm, width 52–74 mm, height 34–43 mm.

Antennae: Forty-four complete antennal rings, including terminal button; antennal body with 30 rings; antennal tip with 13 rings + terminal button; 8th, 10th, 12th rings of tip thinner, without chemoreceptors. Chemoreceptors round, with 4–5 scales around (Figure S22B). Type I sensilla in all rings except terminal button, with conical, one-ranked apical piece; blunt-ended sensory bristle with structured base (Figure S23B). Type II sensilla restricted to antennal tip, with cylindrical structured base (Figure S24B). Antennal sensory field on 21 proximal-most, complete rings and five interpolated incomplete rings.

Spindle-shaped sensilla cylindrical to elliptical, with 2–3 ranks of long textured scales; blunt-ended terminal bristle (Figure S25B). Frontal organs elongated, ventrolateral at antennal base (Figure S26B).
Head: One pair of slime papillae; smaller median lobes lacking dermal papillae; larger lateral lobes with a single lateral dermal papilla each (Figure S27B). Eyes pigmented, well-developed, ~100 μm diameter (Figure S28B). Mouth with two rows of lips: internal row with seven large lip pairs + an unpaired anterior lip, upper four lips and unpaired lip bottle-like; external row with 11 pairs of small, variable lips (Figure S29B). Head pattern irregular.
Dorsal integument: Twelve complete plicae per segment; seven extend ventrally between leg pairs. Alternate pattern of thin and thick plicae dorsally: thick plicae with largest and mid-sized primary papillae + accessory papillae, followed by thin plicae with smallest primary papillae + accessory papillae (Figure S30B). Dorsomedian furrow distinct throughout body. Hyaline organs paired, round, with 7–12 structured, flat scales. Primary papillae with similar shape; basal piece round to oval, with 6–8 scale ranks in largest, 4–6 in mid-sized and 2–6 in smallest primary papillae (Figure S14B). Basal and apical piece separated by constriction. Terminal sensory bristle on apical piece thorn-shaped, variable size. Accessory papillae variable in size, round to oval, with 2–7 scale ranks (Figure S15B). One to three accessory papillae between primary papillae. Peripheral accessory papillae, absent.
Ventral integument: Type I and II crater-shaped papillae without rudimentary apical piece. Type I crater-shaped papillae round, apical collar with 7–11 scales (Figure S31B). Type II crater-shaped papillae elongated, apical collar with 9–12 scales (Figure S32B). One pair of interpedal structures per segment. Preventral and ventral organs between leg pairs along ventral midline (except genital segment), separated by two rows of papillae. Genital pad between penultimate leg pair, covered with scales. Genital opening cruciform in males (Figure S20A), slit-like in females (Figure S33B). Male anal gland openings paired, median, longitudinal (Figure S20B). Terminal anus.
Leg: Examined males with 29 leg pairs; females with 35 (Table S1). Last leg pair reduced, rotated posteriorly. First and penultimate leg pairs similar in size, larger than last, smaller than remaining legs. Five spinous pads on developed legs (Figure S34B), three on penultimate, 2–3 on last. Spinous pads arched, increasing proximally, covering ~1/3 of ventral leg surface. Fourth pad smaller than third; fifth pad smaller and thinner than fourth. Spines of spinous pads needle-shaped, with cylindrical, textured base ~1/3 of spine length (Figure S34B with inset). Seven transverse rings per leg, separated by thinner, incomplete rings (Figure S19B). Nephridial tubercle on fourth and fifth leg pairs, inserted on third spinous pad, dividing it into small anterior and large posterior parts, attached to larger part, not indenting adjacent pads (Figure S11B). Eversible coxal vesicles on all legs except 4th, 5th, penultimate and last pairs. Four distal foot papillae per foot: two anterior, two posterior; papillae with conical to cylindrical base, cylindrical apical piece and short, blunt-ended sensory bristle (Figure S12B). Proximal setiform ridges with two bristles; distal ridges with 1–2 (Figure S35B). Two prominent crural tubercles per leg on two pregenital segments in males (Figure S20C). Crural tubercle conical to cylindrical, with smooth-surfaced apices; opening slit-like (Figure S20D).
Differential diagnosis: *Oroperipatus peruvianus* can be distinguished from the *Oroperipatus* species examined in the present study by the unique combination of characters outlined in the diagnostic section. It can additionally be distinguished from previously described species of *Oroperipatus* as

follows. The 12 complete dorsal plicae per segment differentiates it from *O. bimbergi*, *O. cameranoi, O. intermedius*, *O. koepckei*, *O. lankesteri*, *O. soratanus*, *O. tiputini*, *O. tuberculatus*, and *O. weyrauchi.* The number and arrangement of distal foot papillae, consisting of two anterior and two posterior papillae, distinguish it from *O. bluntschlii*, *O. ecuadorensis*, and *O. lankesteri*. Females of *O. peruvianus* possess fewer leg pairs than females of *O. belli*, *O. bluntschlii*, *O. ecuadorensis*, *O. lankesteri*, *O. tiputini*, *O. tuberculatus* and *O. weyrauchi*, but more leg pairs than those of most remaining congeners, with the exception of *O. cameranoi*, which exhibits overlapping leg-pair counts. Finally, the presence of five complete spinous pads distinguishes *O. peruvianus* from *O. balzani*, *O. belli*, *O. cameranoi*, *O. corradi*, *O. intermedius*, *O. koepckei*, *O. multipodes*, *O. omeyrus*, *O. soratanus*, and *O. tiputini*.

Oroperipatus globulatus sp. nov.

Etymology: The specific epithet *globulatus* is derived from the Latin noun *globulus* (diminutive of *globus*), meaning “small ball”, combined with the adjectival suffix *atus*, meaning “provided with” or “having the nature of”. It refers to the characteristic globular shape of the apical pieces of the dorsal primary papillae. The epithet is formed as a Latin adjective in the nominative singular masculine and agrees in gender with the generic name *Oroperipatus*, in accordance with the International Code of Zoological Nomenclature (Articles 30.1.4, 31.2).

Coloration: Animals *in vivo* dorsally dark brown, dark dorsal midline, ventrally pale orange-brown. Preserved specimens without pattern, dorsally uniform brown, dark dorsal midline, ventrally beige; cephalic white collar absent.

Measurements: Body size in 70% ethanol: length 393–402 mm, width 38–40 mm, height 30–31 mm.

Antennae: Thirty-five complete antennal rings including terminal button. Antennal body with 22 rings; antennal tip with 12 rings + terminal button; 8th, 10th, 12th rings of tip thinner, lacking chemoreceptors. Chemoreceptors round, surrounded by four scales (Figure S22C); inner-most scales with needle-like projections. Type I antennal sensillae in all rings, with conical, two-ranked apical piece and blunt-ended bristle with structured base (Figure S23C). Type II sensillae restricted to antennal tip, with conical structured base (Figure S24C). Antennal sensory field on 22–24 proximal-most, complete rings and five interpolated incomplete rings. Spindle-shaped sensillae conical to oval, with 2–3 ranks of long, textured scales; terminal bristle blunt-ended (Figure S25C). Frontal organs elongated, ventrolateral at antennal base (Figure S26C).

Head: One pair of slime papillae; smaller median lobes lacking dermal papillae; larger lateral lobes with 1–3 lateral dermal papillae each (Figure S27C). Eyes pigmented, well-developed, ~80 µm in diameter (Figure S28C). Mouth with two lip rows: internal row with seven pairs of large lips + one unpaired anterior lip; external row with ca. nine pairs of small, variable lips (Figure S29C). Head pattern irregular.

Dorsal integument: Twelve complete plicae per segment; seven extend ventrally between leg pairs. Dorsomedian furrow distinct throughout body. Hyaline organs paired, inconspicuous. Alternate pattern of thin and thick plicae dorsally: thick plicae with large primary papillae + accessory papillae followed by thin plicae with small primary papillae + accessory papillae (Figure S30C). Dorsal primary papillae variable in size, asymmetrical roundish, with 2–3 scale ranks (Figure S30C); apical piece separated from basal by constriction (Figure S13C). Sensory bristle on apical piece thorn-shaped, size variable.

Two to four accessory papillae between primary papillae. Peripheral accessory papillae, few, small, flat (Figure S30C).

Ventral integument: Type I crater-shaped papillae oval, of three types: one with a rudimentary apical piece bearing a sensory bristle (Figure S31C), second with a rudimentary apical piece lacking a sensory bristle (Figure S31M) and third type without a rudimentary apical piece; apical collar with 8–9 scales (Figure S31C,M). Type II crater-shaped papillae oval, similar in size, without rudimentary apical piece; arranged in ventral and ventro-lateral rows of six papillae between adjacent legs; apical collar with 6–8 scales (Figure S32C). Preventral and ventral organs between leg pairs along ventral midline (except genital segment), separated by one row of papillae. Genital pad between penultimate leg pair, covered with scales; female genital opening cruciform (Figure S33C). Male sexual structures unknown. Terminal anus.

Leg: Examined females with 24 leg pairs (Table S1). Last pair reduced, rotated posteriorly; first and penultimate pairs similar in size, larger than last, smaller than others. Four spinous pads on developed legs (Figure S34C); three on penultimate, two on last pair. Spinous pads nearly straight, increasing proximally, covering about half of ventral leg surface; fourth pad thinner, smaller, about 2/3 of third pad length. Spines needle-shaped, with conical, textured base about 1/3 of spine length (Figure S34C with inset). Five transverse leg rings, separated by thinner, incomplete rings (Figure S19C). Bean-shaped papillae on dorsodistal leg portion, reduced in size, in pouch of two tegumental folds (Figure S19C). Nephridial tubercle on fourth and fifth leg pairs, inserted on third spinous pad, dividing it into small anterior, large posterior parts; tubercle attached to larger part, not indenting adjacent pads (Figure S11C). Eversible coxal vesicles on all legs except 4th, 5th, penultimate and last pairs. Four distal foot papillae per foot, two anterior, two posterior; papillae cylindrical, with globular to oval apical piece and short blunt-ended sensory bristle (Figure S12C). Proximal and distal setiform ridges with 1–2 bristles (Figure S35C).

Remarks: This species was collected at the edge of a paramo, at an altitudinal range of 3000–4000 m, specimens were widespread and found only in open areas. Thirty-seven specimens were collected and 24 were subsequently born in captivity, the number of leg pairs ranged from 22–25, specimens with 22–23 leg pairs were considered all males and those with 24–25 females. The species is considered small, females weighted 0.10–0.24 g and males >0.06 g.

Differential diagnosis: *Oroperipatus globulatus* **sp. nov.** can be distinguished from the *Oroperipatus* species examined in the present study by the unique combination of characters outlined in the diagnostic section. It can additionally be distinguished from previously described species of *Oroperipatus* as follows. The 12 complete dorsal plicae per segment differentiates it from *O. bimbergi*, *O. cameranoi, O. intermedius*, *O. koepckei*, *O. lankesteri*, *O. soratanus*, *O. tiputini*, *O. tuberculatus*, and *O. weyrauchi.* The number and arrangement of distal foot papillae, consisting of two anterior and two posterior papillae, distinguish it from *O. bluntschlii*, *O. ecuadorensis*, and *O. lankesteri*. Females of *O. globulatus* **sp. nov.** possess the lowest numbers of leg pairs recorded within *Oroperipatus* (24–25), distinguishing the species from nearly all congeners except *O. omeyrus*, which exhibits overlapping counts. In addition, the occurrence of only four complete spinous pads distinguishes this species from *O. bimbergi*, *O. bluntschlii*, *O. ecuadorensis*, *O. lankesteri*, *O. tiputini*, *O. tuberculatus*, and *O.*

weyrauchi. Finally, the dorsal primary papillae of *O. globulatus* **sp. nov.** possess apical pieces that are unusually larger than the basal pieces, a condition otherwise known only in *O. omeyrus* within the genus. Nevertheless, conspecificity between *O. globulatus* **sp. nov.** and *O. omeyrus* can be excluded based on their marked geographic separation, as the two species were recorded approximately 1,635 km apart.

Oroperipatus megapiculatus sp. nov.

Etymology: The specific epithet *megapiculatus* is formed from the Greek prefix *mega* ("large") combined with the Latin noun *apiculus* (diminutive of *apex*, meaning "apical point") and the adjectival suffix *atus* ("provided with" or "having the nature of"). The name refers to the prominent apical piece of the dorsal primary papillae. The epithet is a masculine Latin adjective in the nominative singular and agrees in gender with the generic name *Oroperipatus*, in accordance with the International Code of Zoological Nomenclature (Articles 30.1.4, 31.2).

Coloration: Preserved specimens without pattern, dorsally uniform dark brown, ventrally greyish brown with some tonalities of pinkish; cephalic white collar absent.

Measurements: Body size in 100% ethanol: length 297 mm, width 55 mm, height 26 mm.

Antennae: Forty-four complete antennal rings including terminal button. Antennal body with 32 rings; antennal tip with 11 rings + terminal button; 8th, 10th, and 12th rings of tip thinner, lacking chemoreceptors. Chemoreceptors round, surrounded by four scales (Figure S22D). Type I antennal sensillae in all antennal rings, with semi-elliptical, two-ranked apical piece and short needle-shaped sensory bristle with structured base (Figure S23D). Type II antennal sensillae restricted to antennal tip, with cylindrical to conical structured base (Figure S24D). Antennal sensory field on 31 proximal-most, complete rings and five interpolated incomplete rings. Spindle-shaped sensillae semi-elliptical to rod-like, with 2–3 ranks of long, textured scales; terminal bristle needle-shaped or blunt-ended (Figure S25D). Frontal organs elongated, ventrolateral at antennal base (Figure S26D).

Head: One pair of slime papillae; smaller median lobes lacking dermal papillae; larger lateral lobes with 1–2 large lateral dermal papillae each (Figure S27D). Eyes pigmented, well-developed, ~100 μm in diameter (Figure S28D). Mouth with two lip rows: internal row with six pairs of large lips + one unpaired anterior lip; external row with 11 pairs of small, variable lips (Figure S29D).

Dorsal integument: Ten complete and two incomplete plicae per segment; incomplete plicae at leg level, separated by complete plica; seven plicae extend ventrally between leg pairs. Dorsomedian furrow distinct throughout body. Hyaline organs inconspicuous. Primary and accessory papillae distributed irregularly along dorsal midline (Figure S30D). Dorsal primary papillae vary in size; basal piece conical, with 3–5 scale ranks (Figure S14D); apical piece separated from basal by constriction (Figures S13D and S14D). Sensory bristle thorn- or needle-shaped, size variable. One to four accessory papillae between primary papillae. Peripheral accessory papillae, small, conical (Figure S30D).

Ventral integument: Type I crater-shaped papillae oval, with rudimentary apical piece without sensory bristle; apical collar with 6–7 scales (Figure S31D). Type II crater-shaped papillae elongated, without rudimentary apical piece; arranged in ventral and ventro-lateral rows of six papillae between adjacent legs; apical collar with 7–11 scales (Figure S32D). One pair of interpedal structures per segment.

Preventral and ventral organs between leg pairs along ventral midline (except genital segment), positioned side by side, preventral organ collapsed in imaged segments. Genital pad between penultimate leg pair, cruciform, covered with primary and accessory papillae (Figure S33D). Male sexual structures unknown. Terminal anus.

Leg: Examined female with 27 leg pairs (Table S1). Last leg pair reduced, rotated posteriorly; first and penultimate pairs similar in size, larger than last, smaller than others. Four spinous pads on developed legs (Figure S34D); three on penultimate, two on last pair. Spinous pads nearly straight, increasing proximally, covering about half of ventral leg surface; fourth pad thinner, as large as third pad. Spines needle-shaped, with conical, textured base about 1/3 of spine length (Figure S34D with inset). Six transverse leg rings, separated by thinner, incomplete rings (Figure S19D). Bean-shaped papillae on dorsodistal leg portion, in pouch of two tegumental folds. Nephridial tubercle on fourth and fifth leg pairs, inserted on third spinous pad (deformed), dividing it into small anterior, large posterior parts (Figure S11D). Eversible coxal vesicles on all legs except 4th, 5th, penultimate and last pairs. Four distal foot papillae per foot, two anterior, two posterior (Figure S12D). Proximal and distal setiform ridges with two bristles (Figure S35D).

Differential diagnosis: *Oroperipatus megapiculatus* **sp. nov.** can be distinguished from the *Oroperipatus* species examined in the present study by the unique combination of characters outlined in the diagnostic section. It can additionally be distinguished from previously described species of *Oroperipatus* as follows. The presence of 10 complete and two incomplete dorsal plicae per segment differentiates it from *O. balzani*, *O. bluntschlii*, *O. corradi*, *O. ecuadorensis*, *O. multipodes*, *O. omeyrus*, and *O. tuberculatus*. The number and arrangement of distal foot papillae, consisting of two anterior and two posterior papillae, distinguish it from *O. bluntschlii*, *O. ecuadorensis*, and *O. lankesteri*. Females of *O. megapiculatus* **sp. nov.** possess more leg pairs than females of *O. omeyrus* but fewer leg pairs than those of most remaining congeners, with the exception of *O. bimbergi* and *O. corradi*, which exhibits overlapping leg-pair counts. Finally, the presence of only four complete spinous pads distinguishes *O. megapiculatus* **sp. nov.** from *O. bimbergi*, *O. bluntschlii*, *O. ecuadorensis*, *O. lankesteri*, *O. tiputini*, *O. tuberculatus*, and *O. weyrauchi*.

Oroperipatus onorei sp. nov.

Etymology: The specific epithet *onorei* is a Latinized genitive singular patronym derived from the modern personal name Onore, formed in accordance with Article 31.1.2 of the International Code of Zoological Nomenclature, and correctly combined with the masculine generic name *Oroperipatus*. The species honors Giovanni Onore, an Italian entomologist based in Ecuador and founder of the Quito Catholic Zoology Museum (QCZM), who collected the examined specimens. The patronym is intentionally formed as *onorei* rather than *onoreii*.

Coloration: Preserved specimens without dorsal pattern; dorsally reddish brown, ventrally beige to pinkish beige; cephalic white-collar present.

Measurements: Body size in 100% ethanol: length 296–455 mm, width 33–67 mm, height 25–34 mm.

Antennae: Thirty-three to thirty-five complete antennal rings including terminal button. Antennal body with 16–18 rings; antennal tip with 16 rings + terminal button; 9th and 11th rings of tip thinner, lacking

chemoreceptors. Chemoreceptors round, surrounded by 4–6 scales (Figure S22E). Type I antennal sensillae in all rings, with semi-oval or semi-elliptical, single-ranked apical piece and blunt-ended bristle with structured base (Figure S23E). Type II antennal sensillae restricted to antennal tip, with cylindrical, structured base (Figure S24E). Antennal sensory field on 10 proximal-most, complete rings, and five interpolated incomplete rings. Spindle-shaped sensillae oval, with 2–3 ranks of long, textured scales; terminal bristle blunt-ended (Figure S25E). Frontal organs elongated, ventrolateral at antennal base (Figure S26E).

Head: One pair of slime papillae; smaller median lobes lacking dermal papillae; larger lateral lobes with three dermal papillae each (Figure S27E). Eyes pigmented, well-developed, ~80 µm in diameter (Figure S28E). Mouth with two lip rows: internal row with five pairs of large lips + one unpaired anterior lip; external row with 13 pairs of small, variable lips (Figure S29E). Head pattern irregular.

Dorsal integument: Twelve complete plicae per segment; seven extend ventrally between leg pairs. Dorsomedian furrow distinct throughout body. Hyaline organs paired, inconspicuous. Primary and accessory papillae distributed irregularly along dorsal midline (Figure S30E). Dorsal primary papillae symmetrical, uniform in shape and size (Figure S30E). Basal piece round, with four scale ranks (Figure S14E); apical and basal pieces separated by inconspicuous constriction (Figure S13E). Apical piece with thorn-shaped sensory bristle variable in size (Figures S13E and S14E). Accessory papillae variable in size, roundish. One or two accessory papillae between two primary ones. Peripheral papillae, small, rosette-like (Figure S30E).

Ventral integument: Type I crater-shaped papillae round, with a rudimentary apical piece or lacking a rudimentary apical piece (Figure S31E,N); rudimentary apical piece with or without a sensory bristle; apical collar with 8–12 scales. Type II crater-shaped papillae larger, roundish, without rudimentary apical piece; arranged in ventral and ventro-lateral rows of six papillae between adjacent legs; apical collar with 12–15 scales (Figure S32E). Preventral and ventral organs between leg pairs along ventral midline (except genital segment), separated by three papillae rows. Genital pad between penultimate leg pair, densely covered with pointy scales (Figure S33E). Male sexual structures unknown. Terminal anus.

Leg: Examined females with 27–28 leg pairs (Table S1). Last leg pair reduced, rotated posteriorly. First and penultimate leg pairs similar in size, larger than last but smaller than remaining legs. Four spinous pads on developed legs (Figure S34E); three on first, two on last leg pair. Spinous pads arched, increasing in size proximally, covering about half of ventral leg surface; fourth pad thinner, smaller than third pad. Spines needle-shaped, with conical, textured base forming about 1/2 of spine length (Figure S34E with inset). Six transverse rings per leg, separated by thinner, incomplete rings (Figure S19E). Bean-shaped papillae on dorsodistal leg portion, reduced in size, inserted in a pouch formed by two tegumental folds. Nephridial tubercle on fourth and fifth leg pairs, inserted on third spinous pad, dividing it into small anterior and large posterior parts; tubercle attached to larger part (Figure S11E). Eversible coxal vesicles on all legs except 4th, 5th, penultimate and last pairs. Four distal foot papillae per foot, two anterior, two posterior; distal foot papillae cylindrical, with semi-oval apical piece and short, needle-ended sensory bristle (Figure S12E). Proximal setiform ridges with 2–3 bristles; distal setiform ridges with 1–2 bristles (Figure S35E).

Differential diagnosis: *Oroperipatus onorei* **sp. nov.** can be distinguished from the *Oroperipatus* species examined in the present study by the unique combination of characters outlined in the diagnostic section. It can additionally be distinguished from previously described species of *Oroperipatus* as follows. The 12 complete dorsal plicae per segment differentiates it from *O. bimbergi*, *O. cameranoi, O. intermedius*, *O. koepckei*, *O. lankesteri*, *O. soratanus*, *O. tiputini*, *O. tuberculatus*, and *O. weyrauchi.* The number and arrangement of distal foot papillae, consisting of two anterior and two posterior papillae, distinguish it from *O. bluntschlii*, *O. ecuadorensis*, and *O. lankesteri*. Females of *O. onorei* **sp. nov.** possess more leg pairs than females of *O. omeyrus* but fewer leg pairs than those of most remaining congeners, with the exception of *O. belli*, *O. bimbergi*, and *O. corradi*, which exhibits overlapping leg-pair counts. In addition, the presence of only four complete spinous pads distinguishes *O. megapiculatus* **sp. nov.** from *O. bimbergi*, *O. bluntschlii*, *O. ecuadorensis*, *O. lankesteri*, *O. tiputini*, *O. tuberculatus*, and *O. weyrauchi*. Finally, the presence of accessory papillae comparable in size to the primary ones distinguishes *O. onorei* **sp. nov.** from *O. belli* and *O. corradi*.

Oroperipatus palenquensis sp. nov.

Etymology: The specific epithet *palenquensis* is derived from the type locality along the banks of the Palenque River and is formed as a Latin adjectival toponym. The epithet is in the nominative singular and is correctly combined with the masculine generic name *Oroperipatus*, in accordance with Article 31.2 of the International Code of Zoological Nomenclature.

Coloration: Preserved specimens without dorsal pattern; dorsally pale or uniform dark brown, ventrally light beige; cephalic white collar absent.

Measurements: Body size in 100% ethanol: length 138–313 mm, width 18–46 mm, height 12–33 mm.

Antennae: Forty-five complete antennal rings including terminal button. Antennal body with 32 rings; antennal tip with 12 rings + terminal button; 7th, 9th, 11th rings of tip thinner, lacking chemoreceptors. Chemoreceptors round, surrounded by 5–6 scales (Figure S22F). Type I sensillae in all rings, with cylindrical to semi-oval, 1–2 ranked apical piece and blunt-ended bristle with structured base (Figure S23F). Type II sensillae restricted to antennal tip, with cylindrical, structured base (Figure S24F). Antennal sensory field on 28 proximal-most, complete rings and six interpolated incomplete rings; five distal-most rings of sensory field with single spindle-shaped sensilla. Spindle-shaped sensillae cylindrical to elliptical, with 2–3 ranks of long, textured scales; terminal bristle blunt-ended (Figure S25F). Frontal organs elongated, ventrolateral at antennal base (Figure S26F).

Head: One pair of slime papillae; smaller median lobes lacking dermal papillae; presence and number of dermal papillae on larger lateral lobes unclear (Figure S27F). Eyes pigmented, well-developed, ~150 µm in diameter (Figure S28F). Mouth with two lip rows: internal row with seven pairs of large lips + one unpaired anterior lip; external row with 10 pairs of small, variable lips (Figure S29F). Head pattern irregular.

Dorsal integument: Ten complete and two incomplete plicae per segment; incomplete plicae at leg level, separated by complete plica; seven plicae extend ventrally between leg pairs. Dorsomedian furrow distinct throughout body. Hyaline organs paired, round, with textured cuticle. Primary and accessory papillae in repeated pattern along dorsal midline: plica with one accessory papilla flanked by one

primary papilla on each side of dorsomedian furrow followed by plica with two accessory papillae flanked by one primary papilla (Figure S30F). Dorsal primary papillae uniform in shape/size; asymmetrical conical basal piece with 4–7 scale ranks (Figure S14F); apical and basal pieces separated by constriction. Sensory bristle on apical piece thorn-shaped, size variable (Figure S13F). One to four accessory papillae between primary papillae. Peripheral accessory papillae, rosette-like (Figure S30F).
Ventral integument: Type I crater-shaped papillae elongated to oval, with rudimentary apical piece; rudimentary apical piece with sensory bristle; apical collar with 7–8 scales (Figure S31F). Type II crater-shaped papillae oval, without rudimentary apical piece; apical collar of elongated papillae with 10–11 scales, five scales on small papillae (Figure S32F). One pair of interpedal structures per segment. Preventral and ventral organs between leg pairs along ventral midline (except genital segment), separated by 2–3 rows of papillae, preventral organ collapsed in imaged segments. Genital pad between penultimate leg pair, covered in scales (Figure S33F). Male sexual structures unknown. Terminal anus.
Leg: Examined females with 27–28 leg pairs (Table S1). Penultimate and last pairs reduced; last pair rotated posteriorly; penultimate pair larger than last but smaller than others. Four spinous pads on developed legs (Figure S34F), three on penultimate, two on last. Spinous pads nearly straight, covering about half of ventral leg surface; pads 1–3 similar in size; fourth pad smaller and thinner than third pad. Spines needle-shaped, with cylindrical, textured base about the same size as the spine length (Figure S34F with inset). Nine transverse rings per leg, separated by thinner, incomplete rings (Figure S19F). Bean-shaped papillae on dorsodistal leg portion, reduced in size, inserted in pouch formed by two tegumental folds; pouch closed in all imaged legs. Nephridial tubercle on fourth and fifth leg pairs, inserted on third spinous pad, dividing third spinous pad into small anterior and large posterior sections; tubercle attached to larger part, slightly indenting fourth pad (Figure S11F). Eversible coxal vesicles on all legs except 4th, 5th, penultimate and last pairs. Four distal foot papillae per foot, two anterior, two posterior (Figure S12F). Proximal setiform ridges with 1–2 bristles; distal ridges with one (Figure S35F).
Differential diagnosis: *Oroperipatus palenquensis* **sp. nov.** can be distinguished from the *Oroperipatus* species examined in the present study by the unique combination of characters outlined in the diagnostic section. It can additionally be distinguished from previously described species of *Oroperipatus* as follows. The presence of 10 complete and two incomplete dorsal plicae per segment differentiates it from *O. balzani*, *O. bluntschlii*, *O. corradi*, *O. ecuadorensis*, *O. multipodes*, *O. omeyrus*, and *O. tuberculatus*. The number and arrangement of distal foot papillae, consisting of two anterior and two posterior papillae, distinguish it from *O. bluntschlii*, *O. ecuadorensis*, and *O. lankesteri*. Females of *O. palenquensis* **sp. nov.** possess more leg pairs than females of *O. omeyrus* but fewer leg pairs than those of most remaining congeners, with the exception of *O. belli*, *O. bimbergi*, and *O. corradi*, which exhibits overlapping leg-pair counts. In addition, the presence of only four complete spinous pads distinguishes *O. palenquensis* **sp. nov.** from *O. bimbergi*, *O. bluntschlii*, *O. ecuadorensis*, *O. lankesteri*, *O. tiputini*, *O. tuberculatus*, and *O. weyrauchi*. Finally, the conical apical piece of dorsal primary papillae differs *O. palenquensis* **sp. nov.** from *O. belli*.

Oroperipatus sachaensis sp. nov.

Etymology: The specific epithet *sachaensis* is derived from the type locality, La Joya de los Sachas (commonly shortened to *Sacha*), a name of Quechua origin meaning "forest" and is formed as a Latin adjectival toponym. The epithet is in the nominative singular and is correctly combined with the masculine generic name *Oroperipatus*, in accordance with Article 31.2 of the International Code of Zoological Nomenclature.

Coloration: Preserved specimen without pattern, dorsally beige, ventrally light beige; cephalic white collar absent.

Measurements: Body size in 70% ethanol: length 580 mm, width 42 mm, height 35 mm.

Antennae: Forty-four complete antennal rings including terminal button. Antennal body with 32 rings; antennal tip with 12 rings + terminal button; 8th, 10th, and 12th rings of tip thinner, lacking chemoreceptors. Chemoreceptors round, surrounded by 4–6 scales (Figure S22G). Type I antennal sensillae in all antennal rings, with semi-oval, two-ranked apical piece and needle-shaped sensory bristle with structured base (Figure S23G). Type II antennal sensillae restricted to antennal tip, with cylindrical structured base (Figure S24G). Antennal sensory field on 31 proximal-most, complete rings and 16 interpolated incomplete rings. Spindle-shaped sensillae semi-elliptical to rod-like, with 2–3 ranks of long, textured scales; terminal bristle short, thorn to needle-shaped (Figure S25G). Frontal organs elongated, ventrolateral at antennal base (Figure S26G).

Head: One pair of slime papillae; smaller median lobes lacking dermal papillae; larger lateral lobes with 2–3 lateral dermal papillae each (Figure S27G). Eyes pigmented, well-developed, ~250 μm in diameter (Figure S28G). Mouth with two lip rows: internal row with seven pairs of large lips + one unpaired anterior lip, upper four lips bottle-like; external row with 13 pairs of small, variable lips (Figure S29G).

Dorsal integument: Ten complete and two incomplete plicae per segment; incomplete plicae at leg level, separated by complete plica; seven plicae extend ventrally between leg pairs. Dorsomedian furrow distinct throughout body. Hyaline organs paired, round, inconspicuous. Alternate pattern of plicae with largest primary papillae + accessory papillae and plicae with mid-sized/small primary papillae + accessory papillae (Figure S30G). Dorsal primary papillae vary in size; basal piece conical, asymmetrical, with 4–10 scale ranks (Figure S14G); apical piece separated from basal by constriction (Figures S13G and S14G). Sensory bristle short, thorn-shaped, variable in size; two sensory bristles on some apical pieces (Figure S14G). Accessory papillae similar in size, roundish to squarish (Figure S15G). One to four accessory papillae between primary papillae. Peripheral accessory papillae, few, small, rosette-like (Figure S30G).

Ventral integument: Type I crater-shaped papillae oval, with (Figure S31G) or without rudimentary apical piece lacking sensory bristle (Figure S31O); apical collar with 7–12 scales (Figure S31G,O). Type II crater-shaped papillae elongated, without vestigial apical piece; apical collar with 8–12 scales (Figure S32G). Preventral and ventral organs between leg pairs along ventral midline (except genital segment), separated by two rows of papillae. Genital pad between penultimate leg pair, densely covered with pointy scales; female genital opening cruciform (Figure S33G). Male sexual structures unknown. Terminal anus.

Leg: Examined females with 37 leg pairs (Table S1). Last leg pair reduced, rotated posteriorly; first and penultimate pairs similar in size, larger than last, smaller than others. Five spinous pads on developed

legs (Figure S34G); four on penultimate and last pairs. Spinous pads arched, increasing proximally in size, covering about half of ventral leg surface; fifth spinous pad thinner, smaller, about 2/3 of fourth pad length. Spines needle-shaped, with conical, textured base similar in size to the spine length (Figure S34G with inset). Six transverse leg rings, separated by thinner, incomplete rings (Figure S19G). Nephridial tubercle on fourth and fifth leg pairs, inserted on third spinous pad, dividing it into small anterior and large posterior parts; tubercle attached to larger part (Figure S11G). Eversible coxal vesicles on all legs except 4th and 5th pairs. Four distal foot papillae per foot, two anterior, two posterior; distal foot papillae cylindrical, with semi-oval to rod-like apical pieces and a short, blunt-ended sensory bristle (Figure S12G). Proximal and distal setiform ridges with 1–2 bristles (Figure S35G).

Differential diagnosis: *Oroperipatus sachaensis* **sp. nov.** can be distinguished from the *Oroperipatus* species examined in the present study by the unique combination of characters outlined in the diagnostic section. It can additionally be distinguished from previously described species of *Oroperipatus* as follows. The presence of 10 complete and two incomplete dorsal plicae per segment differentiates it from *O. balzani*, *O. bluntschlii*, *O. corradi*, *O. ecuadorensis*, *O. multipodes*, *O. omeyrus*, and *O. tuberculatus*. The number and arrangement of distal foot papillae, consisting of two anterior and two posterior papillae, distinguish it from *O. bluntschlii*, *O. ecuadorensis*, and *O. lankesteri*. Females of *O. sachaensis* **sp. nov.** possess fewer leg pairs than females of *O. bluntschlii*, *O. ecuadorensis* and *O. weyrauchi,* but more leg pairs than those of most remaining congeners, with the exception of *O. lankesteri*, *O. tiputini* and *O. tuberculatus*, which exhibits overlapping leg-pair counts. Finally, the presence of five complete spinous pads distinguishes *O. sachaensis* **sp. nov.** from *O. balzani*, *O. belli*, *O. cameranoi*, *O. corradi*, *O. intermedius*, *O. koepckei*, *O. multipodes*, *O. omeyrus*, *O. soratanus*, and *O. tiputini*.

Oroperipatus similaris sp. nov.

Etymology: The specific epithet *similaris* is derived from the Latin adjective *similaris*, meaning "similar to" or "resembling," and refers to the morphological similarity of this species to *Oroperipatus megapiculatus* **sp. nov.** The epithet is in the nominative singular and is correctly combined with the generic masculine name *Oroperipatus*, in accordance with Article 31.2 of the International Code of Zoological Nomenclature.

Coloration: Preserved specimen without pattern, dorsally uniform brown, ventrally greyish brown; cephalic white collar absent.

Measurements: Body size in 100% ethanol: length 284 mm, width 54 mm, height 36 mm.

Antennae: Forty-three complete antennal rings including terminal button. Antennal body with 30 rings; antennal tip with 11 rings + terminal button; 8th, 10th, and 12th rings of tip thinner, lacking chemoreceptors. Chemoreceptors round, surrounded by four scales (Figure S22H). Type I antennal sensillae in all antennal rings, with conical, 2–3 ranked apical piece and long needle-shaped sensory bristle with structured base (Figure S23H). Type II antennal sensillae restricted to antennal tip, with cylindrical structured base (Figure S24H). Antennal sensory field on 28 proximal-most, complete rings and six interpolated incomplete rings. Spindle-shaped sensillae semi-elliptical to rod-like, with two ranks

of long, textured scales; terminal bristle blunt-ended (Figure S25H). Frontal organs elongated, ventrolateral at antennal base (Figure S26H).

Head: One pair of slime papillae; smaller median lobes lacking dermal papillae; larger lateral lobes with two large lateral dermal papillae each (Figure S27H). Eyes pigmented, well-developed, ~150 μm in diameter (Figure S28H). Mouth with two lip rows: internal row with six pairs of large lips + one unpaired anterior lip; external row with 13 pairs of small, variable lips (Figure S29H).

Dorsal integument: Ten complete and two incomplete plicae per segment; incomplete plicae at leg level, separated by complete plica; seven plicae extend ventrally between leg pairs. Dorsomedian furrow distinct throughout body. Hyaline organs inconspicuous. Primary and accessory papillae distributed irregularly along dorsal midline (Figure S30H). Dorsal primary papillae varying in size, asymmetrical; basal piece conical, with 4–6 scale ranks (Figure S14H); apical piece separated from basal by constriction (Figures S13H and S14H). Sensory bristle thorn-shaped, size variable. Two to four accessory papillae between primary papillae. Peripheral accessory papillae, small, conical (Figure S30H).

Ventral integument: Type I crater-shaped papillae oval, with rudimentary apical piece bearing sensory bristle; apical collar with 6–7 scales (Figure S31H). Type II crater-shaped papillae elongated, without rudimentary apical piece; arranged in ventral and ventro-lateral rows of six papillae between adjacent legs; apical collar with 12 scales (Figure S32H). Three pairs of interpedal structures per segment. Preventral and ventral organs between each leg pair along ventral midline (except in genital segment), separated from each other by one row of papillae. Genital pad between penultimate leg pair, densely covered with pointy scales; female genital opening cruciform (Figure S33H). Male sexual structures unknown. Terminal anus.

Leg: Examined female with 27 leg pairs (Table S1). Last leg pair reduced, rotated posteriorly; first and penultimate leg pairs similar in size, larger than last, smaller than others. Four spinous pads on developed legs (Figure S34H); three on penultimate, two on last pair. Spinous pads arched, increasing proximally, covering about half of ventral leg surface; fourth pad thinner, smaller, about 2/3 of third pad length. Spines needle-shaped, with conical, textured base similar in size to the spine length (Figure S34H with inset). Six transverse leg rings, separated by thinner, incomplete rings (Figure S19H). Bean-shaped papillae on dorsodistal leg portion, with polygonal scales, in pouch of two tegumental folds (Figure S19H). Nephridial tubercle on fourth and fifth leg pairs, inserted on third spinous pad, dividing it or not into small anterior and large posterior parts; if divided, tubercle attached to larger part, indenting fourth pad (Figure S11H). Eversible coxal vesicles on all legs except 4th, 5th, penultimate and last pairs. Proximal setiform ridges and distal with two bristles (Figure S35H).

Differential diagnosis: *Oroperipatus similaris* **sp. nov.** can be distinguished from the *Oroperipatus* species examined in the present study by the unique combination of characters outlined in the diagnostic section. It can additionally be distinguished from previously described species of *Oroperipatus* as follows. The presence of 10 complete and two incomplete dorsal plicae per segment differentiates it from *O. balzani*, *O. bluntschlii*, *O. corradi*, *O. ecuadorensis*, *O. multipodes*, *O. omeyrus*, and *O. tuberculatus*. The number and arrangement of distal foot papillae, consisting of two anterior, two posterior and one dorsal papilla, distinguish it from nearly all congeners, with the exception of *O.*

bluntschlii, *O. ecuadorensis*, and *O. lankesteri*. Females of *O. similaris* **sp. nov.** possess more leg pairs than females of *O. omeyrus*, but less leg pairs than those of the remaining congeners. Finally, the presence of only four complete spinous pads distinguishes *O. similaris* **sp. nov.** from *O. bimbergi*, *O. bluntschlii*, *O. ecuadorensis*, *O. lankesteri*, *O. tiputini*, *O. tuberculatus*, and *O. weyrauchi*.

Oroperipatus specialis sp. nov.

Etymology: The specific epithet *specialis* is derived from the Latin adjective *specialis*, meaning "special" or "particular," and refers to the modified bean-shaped papilla that, to date, is unique to this species. The epithet is in the nominative singular and is correctly combined with the generic masculine name *Oroperipatus* in accordance with Article 31.2 of the International Code of Zoological Nomenclature.

Coloration: Preserved specimens without pattern, dorsally beige, ventrally light beige; cephalic collar white, complete.

Measurements: Body size in 70% ethanol: length 123–236 mm, width 30–47 mm, height 20–27 mm.

Antennae: Forty-two complete antennal rings including terminal button. Antennal body with 29 rings; antennal tip with 11 rings + terminal button; 9th, 11th, and 13th rings of tip thinner, lacking chemoreceptors. Chemoreceptors round, surrounded by four scales (Figure S22I). Type I antennal sensillae in all rings, with semi-oval, single-ranked apical piece and blunt-ended sensory bristle with structured base (Figure S23I). Type II antennal sensilla restricted to antennal tip, with cylindrical structured base (Figure S24I). Antennal sensory field on 14–16 proximal-most, complete rings and six interpolated incomplete rings. Spindle-shaped sensillae oval, with two ranks of long, textured scales; terminal bristle blunt-ended (Figure S25I). Frontal organs elongated, ventrolaterally at antennal base; with long modified scales at internal surface (Figure S26I).

Head: One pair of slime papillae; smaller median lobes lacking dermal papillae; larger lateral lobes thin, with two lateral dermal papillae each (Figure S27I). Eyes pigmented, well-developed, ~60 µm in diameter (Figure S28I). Mouth with two lip rows: internal row with seven pairs of large lips + one unpaired anterior lip; external row with nine pairs of small, variable lips (Figure S29I). Head pattern irregular.

Dorsal integument: Twelve complete plicae per segment; seven extend ventrally between leg pairs. Dorsomedian furrow distinct throughout body. Hyaline organs inconspicuous. Primary and accessory papillae distributed irregularly along dorsal midline (Figure S30I). Dorsal primary papilla similar in size and shape (Figure S30I), base asymmetrical, with 2–4 scale ranks (Figure S14I); apical piece separated from basal by constriction (Figures S13I and S14I). Sensory bristle on apical piece thorn-shaped, size variable. Accessory papillae rosette-like (Figure S15I). One to three accessory papillae between primary papillae. Peripheral accessory papillae, rare (Figure S30I).

Ventral integument: Type I crater-shaped papillae oval, with rudimentary apical piece bearing sensory bristle (Figure S31I) or without rudimentary apical piece (Figure S31P); apical collar with 9–14 scales (Figure S31I,P). Type II crater-shaped papillae elongated to roundish, without rudimentary apical piece; arranged in ventral and ventro-lateral rows of six papillae between adjacent legs: apical collar with nine scales (Figure S32I). Preventral and ventral organs collapsed in all imaged segments. Genital pad between penultimate leg pair, covered with pointy scales; genital opening cruciform in males and

females (Figures S20E and S33I). Male anal gland openings paired, median, longitudinal, nearly straight (Figure S20F). Terminal anus.

Leg: Examined male with 24 leg pairs, females with 26 (Table S1). First and last pairs reduced; last pair rotated posteriorly; penultimate leg pair larger than last and smaller than remaining legs. Four spinous pads on developed legs (Figure S34I); three on penultimate, two on last pair. Spinous pads nearly straight, increasing proximally, covering about half of ventral leg surface; fourth pad thinner, as large as third pad. Spines needle-shaped, with cylindrical, textured base about half of spine length (Figure S34I with inset). Eight transverse rings on each leg, separated from each other by thinner, incomplete rings (Figure S19I). Nephridial tubercle on fourth and fifth leg pairs, inserted on third spinous pad, dividing it into small anterior, large posterior parts; tubercle attached to larger part, indenting the fourth spinous pad (Figure S11I). Eversible coxal vesicles on all legs except 4th, 5th, penultimate and last pairs. Distal foot papillae cylindrical, with cylindrical to oval apical piece and short, blunt-ended sensory bristle (Figure S12I). Proximal setiform ridges with two bristles; distal with 1–2 bristles (Figure S35I). Two prominent crural tubercles per leg on two pregenital segments in males (Figure S20G). Crural tubercles conical to cylindrical, with smooth-surfaced apices; opening slit-like (Figure S20H).

Differential diagnosis: *Oroperipatus specialis* **sp. nov.** can be distinguished from the *Oroperipatus* species examined in the present study by the unique combination of characters outlined in the diagnostic section. It can additionally be distinguished from previously described species of *Oroperipatus* as follows. The 12 complete dorsal plicae per segment differentiates it from *O. bimbergi*, *O. cameranoi, O. intermedius*, *O. koepckei*, *O. lankesteri*, *O. soratanus*, *O. tiputini*, *O. tuberculatus*, and *O. weyrauchi.* The number and arrangement of distal foot papillae, consisting of two anterior, two posterior and one dorsal papilla, distinguish it from nearly all congeners, with the exception of *O. bluntschlii*, *O. ecuadorensis*, and *O. lankesteri*. Females of *O. specialis* **sp. nov.** possess fewer leg pairs than those of most remaining congeners, with the exception of *O. corradi* and *O. omeyrus*, which exhibits overlapping leg-pair counts. The number of leg pairs in males of this species is lower than in most remaining congeners, for which males are known. Exceptions include *O. belli*, *O. bimbergi*, and *O. corradi*, the males of which also exhibits overlapping leg-pair counts. Finally, the presence of only four complete spinous pads distinguishes *O. specialis* **sp. nov.** from *O. bimbergi*, *O. bluntschlii*, *O. ecuadorensis*, *O. lankesteri*, *O. tiputini*, *O. tuberculatus*, and *O. weyrauchi*.

Oroperipatus vallartensis sp. nov.

Etymology: The specific epithet *vallartensis* is derived from the type locality, Puerto Vallarta, Jalisco State, Mexico, and is formed as a Latin adjectival toponym. The epithet is in the nominative singular and is correctly combined with the generic masculine name *Oroperipatus* in accordance with Article 31.2 of the International Code of Zoological Nomenclature.

Coloration: Preserved specimens without pattern, dorsally uniform dark brown to grey, ventrally pale dark brown to grey; cephalic white collar absent.

Measurements: Body size in 70% ethanol: length 251–580 mm, width 25–42 mm, height 21–35 mm.

Antennae: Thirty-nine complete antennal rings including terminal button. Antennal body with 26 rings; antennal tip with 11 rings + terminal button; 9th, 11th and 13th rings of tip thinner, lacking chemoreceptors.

Chemoreceptors round, surrounded by 4–5 scales (Figure S22J). Type I antennal sensillae in all rings, with semi-oval, two-ranked apical piece and blunt-ended sensory bristle with structured base (Figure S23J). Type II antennal sensillae restricted to antennal tip, with cylindrical structured base (Figure S24J). Antennal sensory field on 26 complete rings and five interpolated incomplete rings. Spindle-shaped sensillae oval to semi-elliptical, with 2–3 ranks of long, textured scales; terminal bristle variable, needle-shaped or blunt-ended (Figure S25J). Frontal organs elongated, ventrolateral at antennal base (Figure S26J).

Head: One pair of slime papillae; smaller median lobes lacking dermal papillae; larger lateral lobes with 3–4 lateral dermal papillae each (Figure S27J). Eyes pigmented, well-developed, ~100 µm in diameter (Figure S28J). Mouth with two lip rows: internal row with seven pairs of large lips + one unpaired anterior lip; external row with seven pairs of small, variable lips (Figure S29J). Head pattern irregular.

Dorsal integument: Twelve complete plicae per segment; seven plicae extend ventrally between leg pairs. Dorsomedian furrow distinct throughout body. Hyaline organs paired, round, contiguous medially. Primary and accessory papillae distributed irregularly along dorsal midline (Figure S30J). Dorsal primary papillae similar in size and shape; basal piece round, with two scale ranks (Figure S14J); apical piece separated from basal by inconspicuous constriction (Figure S13J). Sensory bristle needle-shaped, variable in size. One to five accessory papillae between primary papillae. Peripheral accessory papillae, numerous, small, rosette-like, mainly on alternate plicae (Figure S30J).

Ventral integument: Type I crater-shaped papillae round, with rudimentary apical piece or with sensory bristle (Figure S31J); apical collar with 7–8 scales (Figure S31J). Type II crater-shaped papillae elongated, without rudimentary apical piece; arranged in ventral and ventro-lateral rows of six papillae between adjacent legs; apical collar with 9–11 scales (Figure S32J). Two pairs of interpedal structures per segment. Preventral and ventral organs between leg pairs along ventral midline (except genital segment), separated by one row of papillae. Genital pad between penultimate leg pair, female genital opening cruciform (Figure S33J). Male sexual structures unknown. Terminal anus.

Leg: Examined females with 27–28 leg pairs (Table S1). Last leg pair reduced, rotated posteriorly; first and penultimate pairs similar in size, larger than last, smaller than others. Four complete and a fifth vestigial spinous pads on developed legs (Figure S34J); three complete and one vestigial on penultimate and two on last pair. Spinous pads nearly straight, increasing proximally in size, covering about half of ventral leg surface; fourth spinous pad thinner, smaller, about 3/4 of third pad length. Spines needle-shaped, with conical, textured base about the same size of spine length (Figure S34J with inset). Six transverse leg rings, separated by thinner, incomplete rings (Figure S19J). Bean-shaped papillae on dorsodistal leg portion, in pouch of two tegumental folds. Nephridial tubercle on fourth and fifth leg pairs, inserted on third spinous pad, dividing it into small anterior, large posterior parts; tubercle attached to larger part, indenting third and fourth pad (Figure S11J). Eversible coxal vesicles on all legs except 4th, 5th, penultimate and last pairs. Four distal foot papillae per foot, two anterior, two posterior; distal foot papillae cylindrical, with semi-oval to semi-elliptical apical pieces and a short, blunt-ended or thorn-shaped sensory bristle (Figure S12J). Proximal setiform ridges with two bristles, distal setiform ridges with one bristle (Figure S35J).

Differential diagnosis: *Oroperipatus vallartensis* **sp. nov.** can be distinguished from the *Oroperipatus* species examined in the present study by the unique combination of characters outlined in the diagnostic section. It can additionally be distinguished from previously described species of *Oroperipatus* as follows. The 12 complete dorsal plicae per segment differentiates it from *O. bimbergi*, *O. cameranoi, O. intermedius*, *O. koepckei*, *O. lankesteri*, *O. soratanus*, *O. tiputini*, *O. tuberculatus*, and *O. weyrauchi.* The number and arrangement of distal foot papillae, consisting of two anterior and two posterior papillae, distinguish it from *O. bluntschlii*, *O. ecuadorensis*, and *O. lankesteri*. Females of *O. vallartensis* **sp. nov.** possess more leg pairs than females of *O. omeyrus*, but fewer leg pairs than those of most remaining congeners, with the exception of *O. belli*, *O. bimbergi*, and *O. corradi*, which exhibits overlapping leg-pair counts. In addition, the presence of only four complete spinous pads distinguishes *O. vallartensis* **sp. nov.** from *O. bimbergi*, *O. bluntschlii*, *O. ecuadorensis*, *O. lankesteri*, *O. tiputini*, *O. tuberculatus*, and *O. weyrauchi*. Finally, conspecificity between *O. vallartensis* **sp. nov.** and both *O. belli* and *O. corradi* can be excluded based on their marked geographic separation, as the latter two species were recorded in South America, respectively 3,767 km and 3,709 km apart from the type-locality of *O. vallartensis* **sp. nov.** in Mexico.

Oroperipatus variabilis sp. nov.

Etymology: The specific epithet *variabilis* is derived from the Latin adjective *variabilis*, meaning "variable" or "changeable," and refers to the morphological variability observed in the apical pieces of the dorsal primary papillae. The epithet is in the nominative singular and is correctly combined with the generic masculine name *Oroperipatus* in accordance with Article 31.2 of the International Code of Zoological Nomenclature.

Coloration: Preserved specimens without pattern, dorsally uniform dark brown, ventrally light beige; cephalic white collar absent.

Measurements: Body size in 70% ethanol: length 90–160 mm, width 13–30 mm, height 11–20 mm.

Antennae: Twenty-seven complete antennal rings including terminal button. Antennal body with 14 rings; antennal tip with 12 rings + terminal button; 8th, 10th, and 12th rings of tip thinner, lacking chemoreceptors. Chemoreceptors round, surrounded by five scales (Figure S22K). Type I antennal sensillae in all rings, with cylindrical to semi-elliptical, two-ranked apical piece and needle-shaped sensory bristle with structured base (Figure S23K). Type II antennal sensillae restricted to antennal tip and needle-shaped sensory bristle with a cylindrical structured base (Figure S24K). Antennal sensory field on 12 complete rings and 3–4 interpolated incomplete rings. Spindle-shaped sensillae semi-elliptical to rod-like, with two ranks of long, textured scales; terminal bristle needle-shaped or blunt-ended (Figure S25K). Frontal organs elongated, ventrolateral at antennal base (Figure S26K).

Head: One pair of slime papillae; smaller median lobes lacking dermal papillae; larger lateral lobes with two lateral dermal papillae each (Figure S27K). Eyes pigmented, well-developed, ~70 µm in diameter (Figure S28K). Mouth with two lip rows: internal row with seven pairs of large lips + one unpaired anterior lip; external row with five pairs of small, variable lips (Figure S29K). Head pattern irregular.

Dorsal integument: Twelve complete plicae per segment, seven of which pass between adjacent leg pairs to ventral side. Dorsomedian furrow distinct throughout body (Figure S30K). Hyaline organs

paired, variable in shape, contiguous medially. Basal piece of dorsal primary papillae round, with 2–3 scale ranks (Figures S14K and S21E–H); apical piece separated from basal by constriction (Figures S14K and S21E). Sensory bristle thorn- to needle-shaped, variable in size. Accessory papillae small, nearly flat, elliptical, apical-most scales with smooth tip (Figure S15K); One to four accessory papillae between primary papillae. Peripheral accessory papillae, absent.

Ventral integument: Type I and II crater-shaped papillae without vestigial apical piece. Type I crater-shaped papillae round to oval; apical collar with 4–8 scales (Figure S31K). Type II crater-shaped papillae elongated; arranged in ventral and ventro-lateral rows of six papillae between adjacent legs; apical collar with 5–12 scales (Figure S32K). Preventral and ventral organs between leg pairs along ventral midline (except genital segment), separated by one row of papillae. Interpedal structures irregular, with four scales with short villus-like, smooth projections (Figure S16K); two pairs of interpedal structures per segment. Genital pad between penultimate leg pair, covered with pointy scales. Male sexual structures unknown. Terminal anus.

Leg: Examined females with 26 leg pairs (Table S1). Last leg pair reduced, rotated posteriorly; first and penultimate pairs similar in size, larger than last, smaller than others. Four spinous pads on developed legs (Figure S34K); three on penultimate and two on last pair. Spinous pads arched, increasing proximally in size, covering about half of ventral leg surface; fourth spinous pad thinner, smaller, about 2/3 of third pad length. Spines needle-shaped, with conical, textured base about the same size of spine length (Figure S34K with inset). Five transverse leg rings, separated by thinner, incomplete rings (Figure S19K). Bean-shaped papillae on dorsodistal leg portion, prominent, in pouch of two tegumental folds (Figure S19K). Nephridial tubercle on fourth and fifth leg pairs, inserted on third spinous pad, dividing it into small anterior and large posterior parts; tubercle attached to larger part (Figure S11K). Eversible coxal vesicles on all legs except 4th, 5th, penultimate and last pairs. Four distal foot papillae per foot, two anterior, two posterior; distal foot papillae cylindrical, with semi-oval apical pieces and a short, needle-shaped or blunt-ended sensory bristle (Figure S12K). Proximal setiform ridges with two bristles, distal setiform ridges with one bristle (Figure S35K).

Differential diagnosis: *Oroperipatus variabilis* **sp. nov.** can be distinguished from the *Oroperipatus* species examined in the present study by the unique combination of characters outlined in the diagnostic section. It can additionally be distinguished from previously described species of *Oroperipatus* as follows. The 12 complete dorsal plicae per segment differentiates it from *O. bimbergi*, *O. cameranoi, O. intermedius*, *O. koepckei*, *O. lankesteri*, *O. soratanus*, *O. tiputini*, *O. tuberculatus*, and *O. weyrauchi.* The number and arrangement of distal foot papillae, consisting of two anterior and two posterior papillae, distinguish it from *O. bluntschlii*, *O. ecuadorensis*, and *O. lankesteri*. Females of *O. variabilis* **sp. nov.** possess fewer leg pairs than those of most remaining congeners, with the exception of *O. corradi* and *O. omeyrus*, which exhibits overlapping leg-pair counts. In addition, the presence of only four complete spinous pads distinguishes *O. variabilis* **sp. nov.** from *O. bimbergi*, *O. bluntschlii*, *O. ecuadorensis*, *O. lankesteri*, *O. peruvianus*, *O. tiputini*, *O. tuberculatus*, and *O. weyrauchi*. Finally, conspecificity of *Oroperipatus variabilis* **sp. nov.** with either *O. corradi* or *O. omeyrus* can be rejected based on their pronounced geographic disjunctions. *Oroperipatus corradi* was originally described from high-elevation habitats (~3000 m ASL), located more than 110 km from the type locality of *O. variabilis*

sp. nov. (372 m ASL), whereas *O. omeyrus* is distributed in Peru, approximately 1,625 km distant from the new species.

Oroperipatus yarapensis sp. nov.

Etymology: The specific epithet *yarapensis* is derived from the type locality along the banks of the Yarapa River, Department of Loreto, Peru, and is formed as a Latin adjectival toponym. The epithet is in the nominative singular and is correctly combined with the generic masculine name *Oroperipatus* in accordance with Article 31.2 of the International Code of Zoological Nomenclature.

Coloration: Preserved specimen without pattern, dorsally uniform light brown, ventrally pale; cephalic collar white, incomplete.

Measurements: Body size in 70% ethanol: length 409 mm, width 58 mm, height 33 mm.

Antennae: Forty-six complete antennal rings including terminal button. Antennal body with 33 rings; antennal tip with 12 rings + terminal button; 8th, 10th, 12th rings of tip thinner lacking chemoreceptors. Chemoreceptors round, surrounded by five scales (Figure S22L). Type I antennal sensillae in all rings, with conical to oval, three-ranked apical piece and blunt-ended or needle-shaped sensory bristle with structured base (Figure S23L). Type II antennal sensillae restricted antennal tip, with blunt-ended sensory bristle with a cylindrical to conical structured base (Figure S24L). Antennal sensory field on 22 complete rings and three interpolated incomplete rings. Spindle-shaped sensillae oval to rod-like, with 3–4 ranks of long, textured scales; terminal bristle needle-shaped (Figure S25L). Frontal organs elongated, ventrolateral at antennal base (Figure S26L).

Head: One pair of slime papillae; smaller median lobes lacking dermal papillae; larger lateral lobes with five lateral dermal papillae (Figure S27L). Eyes pigmented, well-developed, ~200 μm in diameter (Figure S28L). Mouth with two lip rows: internal row with seven pairs of large lips + one unpaired anterior lip; external row with nine pairs of small, variable lips (Figure S29L).

Dorsal integument: Ten complete and two incomplete plicae per segment; incomplete plicae at leg level, separated by complete plica; seven plicae extend ventrally between leg pairs. Dorsomedian furrow distinct throughout body. Hyaline organs inconspicuous. Alternate pattern of thin and thick plicae dorsally: thick plicae with largest and midsized primary papillae + accessory papillae followed by thin plicae with midsized and small primary papillae + accessory papillae (Figure S30L). Dorsal primary papillae variable in size; basal piece oval, with 8–12 scale ranks (Figure S14L); apical piece separated from basal by constriction (Figures S13L and S14L). Sensory bristle thorn- to needle-shaped, variable in size. One to four accessory papillae between primary papillae. Peripheral accessory papillae conical, few (Figure S30L).

Ventral integument: Type I crater-shaped papillae oval, without rudimentary apical piece; apical collar with of 10 scales (Figure S31L). Type II crater-shaped papillae elongated, without apical piece; arranged in ventral and ventro-lateral rows of six papillae between adjacent legs; apical collar with 9–13 scales (Figure S32L). Preventral and ventral organs between leg pairs along ventral midline (except genital segment), preventral organ collapsed in all imaged segments. Interpedal structures round, with six scales with short villus-like, smooth projections (Figure S16L); one pair of interpedal structures per segment. Genital pad between penultimate leg pair, with numerous tegumental folds covered with

scales and bearing sensory bristle; male genital opening cruciform (Figure S20I). Male anal gland openings paired, median, longitudinal, curved (Figure S20J). Terminal anus.

Leg: Examined male with 37 leg pairs (Table S1). Last leg pair reduced, rotated posteriorly; first and penultimate pairs similar in size, larger than last, smaller than others. Five spinous pads on developed legs (Figure S34L); four on first and penultimate pairs, three complete and one vestigial on last pair. Spinous pads nearly straight, increasing proximally in size, covering about half of ventral leg surface; fifth spinous pad smaller, about 3/4 of fourth pad length. Spines needle-shaped, with cylindrical, textured base about the same size of spine length (Figure S34L with inset). Four transverse leg rings, separated by thinner, incomplete rings (Figure S19L). Nephridial tubercle on fourth and fifth leg pairs, inserted on third spinous pad, dividing it into small anterior and large posterior parts; tubercle attached to larger part (Figure S11L). Eversible coxal vesicles on all legs except 4th and 5th pairs. Four distal foot papillae per foot, two anterior, two posterior; distal foot papillae cylindrical, with conical apical pieces and a short, needle-like sensory bristle (Figure S12L). Proximal and distal setiform ridges with 1–2 bristles (Figure S35L).

Differential diagnosis: *Oroperipatus yarapensis* **sp. nov.** can be distinguished from the *Oroperipatus* species examined in the present study by the unique combination of characters outlined in the diagnostic section. It can additionally be distinguished from previously described species of *Oroperipatus* as follows. The presence of 10 complete and two incomplete dorsal plicae per segment differentiates it from *O. balzani*, *O. bluntschlii*, *O. corradi*, *O. ecuadorensis*, *O. multipodes*, *O. omeyrus*, and *O. tuberculatus*. The number and arrangement of distal foot papillae, consisting of two anterior and two posterior papillae, distinguish it from *O. bluntschlii*, *O. ecuadorensis*, and *O. lankesteri*. Males of *Oroperipatus yarapensis* **sp. nov.** possess fewer leg pairs than those of *O. weyrauchi*, but more than those of most remaining congeners, for which males are known. The exception includes *O. tuberculatus*, the males of which exhibits overlapping leg-pair counts. Finally, the presence of five complete spinous pads distinguishes *O. yarapensis* **sp. nov.** from *O. balzani*, *O. belli*, *O. cameranoi*, *O. corradi*, *O. intermedius*, *O. koepckei*, *O. multipodes*, *O. omeyrus*, *O. soratanus*, and *O. tiputini*.

REMARKS ON MORPHOLOGICAL CHARACTERS AND SPECIES DELIMITATION

Prior to this study, knowledge of morphological diversity within *Oroperipatus* was very limited and primarily derived from early species descriptions [S4-7]. In addition to demonstrating that *Oroperipatus* species are morphologically more diverse than previously recognized, our data also revealed that features exhibiting limited diagnostic utility within the Caribbean clade of Neopatida emerge as consistent and reliable taxonomic characters in *Oroperipatus*. These include the structure of the apical pieces in the dorsal primary papillae, the number and morphology of distal foot papillae, and specific modifications in the apical-most scales of dorsal accessory papillae (see Oliveira et al., [S8,9]). Our findings further indicate that the number, morphology, and arrangement of interpedal structures, as well as characteristics of embryonic foot projections, may serve as informative characters for species delimitation within *Oroperipatus*. However, these characters have only been examined in a few non-*Oroperipatus* taxa [S10-13], highlighting the need for a broader comparative analysis across the clade to clarify their diagnostic value.

Many of the morphological characters identified in this study have not yet been surveyed comprehensively across *Oroperipatus*, which limits direct comparisons between our findings and previously published accounts. Where comparisons are possible, we detected interspecific differences in the total number and sex-specific variation of leg pairs, in the number and arrangement of distal foot papillae and spinous pads, and in features of the dorsal integument, including plicae organisation and the distribution of primary and accessory papillae. Given that interspecific variation within Peripatidae is generally subtle [S11-12], the suite of differences documented here provides sufficient evidence to support recognition of the new species described herein, as further reflected in the differential diagnoses of each species.

For taxa represented by molecular data, species distinctiveness is additionally supported by single-locus species delimitation analyses, although the results varied according to the genetic marker and delimitation method employed. Delimitation analyses based on *COI* and *16S rRNA* yielded results largely congruent with the phylogenetic and morphological evidence, recovering nearly all examined taxa as distinct species. The main exception involved the Mexican and Galápagos taxa, which were consistently recovered as a single species. Nevertheless, these taxa are morphologically distinct and were also recovered as independent evolutionary lineages in all phylogenetic analyses. In contrast, analyses based on *12S rRNA* tended to overestimate species diversity, frequently delimiting even conspecific specimens as separate species. It should be noted, however, that the performance of species delimitation analyses may have been constrained by the limited sampling available for most localities, which often consisted of only one or two individuals. Such restricted sampling can reduce the accuracy of species delimitation methods and may lead to both over- and underestimation of species boundaries [S14-15]. This limitation was mitigated by integrating additional lines of evidence, including morphological data and multilocus molecular phylogenetic analyses, which provided independent support for species boundaries and taxonomic decisions.

When considering only the *Oroperipatus* species examined here, four species exhibit particularly distinctive morphological traits. *Oroperipatus specialis* **sp. nov.** exhibits a highly modified, previously undocumented form of bean-shaped papillae, characterized by an eversible, stalked structure with a spherical apex, the function of which remains unknown. In contrast, *O. peruvianus*, *O. sachaensis* **sp. nov.**, and *O. yarapensis* **sp. nov.** share the absence of bean-shaped papillae on the legs and a dorsodistal leg region with absent or markedly reduced dermal papillae—features otherwise present in all neopatids. We additionally observed that embryos of *O. peruvianus* and *O. sachaensis* **sp. nov.** lack embryonic foot projections, whereas this character could not be assessed in *O. yarapensis* **sp. nov.** because only a single male specimen was available. Interestingly, a pattern like in these three species has been documented in representatives of *Eoperipatus* from Southeast Asia [S11-12], the sister group to Neopatida + *Mesoperipatus tholloni* [S16] from tropical Africa. However, this resemblance is more plausibly attributed to secondary loss within *Oroperipatus* rather than to the retention of a plesiomorphic condition, given that other examined congeners retain both bean-shaped and dorsodistal dermal papillae on the legs, as well as embryonic foot projections in the embryos.

Our molecular phylogenetic analyses, which incorporated samples from across the known geographic distribution of *Oroperipatus*, recovered two well-supported subclades: the Amazonian

subclade comprising eastern Andean and Amazonian taxa, and the Pacific subclade consisting of western Andean, Galápagos, and Mexican species. The newly described species are nested within clades consistent with their respective geographic ranges. Even though each of these two major clades shows a set potential molecular diagnostic characters in *18S rRNA* sequences, a preliminary assessment indicates that they do not display clear morphological autapomorphies. The absence of bean-shaped papillae and the reduction or loss of dermal papillae on the dorsal leg integument may represent potential synapomorphies of the Amazonian subclade, but this remains tentative and requires further investigation.

Although these two subclades of *Oroperipatus* have been consistently recovered in previous molecular studies [S17-19], their mutual relationships and their affinities with other neopatids have remained unstable across the literature. In particular, the Amazonian subclade has been recovered either as sister to the Pacific subclade—thereby supporting the monophyly of *Oroperipatus*—or as sister to the Caribbean clade, rendering *Oroperipatus* paraphyletic (see Giribet et al., [S17]). In the present study, *Oroperipatus* is consistently recovered as a well-supported monophyletic clade in all phylogenetic analyses employing neopatids and their immediate sister taxon (*Mesoperipatus tholloni*) as the outgroup. This monophyly is further corroborated by phenotypic evidence, as representatives of *Oroperipatus* are also morphologically highly distinct from other neopatids [S5].

By contrast, the inclusion of Southeast Asian Peripatidae (*Eoperipatus*) and representatives of Peripatopsidae in the outgroup resulted in a weakly supported, paraphyletic topology for *Oroperipatus*. These results suggest that phylogenetic artefacts—such as long-branch attraction associated with highly derived taxa like *Eoperipatus*, and/or the pronounced sequence divergence in the *18S rRNA* gene between peripatids and peripatopsids, which introduces extensive gaps in the final alignments—may obscure the phylogenetic signal and substantially affect inferred relationships within *Oroperipatus*. Consequently, we favoured a more restricted outgroup selection to infer the phylogenetic relationships of *Oroperipatus*.

The exclusive reliance on collection-based material imposed significant constraints on this study, highlighting the intrinsic challenges of employing historical specimens in systematic research on soft-bodied invertebrates such as onychophorans. While preservation artifacts frequently hindered the accurate assessment of key morphological features, molecular analyses were often limited by DNA degradation and low extraction yields. As a result, certain species, namely *Oroperipatus globulatus* **sp. nov.**, *O. onorei* **sp. nov.**, and *O. yarapensis* **sp. nov.**, could not be incorporated into the molecular dataset. Nevertheless, the recovery of reciprocal monophyly between eastern and western Andean *Oroperipatus* suggests a tentative placement of *O. yarapensis* **sp. nov.** within the Amazonian subclade, and *O. globulatus* **sp. nov.** and *O. onorei* **sp. nov.** within the Pacific subclade, based on their respective geographic distributions.

Similar limitations also affected the analysis of the type-species *Oroperipatus lankesteri*. Comparative morphological assessments were not feasible due to institutional restrictions on specimen handling, whereas only partial sequences of the *16S rRNA* gene were successfully amplified, owing to the degraded DNA quality of the single specimen available. Despite these constraints, this study provides the first molecular phylogenetic evidence supporting the placement of *O. lankesteri* within the

Pacific subclade of *Oroperipatus*. Although *16S rRNA* sequences can exhibit high levels of autapomorphy[17], this does not appear to be the case for *O. lankesteri*, the phylogenetic position of which is consistent with its geographic origin. This finding challenges an earlier hypothesis suggesting an eastern Andean origin for *O. lankesteri*, which was primarily based on the number of leg pairs [S17]. However, this incongruence is not unexpected, given that the number of leg pairs is known to vary intraspecifically across most peripatid species and therefore lacks sufficient evolutionary signal for inferring the phylogenetic placement of species. Unfortunately, examination of the type material of nominal species using the analytical approaches employed in this study was not possible, owing to the invasive nature of scanning electron microscopy (SEM) imaging and tissue sampling required for molecular analyses, together with restrictions imposed by the holding institutions on destructive or invasive examination of type specimens.

SUPPLEMENTAL REFERENCES

1. Oliveira, I.S. (2023). An updated world checklist of velvet worms (Onychophora) with notes on nomenclature and status of names. ZooKeys *1184*, 133–260. 10.3897/zookeys.1184.107286.
2. Wheeler, W.M. (1898). A new *Peripatus* from Mexico. Journal of Morphology *15*, 1–8. 10.1002/jmor.1050150102.
3. Brues, C.T. (1917). A new species of *Peripatus* from the mountains of northern Peru. Bulletin of the Museum of Comparative Zoology *61*, 383–387.
4. Camerano, L. (1898). Nouva specie di *Peripatus* dell'Ecuador. Atti R Accad Sci Torino *33*, 308–310.
5. Bouvier, E.L. (1905). Monographie des Onychophores. Ann Sci Nat Zool Biol Anim [9e Série] *2*, 1–383.
6. du Bois-Reymond Marcus, E. (1952). On South American Malacopoda. Boletins da Faculdade de Filosofia, Ciências e Letras da Universidade de São Paulo. Série Zoologia *17*, 189–209. 10.11606/issn.2526-4877.bsffclzoologia.1952.125190.
7. Zilch, A. (1954). Ein neuer *Oroperipatus* aus Peru. Senckenbergiana Biologica *35*, 151–154.
8. Oliveira, I.S., Wieloch, A.H., and Mayer, G. (2010). Revised taxonomy and redescription of two species of the Peripatidae (Onychophora) from Brazil: a step towards consistent terminology of morphological characters. Zootaxa *2493*, 16–34. 10.11646/zootaxa.2493.1.2.
9. Oliveira, I.S., Lacorte, G.A., Fonseca, C.G., Wieloch, A.H., and Mayer, G. (2011). Cryptic speciation in Brazilian *Epiperipatus* (Onychophora: Peripatidae) reveals an underestimated diversity among the peripatid velvet worms. PLOS ONE *6*, e19973. 10.1371/journal.pone.0019973.
10. Walker, M., and Campiglia, S. (1988). Some aspects of segment formation and post-placental development in *Peripatus acacioi* Marcus and Marcus (Onychophora). Journal of Morphology *195*, 123–140. 10.1002/jmor.1051950202.
11. Oliveira, I.S., Franke, F.A., Hering, L., Schaffer, S., Rowell, D.M., Weck-Heimann, A., Monge-Nájera, J., Morera-Brenes, B., and Mayer, G. (2012). Unexplored character diversity in Onychophora (velvet worms): a comparative study of three peripatid species. PLOS ONE *7*, e51220. 10.1371/journal.pone.0051220.
12. Oliveira, I.S., Schaffer, S., Kvartalnov, P.V., Galoyan, E.A., Palko, I.V., Weck-Heimann, A., Geissler, P., Ruhberg, H., and Mayer, G. (2013). A new species of *Eoperipatus* (Onychophora) from Vietnam reveals novel morphological characters for the South-East Asian Peripatidae. Zoologischer Anzeiger *252*, 495–510. 10.1016/j.jcz.2013.01.001.
13. Oliveira, I.S., Lacorte, G.A., Weck-Heimann, A., Cordeiro, L.M., Wieloch, A.H., and Mayer, G. (2015). A new and critically endangered species and genus of Onychophora (Peripatidae) from the Brazilian savannah—a vulnerable biodiversity hotspot. Systematics and Biodiversity *13*, 211–233. 10.1080/14772000.2014.985621.
14. Puillandre, N., Lambert, A., Brouillet, S., and Achaz, G. (2012). ABGD, Automatic Barcode Gap Discovery for primary species delimitation. Molecular ecology *21*, 1864-1877. 10.1111/j.
15. Roy, V., Constantino, R., Chassany, V., Giusti-Miller, S., Diouf, M., Mora, P., and Harry, M. (2014). Species delimitation and phylogeny in the genus Nasutitermes (Termitidae: Nasutitermitinae) in French Guiana. Molecular Ecology *23*, 902–920. 10.1111/mec.12641.

16. Bouvier, E.L. (1898). On the geographical distribution and the evolution of *Peripatus* (preliminary note). Annals and Magazine of Natural History [Series 7] *2*, 351–353.
17. Giribet, G., Buckman-Young, R.S., Costa, C.S., Baker, C.M., Benavides, L.R., Branstetter, M.G., Daniels, S.R., and Pinto-da-Rocha, R. (2018). The '*Peripatos*' in Eurogondwana? – Lack of evidence that south-east Asian onychophorans walked through Europe. Invertebrate Systematics *32*, 842–865. 10.1071/IS18007.
18. Sato, S., Derkarabetian, S., Lord, A., and Giribet, G. (2024). An ultraconserved element probe set for velvet worms (Onychophora). Molecular Phylogenetics and Evolution *197*, 108115. 10.1016/j.ympev.2024.108115.
19. Costa, C.S., and Giribet, G. (2021). Panamanian velvet worms in the genus *Epiperipatus*, with notes on their taxonomy and distribution and the description of a new species (Onychophora, Peripatidae). Invertebrate Biology *140*, e12336. 10.1111/ivb.12336.
20. Pérez-Escobar, O.A., Zizka, A., Bermúdez, M.A., Meseguer, A.S., Condamine, F.L., Hoorn, C., Hooghiemstra, H., Pu, Y., Bogarín, D., Boschman, L.M., et al. (2022). The Andes through time: evolution and distribution of Andean floras. Trends in Plant Science *27*, 364–378. 10.1016/j.tplants.2021.09.010.

SUPPLEMENTAL FIGURES

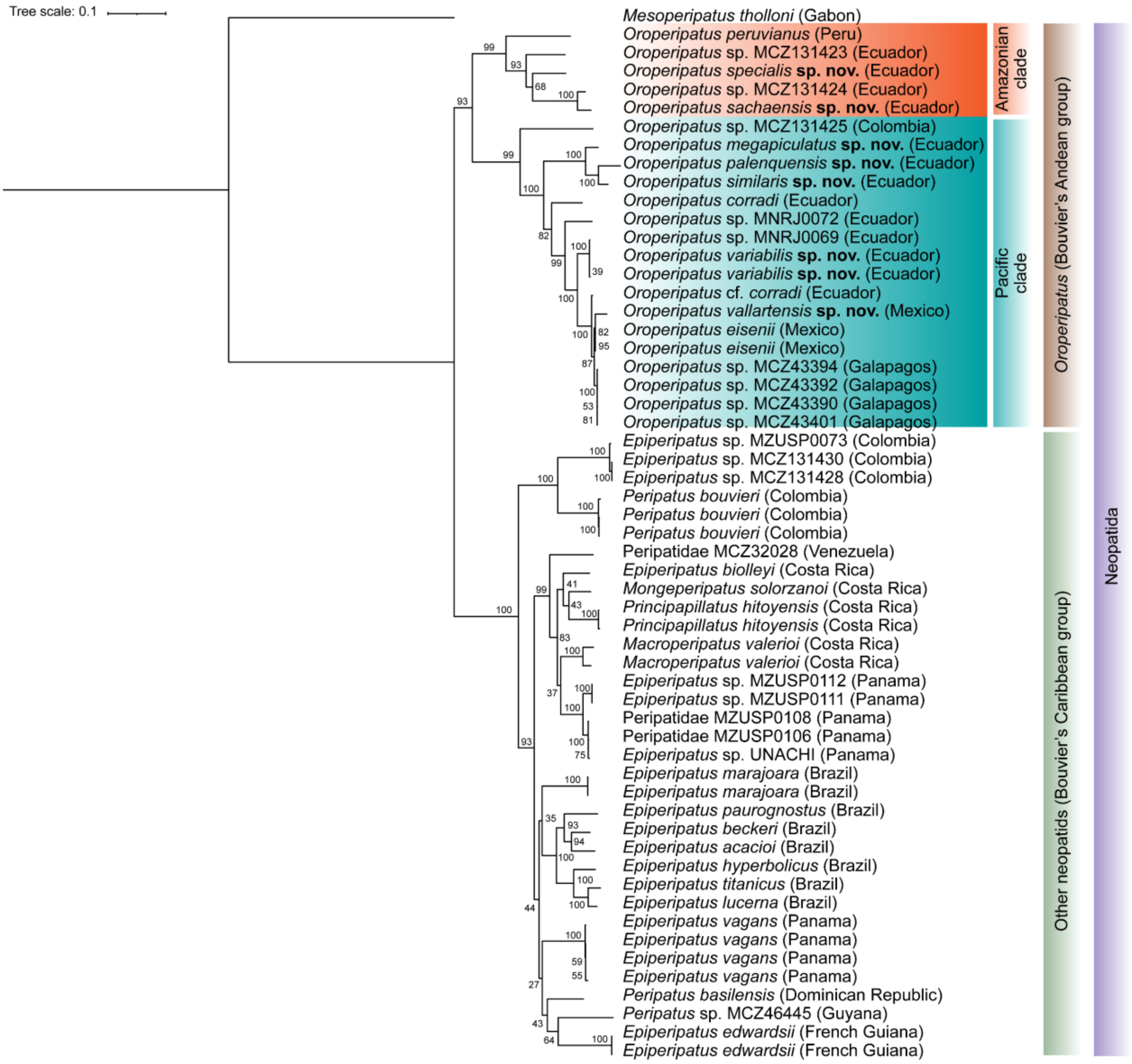

Figure S1. Phylogenetic relationships among studied *Oroperipatus* species, Related to Results. Maximum likelihood topology combining nucleotide sequences of the *12S rRNA*, *16S rRNA*, *18S rRNA* and *COI* genes. Outgroup excluding peripatopsids and *Eoperipatus*. The type species *O. lankesteri* is not included. Numbers indicate bootstrap support values. Note the well-supported Amazonian (orange) and Pacific (blue) subclades of *Oroperipatus*. See also Figure 2, and S2–S9.

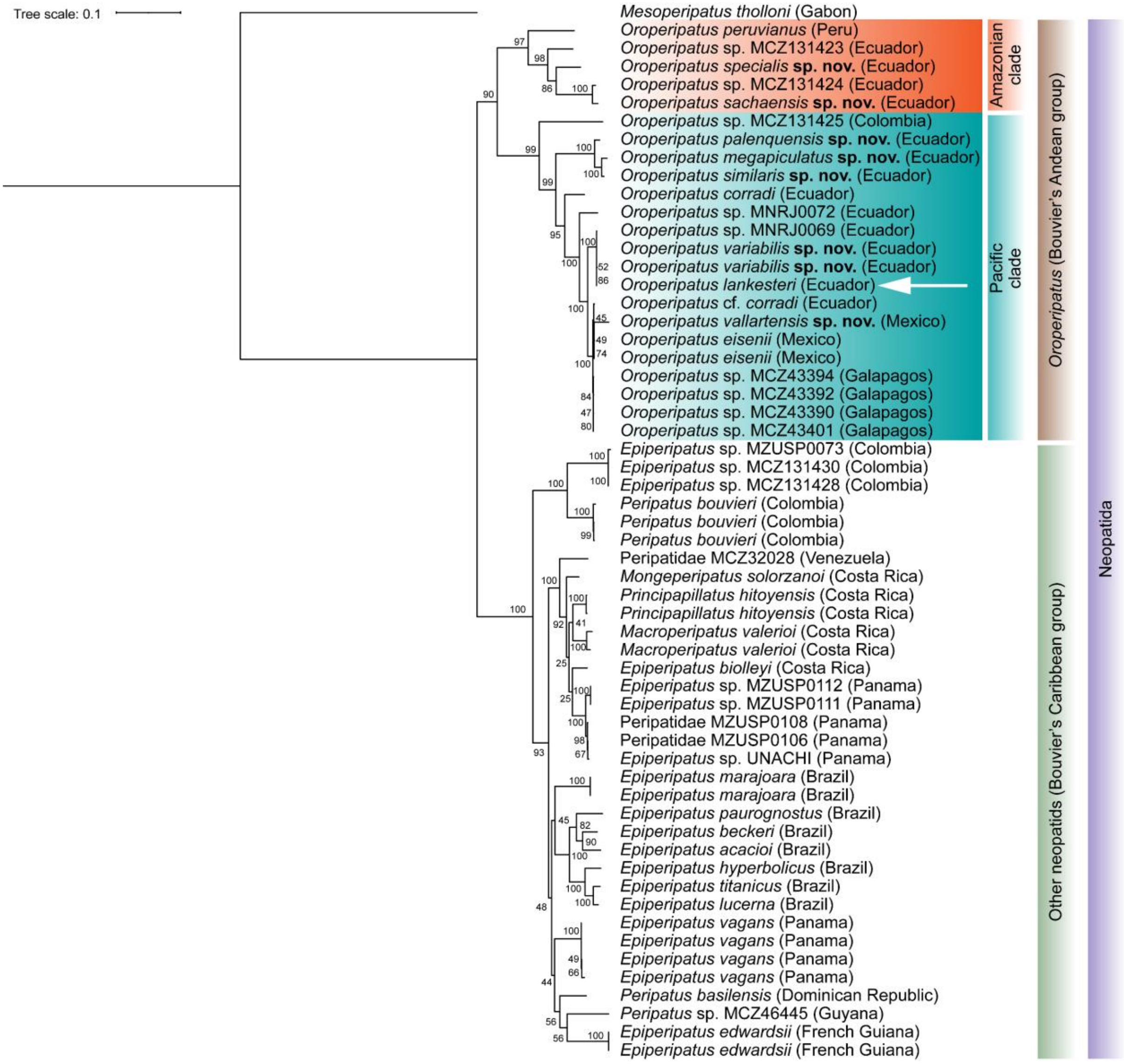

Figure S2. Phylogenetic relationships among studied *Oroperipatus* species, Related to Results. Maximum likelihood topology combining nucleotide sequences of the *12S rRNA*, *16S rRNA*, *18S rRNA* genes and *COI* gene sequences excluding the third codon positions. Outgroup excluding peripatopsids and *Eoperipatus*. Arrow indicates the position of the type species *O. lankesteri*. Numbers indicate bootstrap support values. Note the well-supported Amazonian (orange) and Pacific (blue) subclades of *Oroperipatus*. See also Figure 2, S1, and S3–S9.

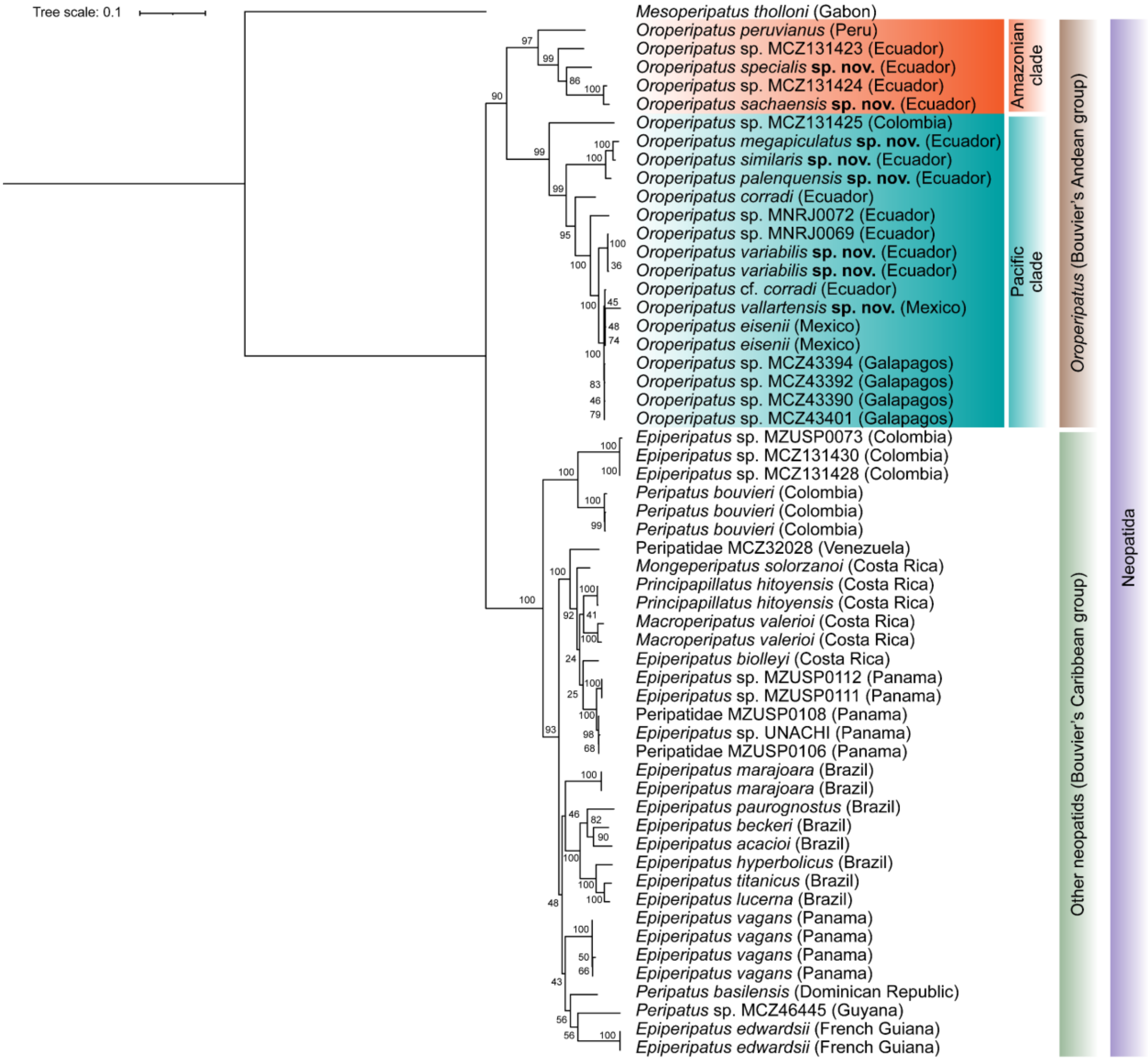

Figure S3. Phylogenetic tree showing the relationship of the studied species, Related to Results. Maximum likelihood topology combining nucleotide sequences of the *12S rRNA*, *16S rRNA*, *18S rRNA* genes and *COI* gene sequences excluding the third codon positions. Outgroup excluding peripatopsids and *Eoperipatus*. The type species *O. lankesteri* is not included. Numbers indicate bootstrap support values. Note the well-supported Amazonian (orange) and Pacific (blue) subclades of *Oroperipatus*. See also Figure 2, S1, S2, and S4–S9.

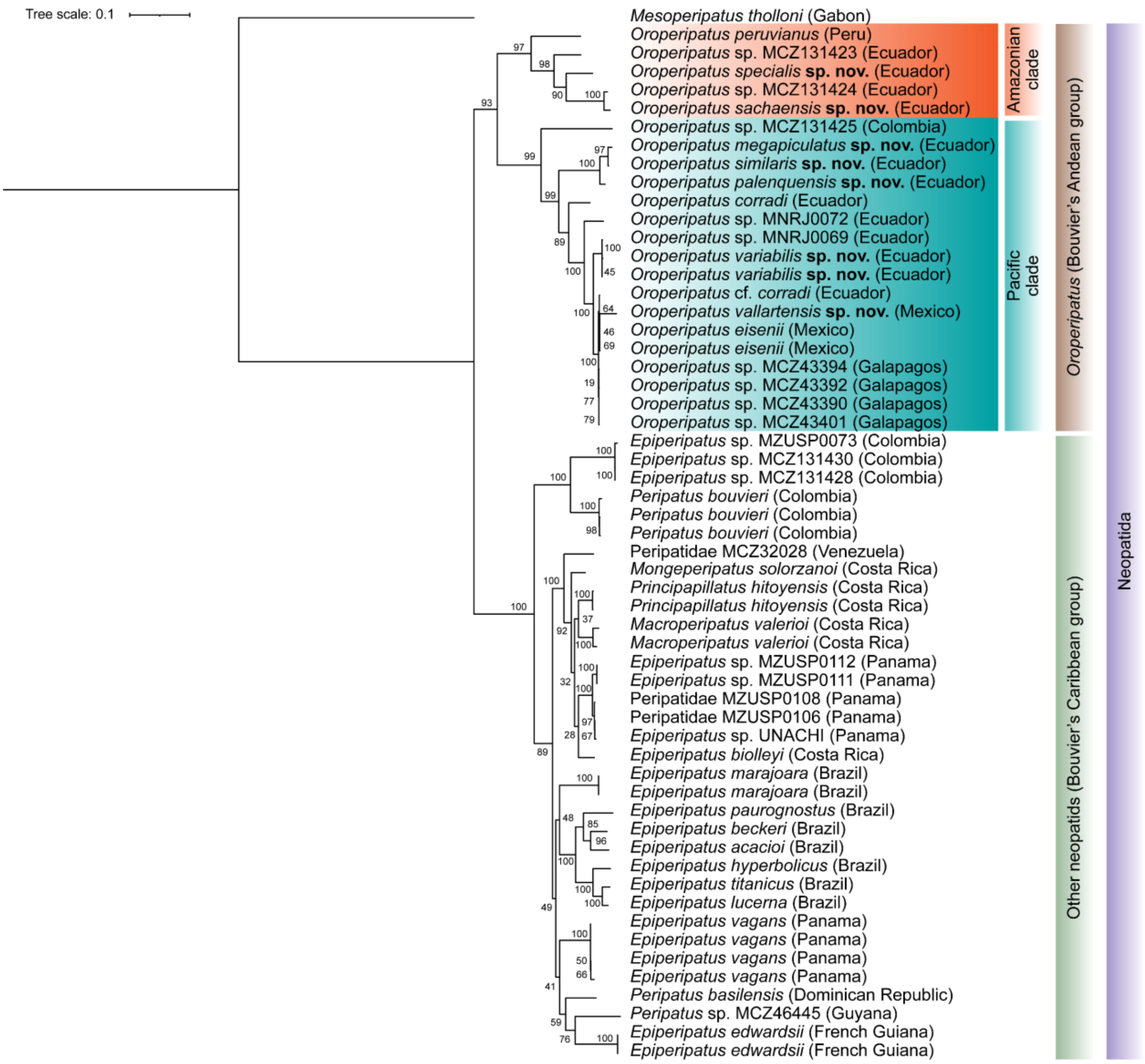

Figure S4. Phylogenetic tree showing the relationship of the studied species, Related to Results. Maximum likelihood topology combining nucleotide sequences of the *12S rRNA*, *16S rRNA*, *18S rRNA* genes and amino acid sequences of *COI* gene. Outgroup excluding peripatopsids and *Eoperipatus*. The type species *O. lankesteri* is not included. Numbers indicate bootstrap support values. Note the well-supported Amazonian (orange) and Pacific (blue) subclades of *Oroperipatus*. See also Figure 2, S1–S3, and S5–S9.

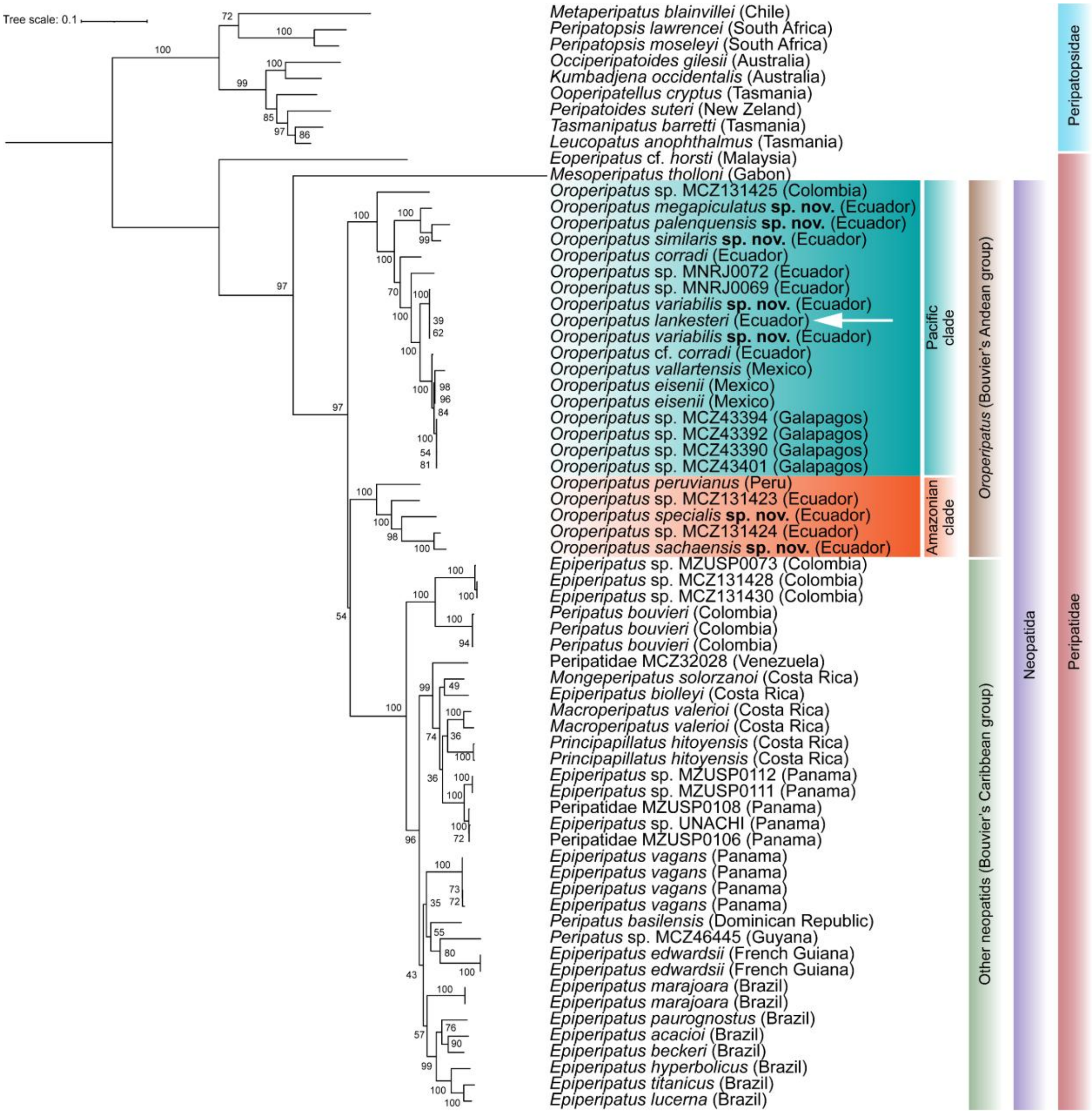

Figure S5. Phylogenetic relationships among studied *Oroperipatus* species, Related to Results. Maximum likelihood topology combining nucleotide sequences of the *12S rRNA*, *16S rRNA*, *18S rRNA* and *COI* genes. Outgroup including peripatopsids and *Eoperipatus*. Arrow point to the position of the type species *O. lankesteri*. Numbers indicate bootstrap support values. Amazonian (orange) and Pacific (blue) subclades are highlighted. Note the weakly supported paraphyletic topology of *Oroperipatus*. See also Figure 2, S1–S4, and S6–S9.

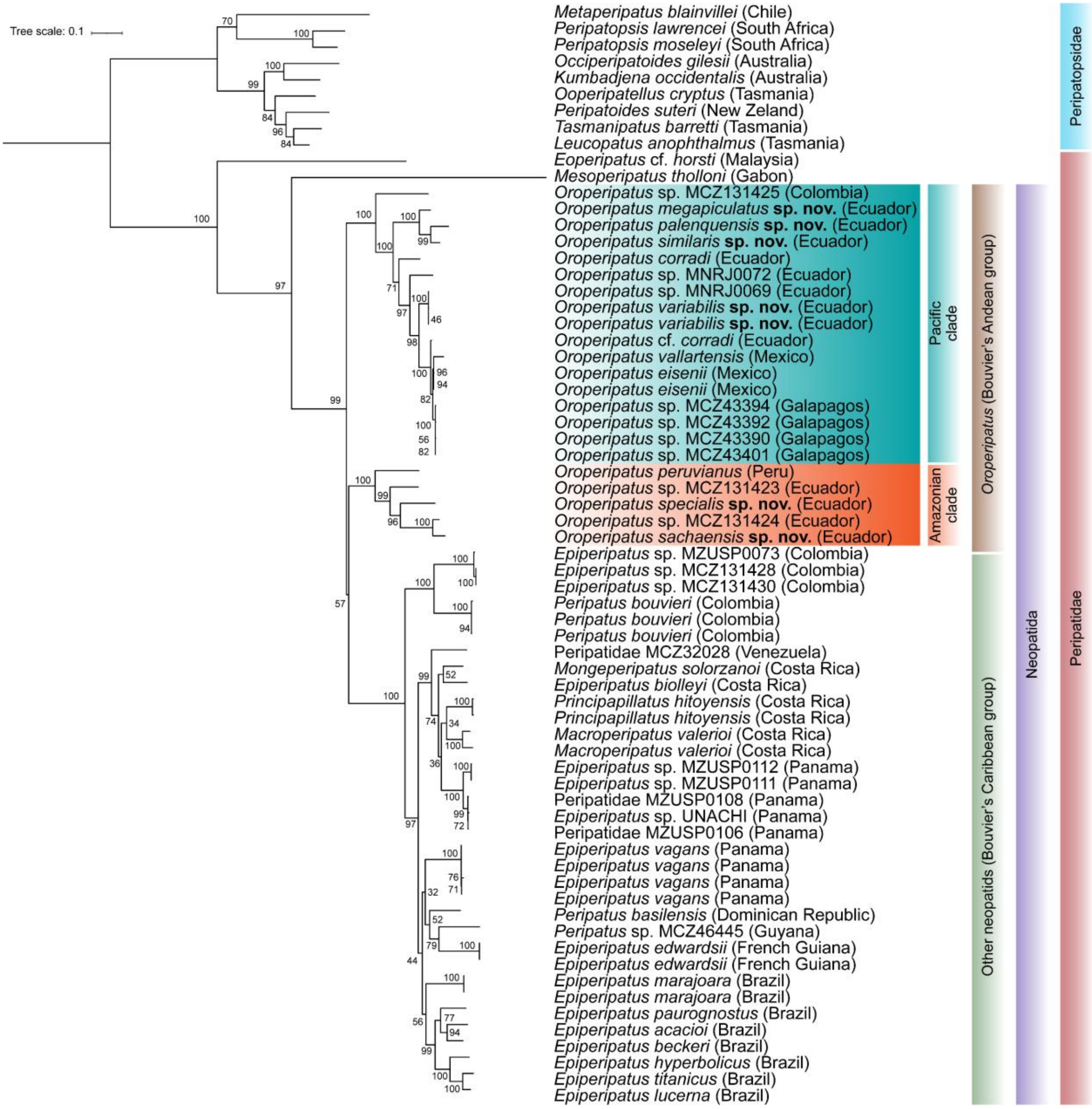

Figure S6. Phylogenetic relationships among studied *Oroperipatus* species, Related to Results. Maximum likelihood topology combining nucleotide sequences of the *12S rRNA*, *16S rRNA*, *18S rRNA* and *COI* genes. Outgroup including peripatopsids and *Eoperipatus*. The type species *O. lankesteri* is not included. Numbers indicate bootstrap support values. Amazonian (orange) and Pacific (blue) subclades are highlighted. Note the weakly supported paraphyletic topology of *Oroperipatus*. See also Figure 2, S1–S5, and S7–S9.

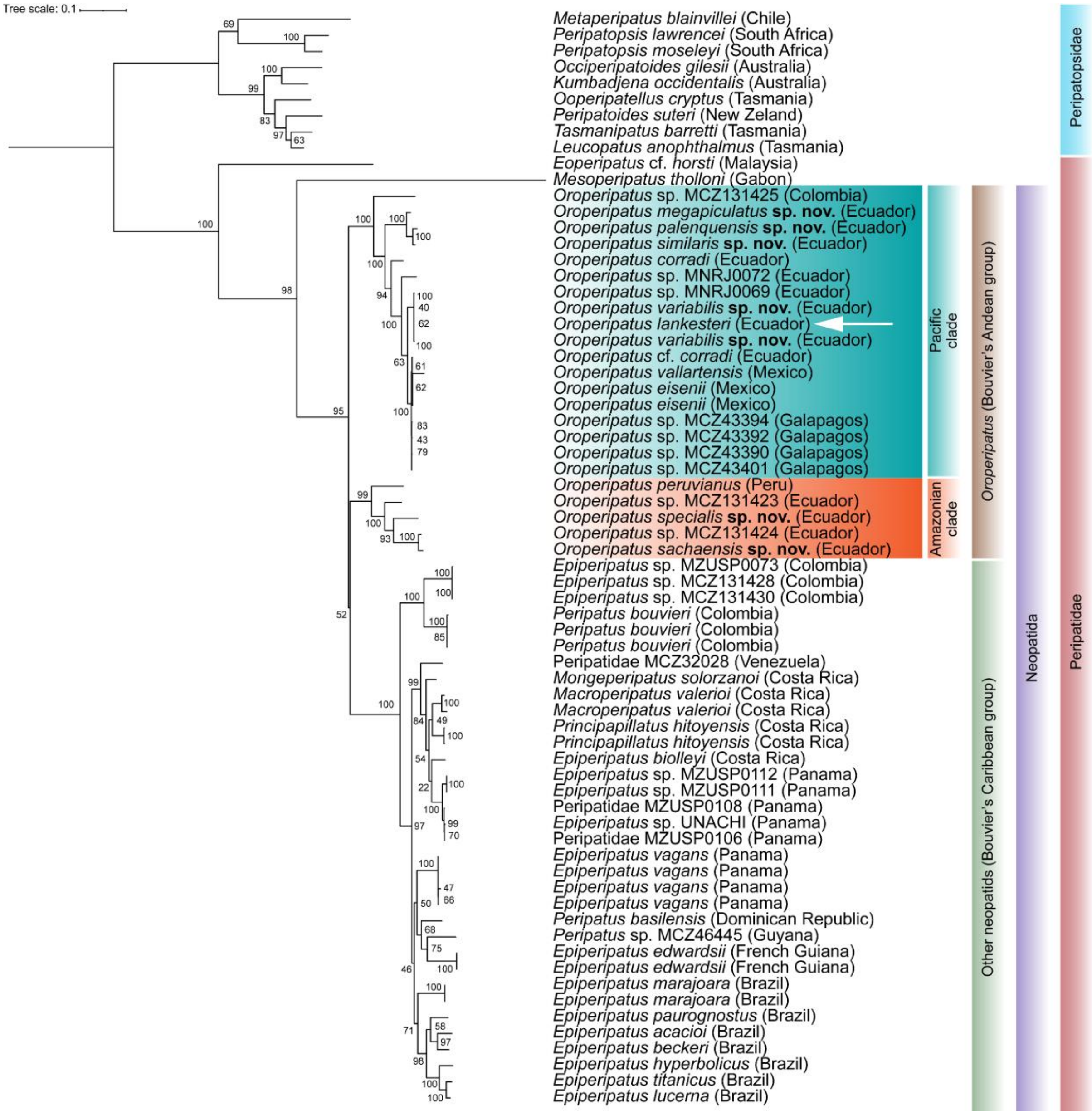

Figure S7. Phylogenetic relationships among studied *Oroperipatus* species, Related to Results. Maximum likelihood topology combining nucleotide sequences of the *12S rRNA*, *16S rRNA*, *18S rRNA* genes and *COI* gene sequences excluding the third codon positions. Outgroup including peripatopsids and *Eoperipatus*. Arrow point to the position of the type species *O. lankesteri*. Numbers indicate bootstrap support values. Amazonian (orange) and Pacific (blue) subclades are highlighted. Note the weakly supported paraphyletic topology of *Oroperipatus*. See also Figure 2, S1–S6, S8 and S9.

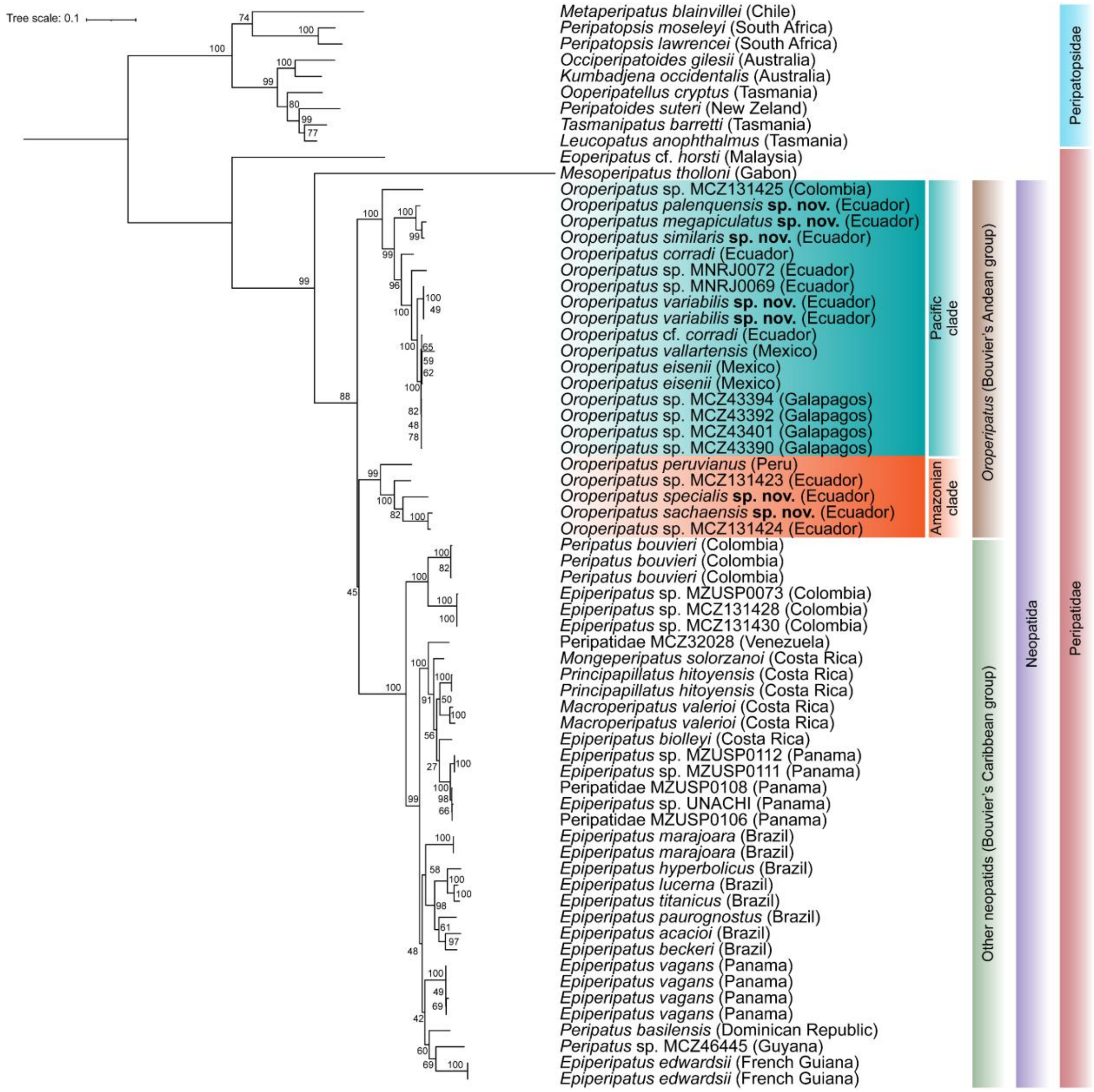

Figure S8. Phylogenetic relationships among studied *Oroperipatus* species, Related to Results. Maximum likelihood topology combining nucleotide sequences of the *12S rRNA*, *16S rRNA*, *18S rRNA* genes and *COI* gene sequences excluding the third codon positions. Outgroup including peripatopsids and *Eoperipatus*. The type species *O. lankesteri* is not included. Numbers indicate bootstrap support values. Amazonian (orange) and Pacific (blue) subclades are highlighted. Note the weakly supported paraphyletic topology of *Oroperipatus*. See also Figure 2, S1–S7 and S9.

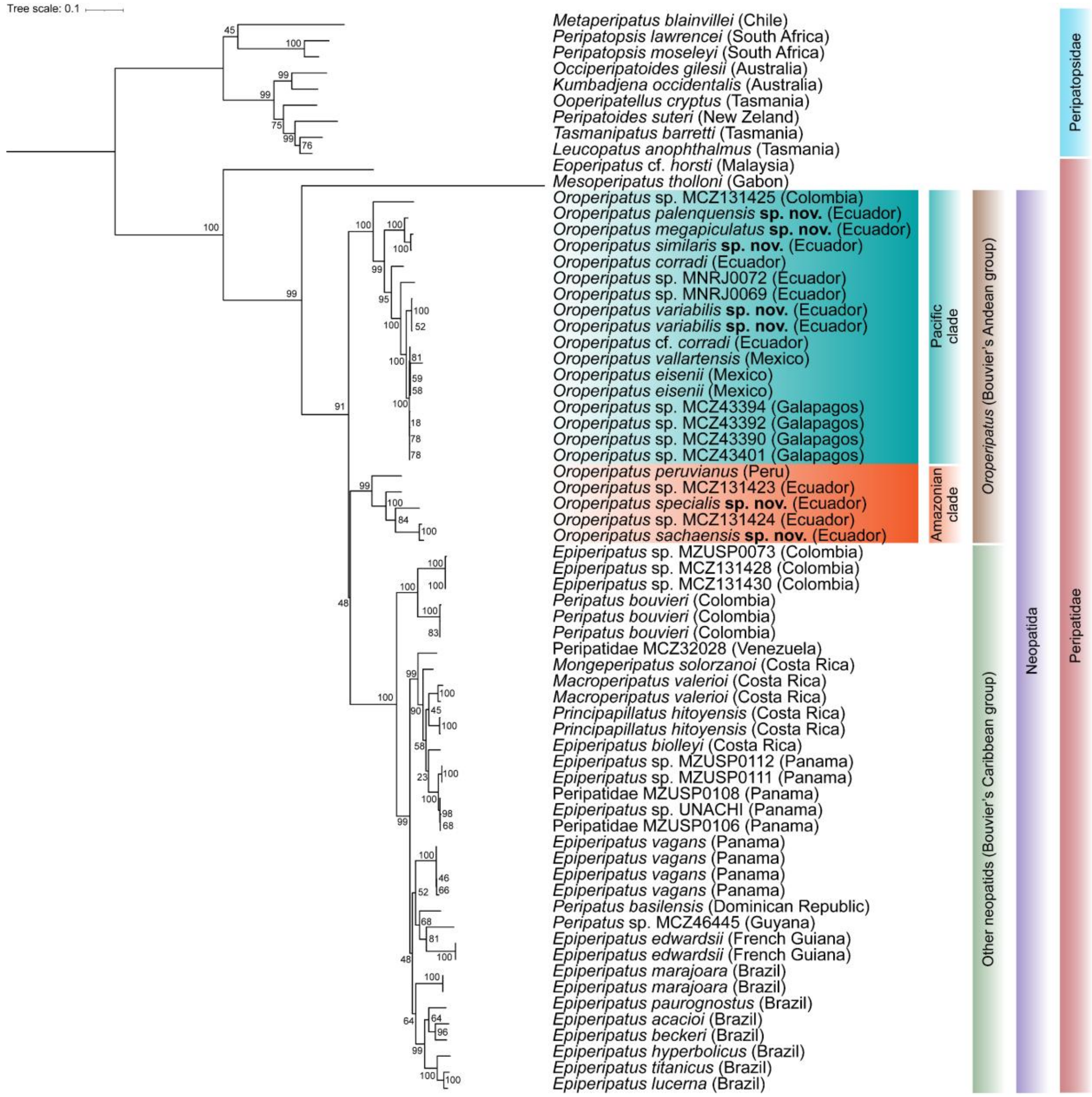

Figure S9. Phylogenetic relationships among studied *Oroperipatus* species, Related to Results. Maximum likelihood topology combining nucleotide sequences of the *12S rRNA*, *16S rRNA*, *18S rRNA* genes and amino acid sequences of *COI* gene. Outgroup including peripatopsids and *Eoperipatus*. The type species *O. lankesteri* is not included. Numbers indicate bootstrap support values. Amazonian (orange) and Pacific (blue) subclades are highlighted. Note the weakly supported paraphyletic topology of *Oroperipatus*. See also Figure 2, and S1–S8.

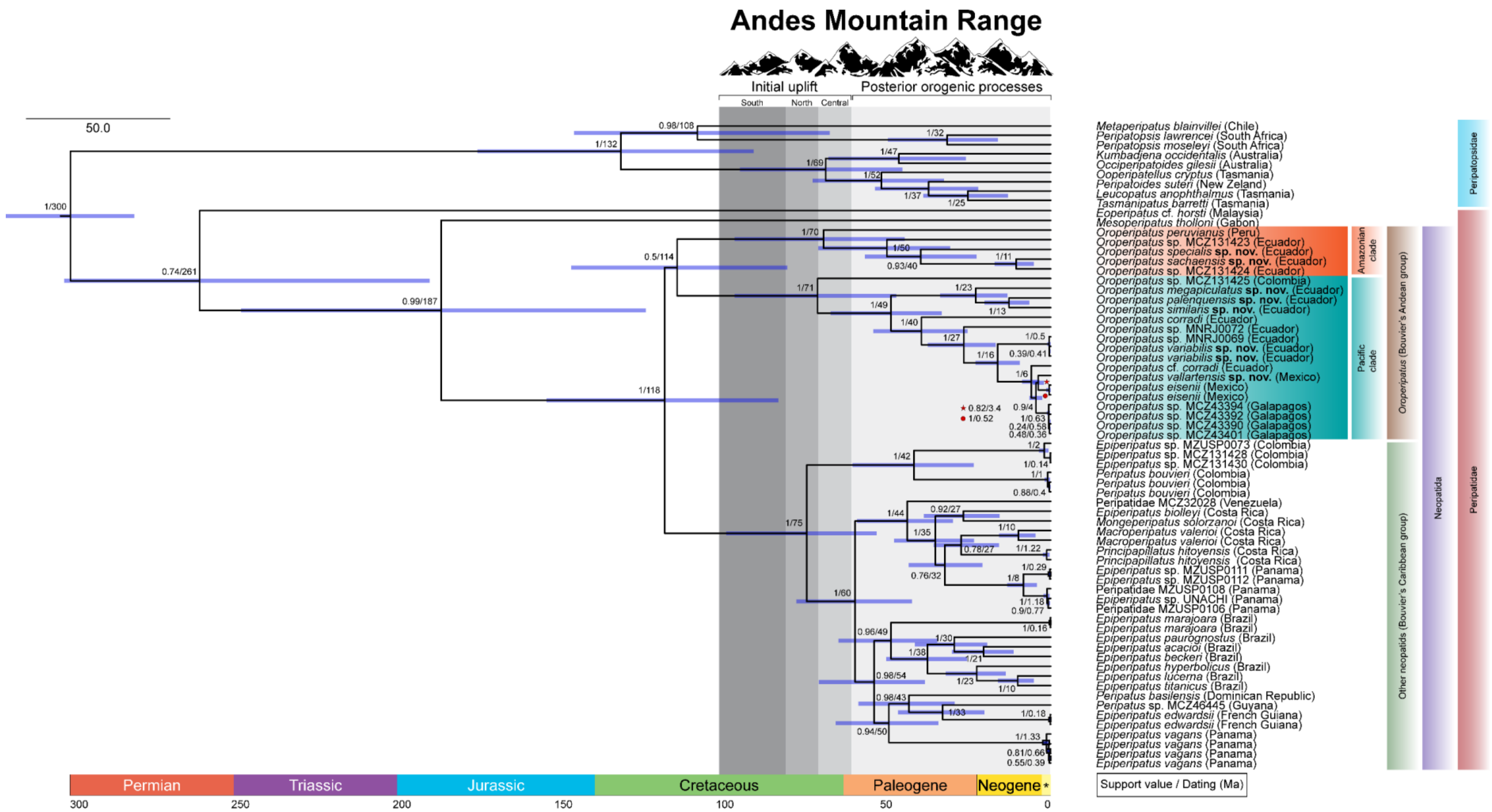

Figure S10. Chronogram of the complete dataset inferred in BEAST, Related to Results. Geological scale shown below, with an asterisk marking the Quaternary period; timing of Andean orogeny *sensu* Pérez-Escobar ([20]) is indicated above. Horizontal bars represent the 95% highest probability density (HPD) intervals for divergence times. The Amazonian (orange) and Pacific (blue) subclades of *Oroperipatus* are highlighted. See also Figure 3.

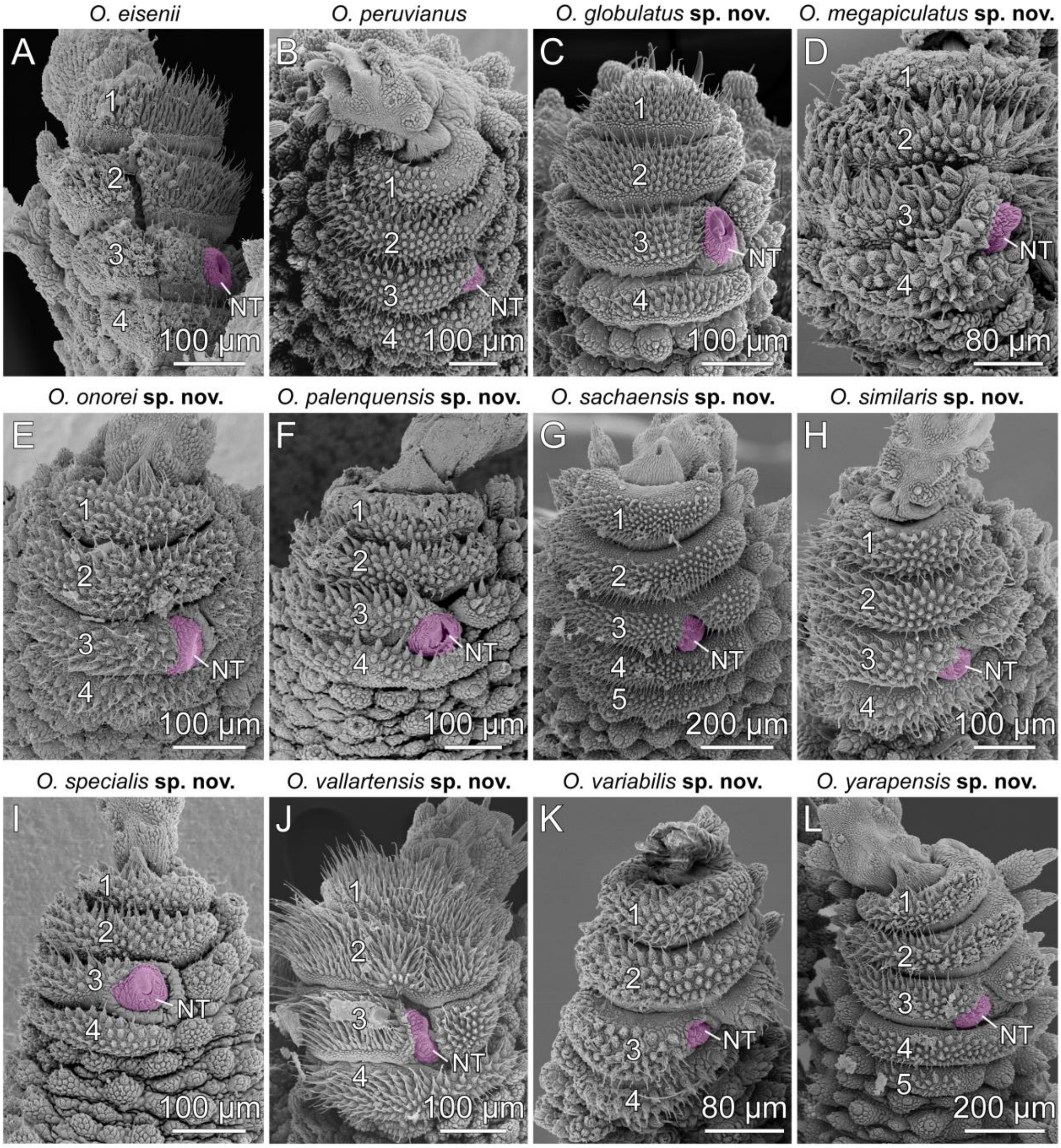

Figure S11. Nephridial tubercle on leg pair IV or V in the species examined, Related to Results. Scanning electron micrographs. Distal is up in all images. Spinous pads are numbered in the proximal direction. In *Oroperipatus*, the nephridial tubercle (artificially colored for clarity) consistently occupies a characteristic position on the third spinous pad, dividing this pad into two unequal parts. (A) *O. eisenii*. (B) *O. peruvianus*. (C) *O. globulatus* **sp. nov**. (D) *O. megapiculatus* **sp. nov**. (E) *O. onorei* **sp. nov**. (F) *O. palenquensis* **sp. nov**. (G) *O. sachaensis* **sp. nov**. (H) *O. similaris* **sp. nov**. (I) *O. specialis* **sp. nov**. (J) *O. vallartensis* **sp. nov**. (K) *O. variabilis* **sp. nov**. (L) *O. yarapensis* **sp. nov**. Abbreviations: NT, nephridial tubercle.

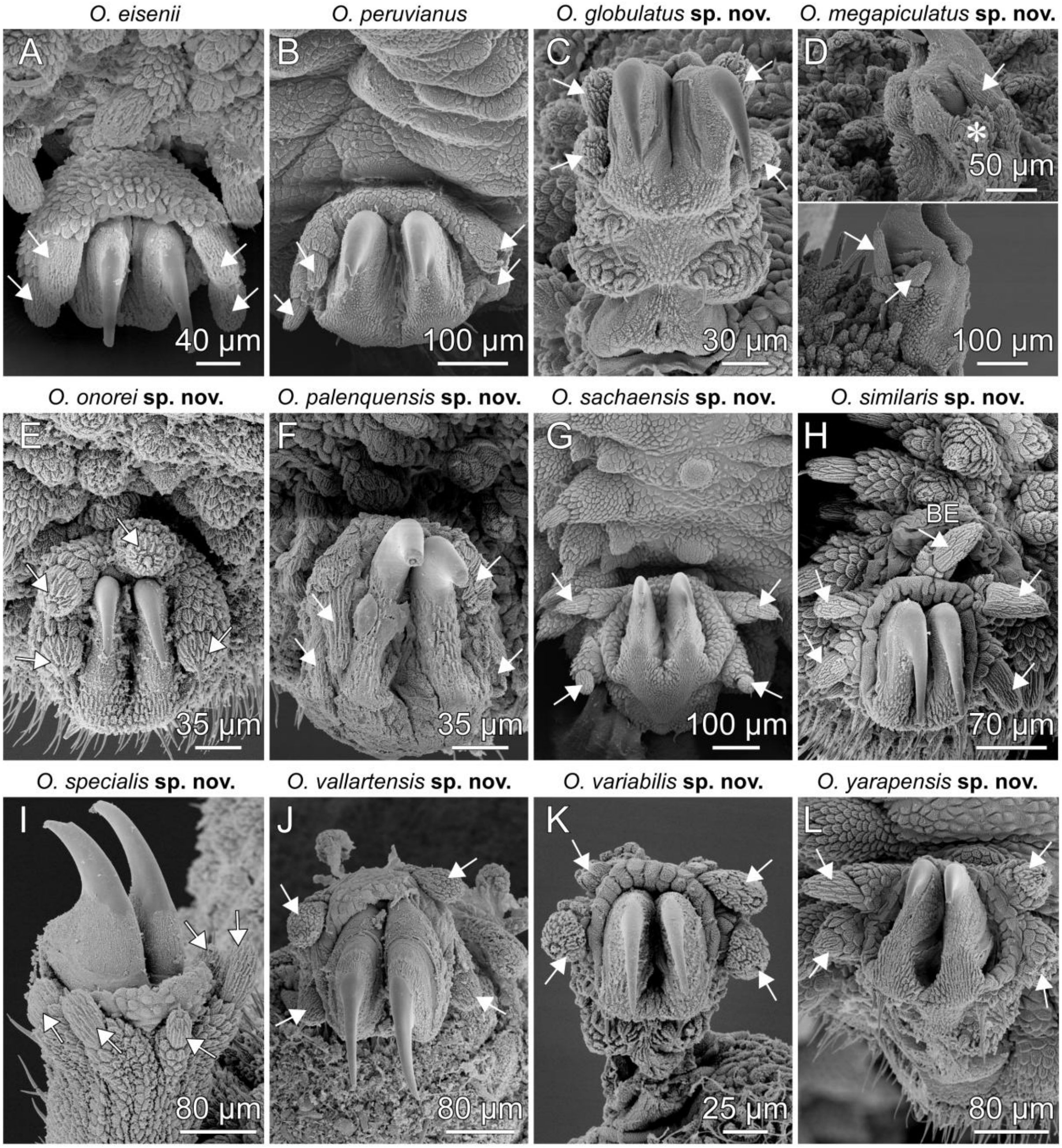

Figure S12. Distal foot papillae in the species examined, Related to Results. Scanning electron micrographs. Anterior is left in all images. Note the presence of at least two anterior and two posterior distal foot papillae (arrows) characteristic to *Oroperipatus*. (A) *O. eisenii*. (B) *O. peruvianus*. (C) *O. globulatus* **sp. nov**. (D) *O. megapiculatus* **sp. nov**. Anterior (up) and posterior (down) views. Asterisk indicates a broken distal foot papilla. (E) *O. onorei* **sp. nov**. (F) *O. palenquensis* **sp. nov**. (G) *O. sachaensis* **sp. nov**. (H) *O. similaris* **sp. nov**. (I) *O. specialis* **sp. nov**. (J) *O. vallartensis* **sp. nov**. (K) *O. variabilis* **sp. nov**. (L) *O. yarapensis* **sp. nov**. Abbreviation: BE, bean-shaped papilla.

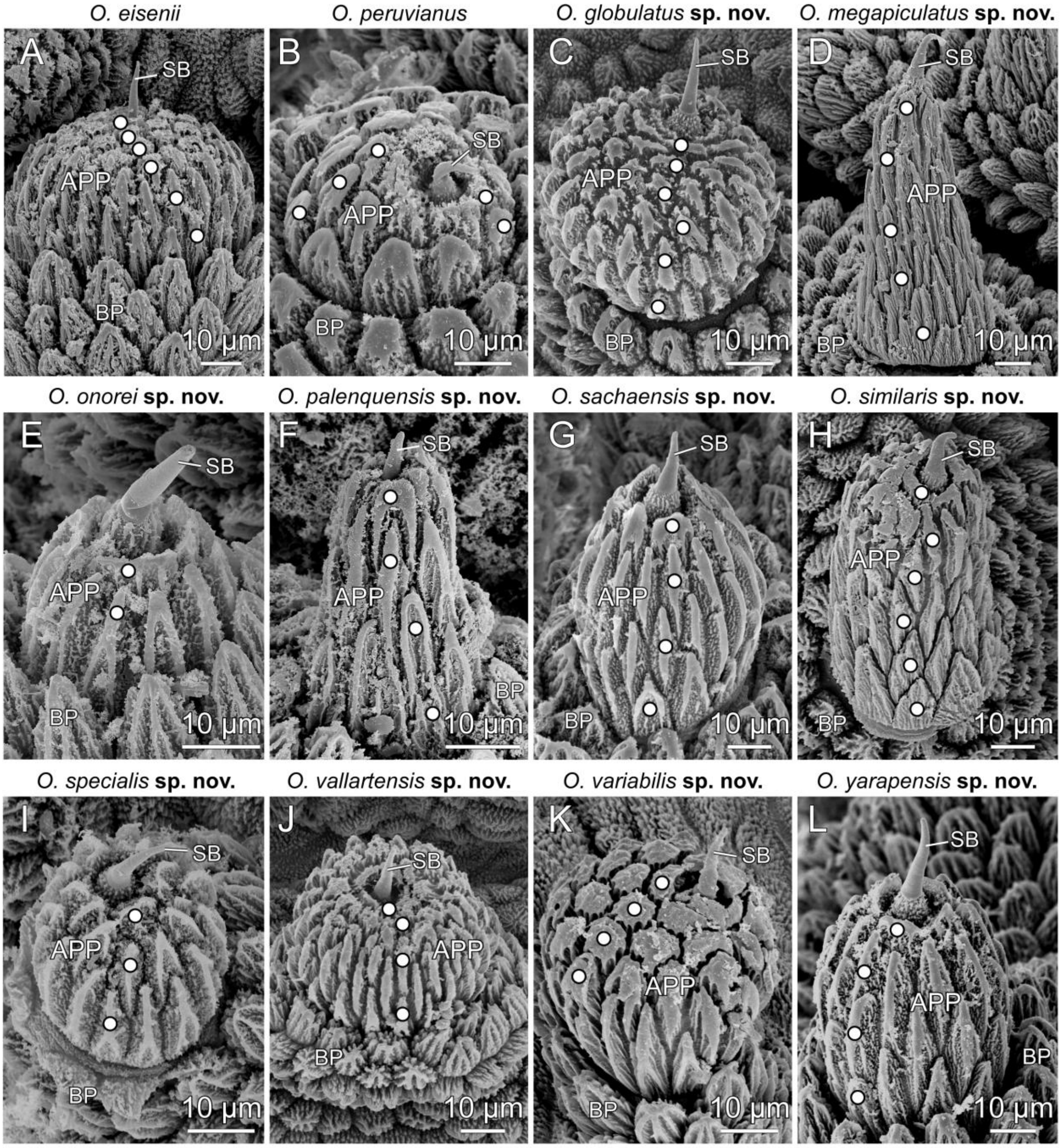

Figure S13. Apical piece of dorsal primary papillae in the species examined, Related to Results. Scanning electron micrographs. Dorsal is up in all images. White dots indicate the scale ranks. (A) *O. eisenii*. (B) *O. peruvianus*. (C) *O. globulatus* **sp. nov**. (D) *O. megapiculatus* **sp. nov**. (E) *O. onorei* **sp. nov**. (F) *O. palenquensis* **sp. nov**. (G) *O. sachaensis* **sp. nov**. (H) *O. similaris* **sp. nov**. (I) *O. specialis* **sp. nov**. (J) *O. vallartensis* **sp. nov**. (K) *O. variabilis* **sp. nov**. (L) *O. yarapensis* **sp. nov**. Abbreviations: APP, apical piece; BP, basal piece; SB, sensory bristle.

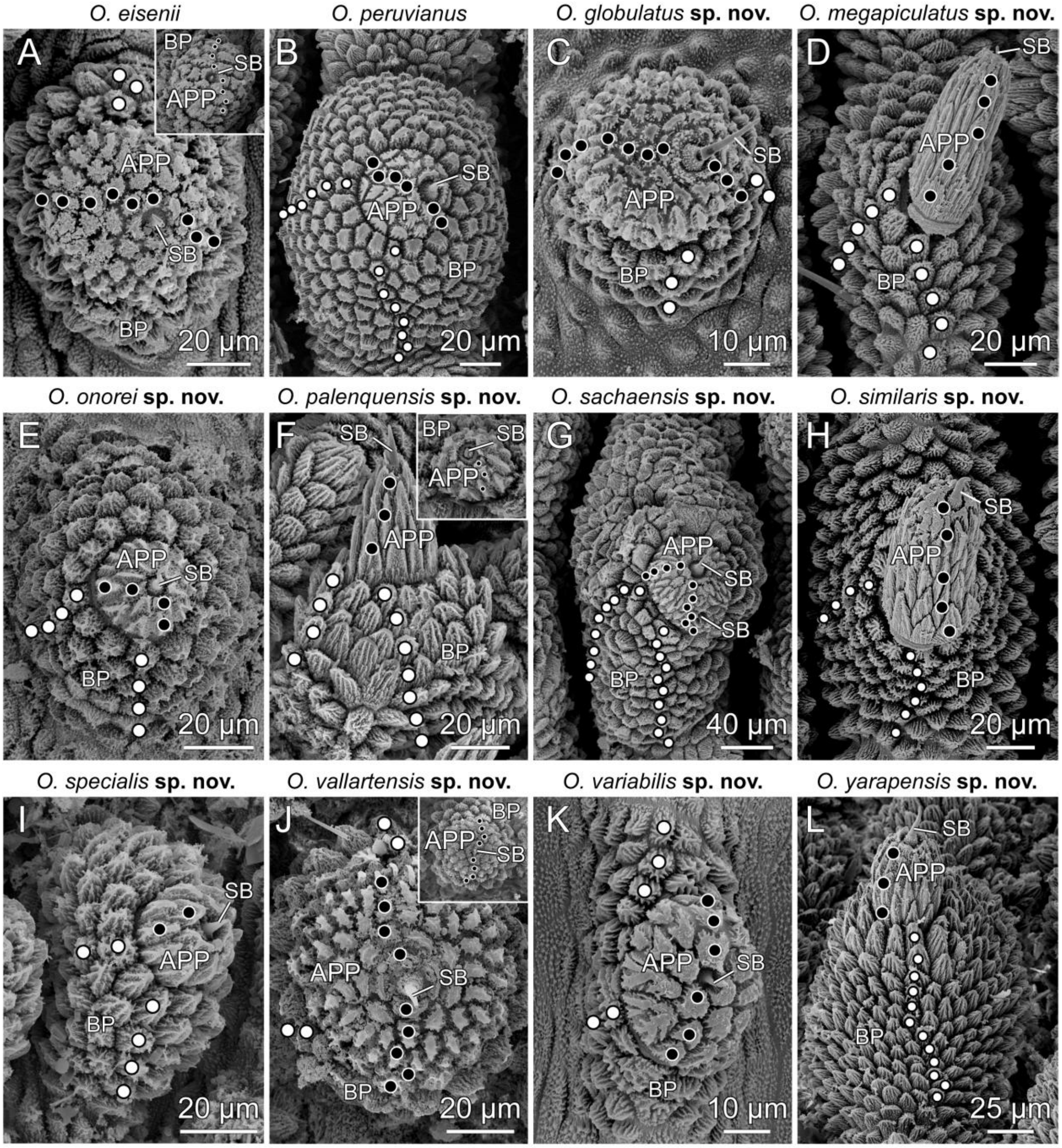

Figure S14. Dorsal primary papillae in the species examined, Related to Results. Scanning electron micrographs. Scale ranks are indicated by black dots on the apical pieces and white dots on the basal pieces. (A) *O. eisenii*. (B) *O. peruvianus*. (C) *O. globulatus* **sp. nov**. (D) *O. megapiculatus* **sp. nov**. (E) *O. onorei* **sp. nov**. (F) *O. palenquensis* **sp. nov**. (G) *O. sachaensis* **sp. nov**. Note the rare presence of two sensory bristles on the apical piece. (H) *O. similaris* **sp. nov**. (I) *O. specialis* **sp. nov**. (J) *O. vallartensis* **sp. nov**. (K) *O. variabilis* **sp. nov**. (L) *O. yarapensis* **sp. nov**. Insets in A,F,J show morphological variations of primary papillae in these species. Abbreviations: APP, apical piece; BP, basal piece; SB, sensory bristle.

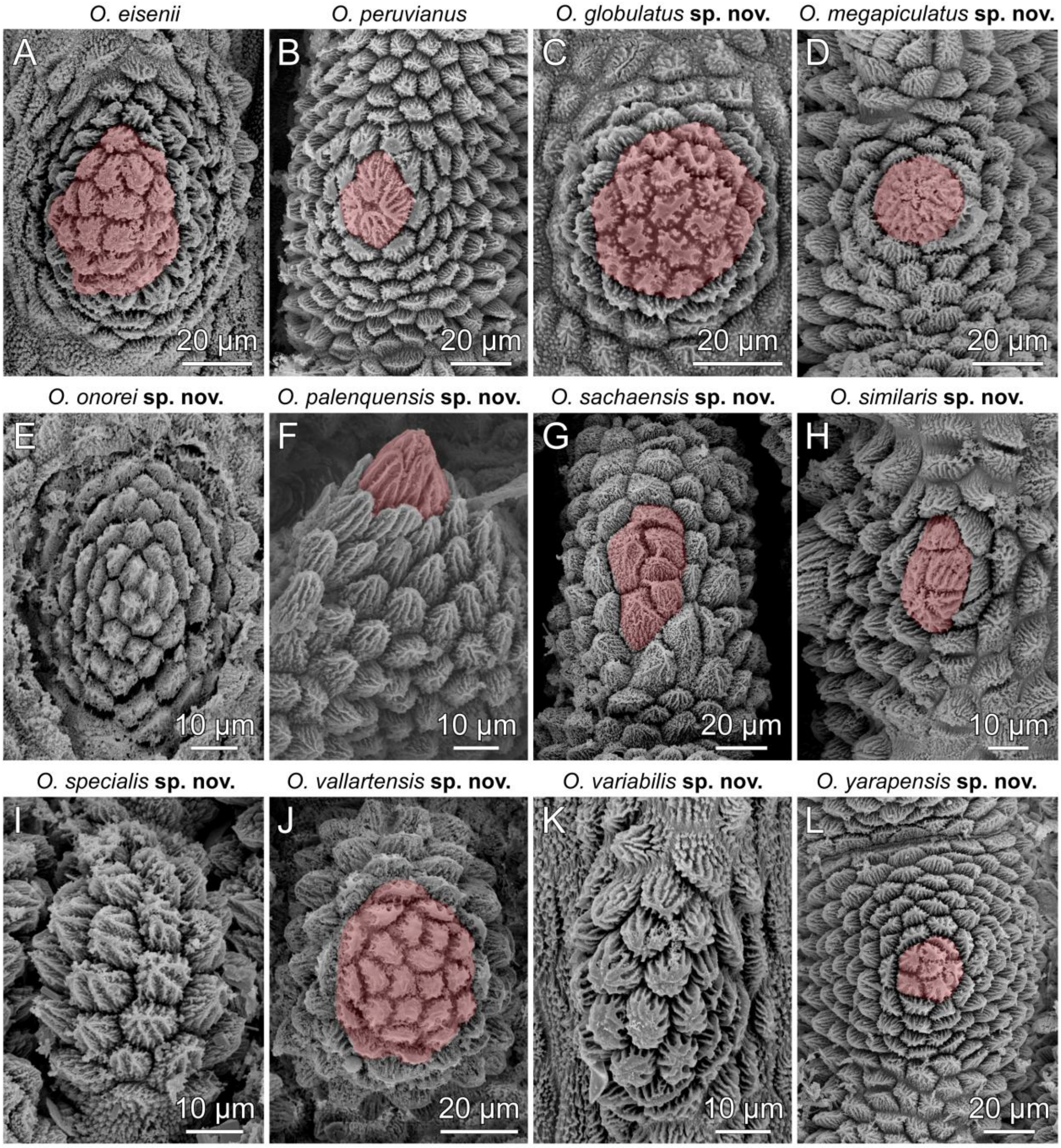

Figure S15. Dorsal accessory papillae in the species examined, Related to Results. Scanning electron micrographs. (A) *O. eisenii*. (B) *O. peruvianus*. (C) *O. globulatus* **sp. nov**. (D) *O. megapiculatus* **sp. nov**. (E) *O. onorei* **sp. nov**. (F) *O. palenquensis* **sp. nov**. (G) *O. sachaensis* **sp. nov**. (H) *O. similaris* **sp. nov**. (I) *O. specialis* **sp. nov**. (J) *O. vallartensis* **sp. nov**. (K) *O. variabilis* **sp. nov**. (L) *O. yarapensis* **sp. nov**. Note the modified apical most scales in some species (artificially colored for clarity).

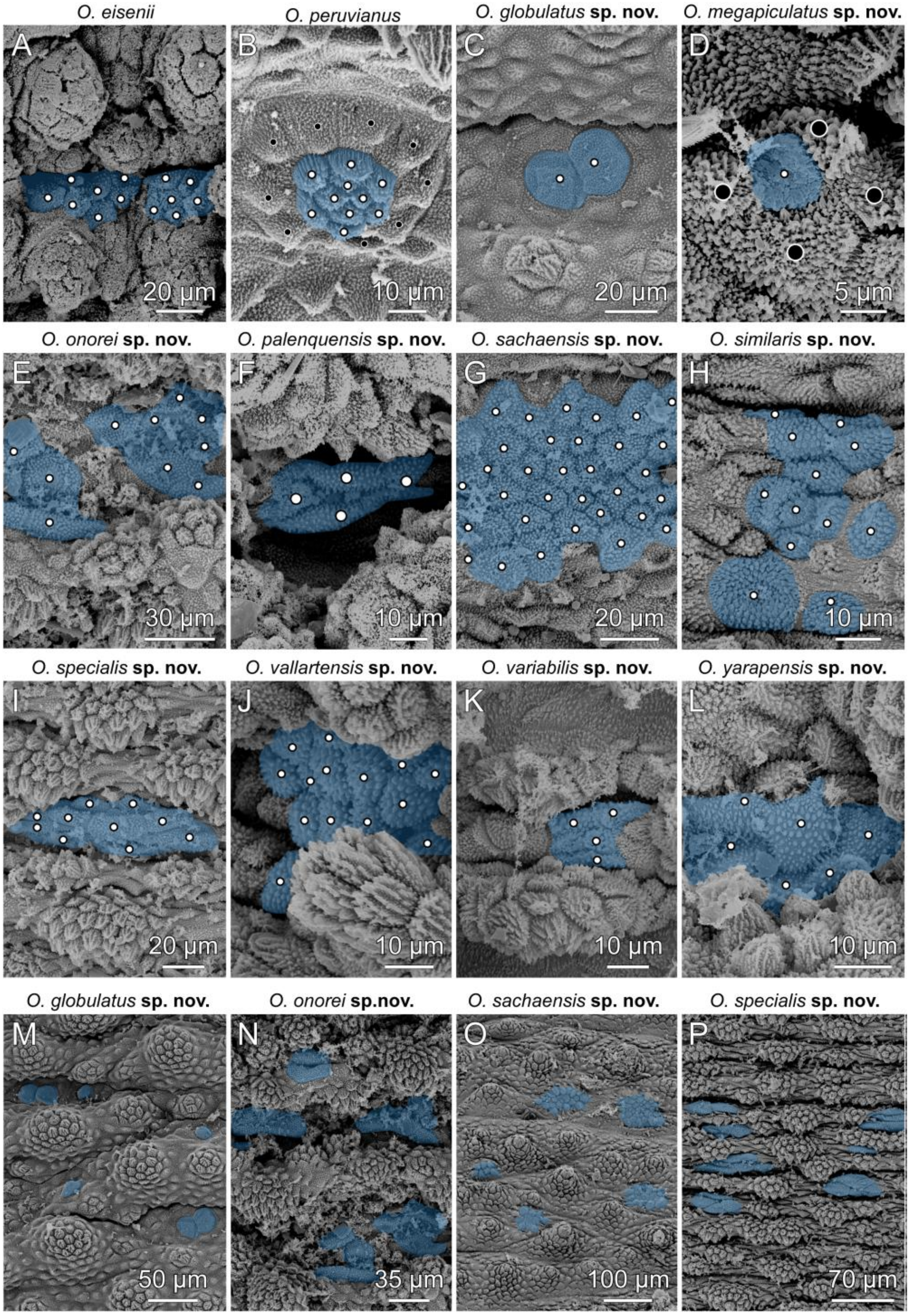

Figure S16. Interpedal structures in the species examined, Related to Results. Scanning electron micrographs. Interpedal structures artificially colored for clarity. (A–L) Detail of single interpedal structures. White dots indicate scales composing the interpedal structures; black dots mark the textured scales surrounding the interpedal structures in some species. (A) *O. eisenii*. (B) *O. peruvianus*. (C) *O. globulatus* **sp. nov**. (D) *O. megapiculatus* **sp. nov**. (E) *O. onorei* **sp. nov**. (F) *O. palenquensis* **sp. nov**. (G) *O. sachaensis* **sp. nov**. (H) *O. similaris* **sp. nov**. (I) *O. specialis* **sp. nov**. (J) *O. vallartensis* **sp. nov**. (K) *O. variabilis* **sp. nov**. (L) *O. yarapensis* **sp. nov**. Insets in A,J show variations in shape and number of scales in the Mexican species. (M–P) Overview of the number of interpedal structures in *O. globulatus* **sp. nov.** (M), *O. onorei* **sp. nov.** (N), *O. sachaensis* **sp. nov.** (O), and *O. specialis* **sp. nov.** (P).

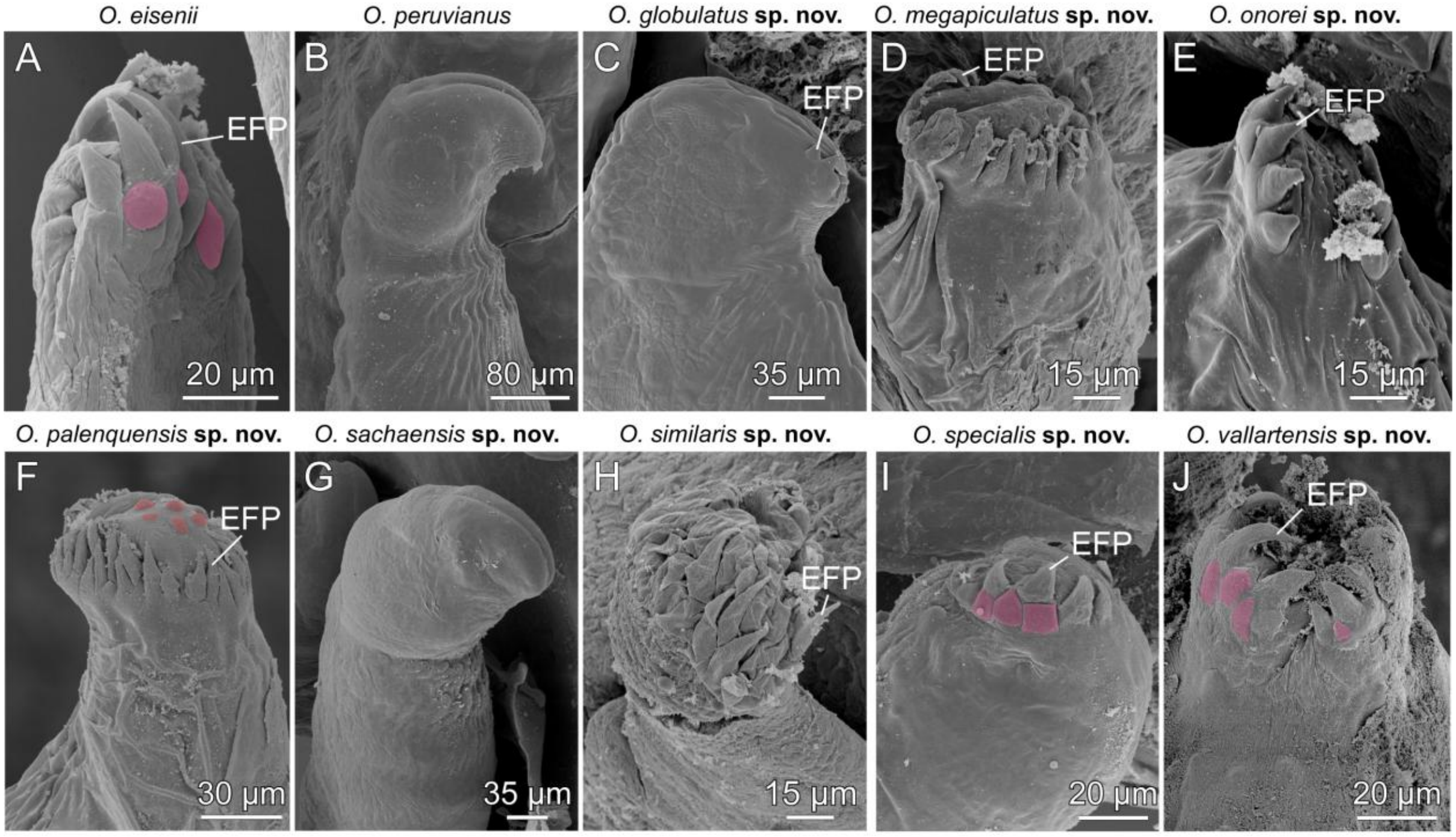

Figure S17. Embryonic foot projections in the species examined, Related to Results. Scanning electron micrographs. (A) *O. eisenii*. (B) *O. peruvianus*. (C) *O. globulatus* **sp. nov**. (D) *O. megapiculatus* **sp. nov**. (E) *O. onorei* **sp. nov**. (F) *O. palenquensis* **sp. nov**. (G) *O. sachaensis* **sp. nov**. (H) *O. similaris* **sp. nov**. (I) *O. specialis* **sp. nov**. (J) *O. vallartensis* **sp. nov**. Note the absence of embryonic foot projections in B, G, as well as the apical-most swellings in F and the polygonal to round basal bulks in A,I,J **(**artificially colored red and magenta respectively for clarity). Abbreviation: EFP, embryonic foot projection. See also Figure 5.

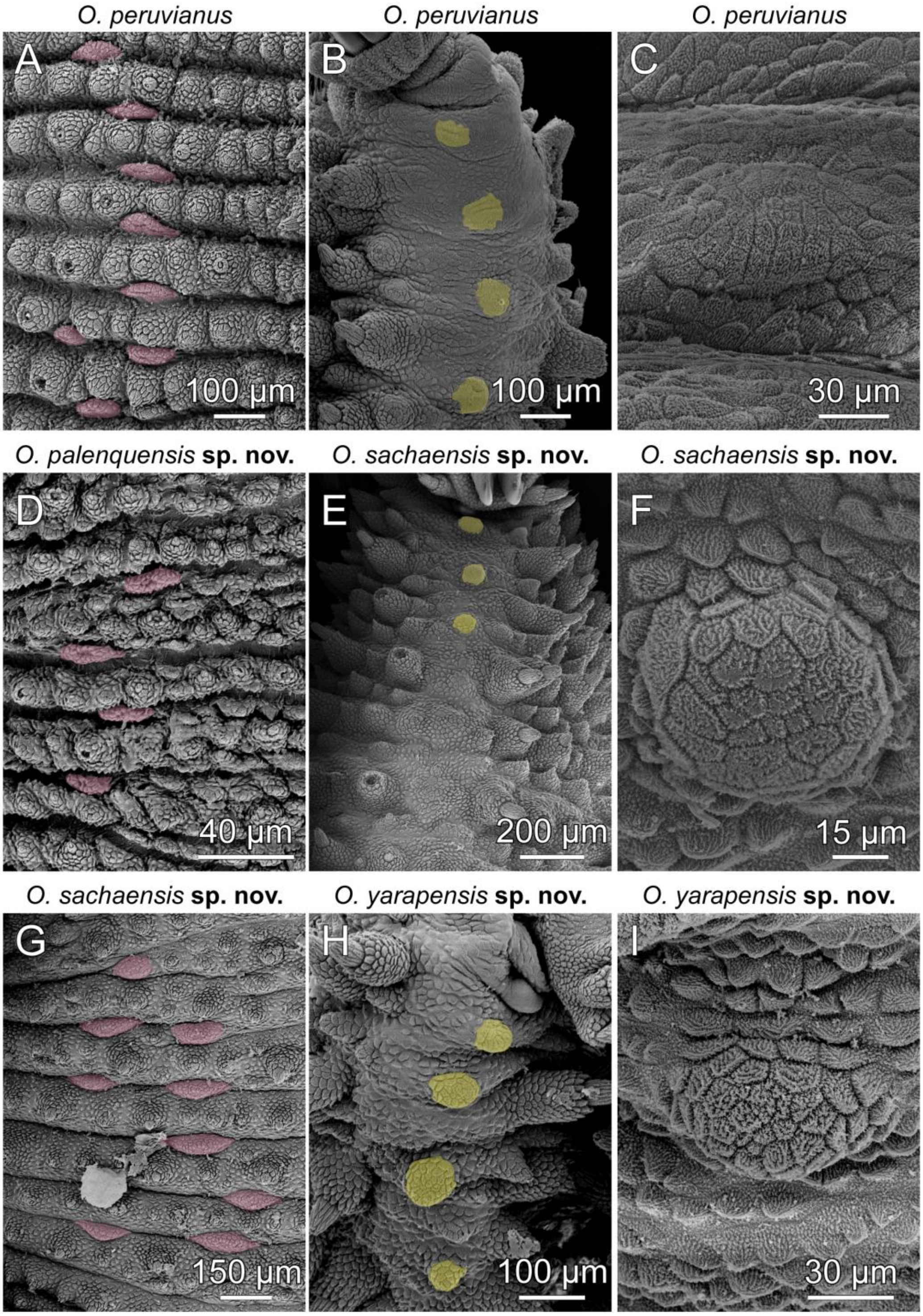

Figure S18. Species-specific features associated with type II crater-shaped papillae and the dorsodistal leg portion, Related to Results. Scanning electron micrographs. (A,D,G) Atypical number and arrangement of type II crater-shaped papillae (artificially colored for clarity) in *O. peruvianus* (A), *O. palenquensis* (D) and *O. sachaensis* (G). (B,E,H) Dorsodistal leg region with markedly reduced dermal papillae or lacking them in *O. peruvianus* (B), *O. sachaensis* **sp. nov.** (E) and *O. yarapensis* **sp. nov.** (H). Note the presence of round bumps in this area (artificially colored for clarity). (C,F,I) Details of a round bump in *O. peruvianus* (C), *O. sachaensis* **sp. nov.** (F) and *O. yarapensis* **sp. nov.** (I). See also Figure 5, S19 and S21.

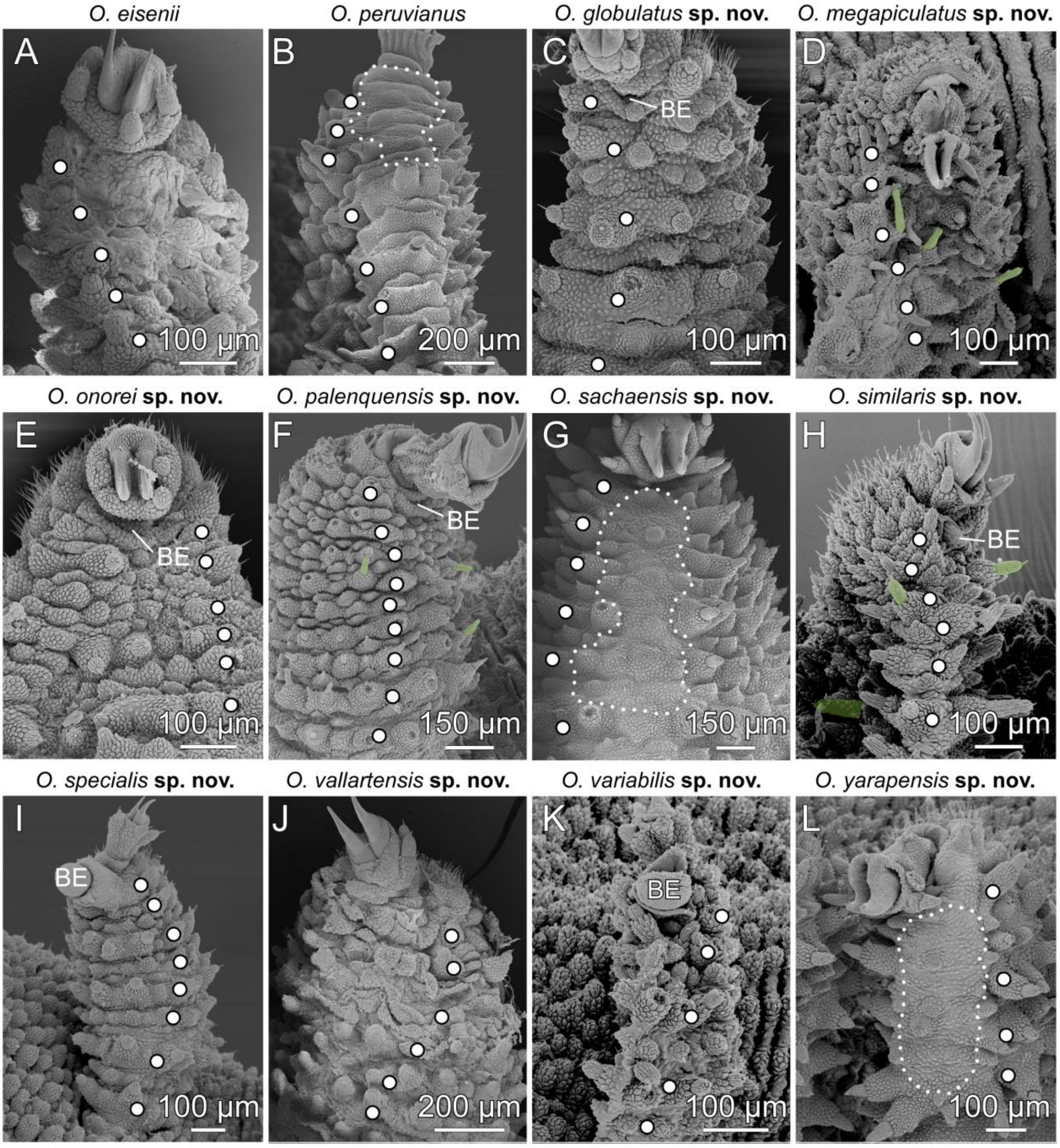

Figure S19. Dorsal leg region in the species examined, Related to Results. Scanning electron micrographs. White dots indicate the number of leg rings. Dotted lines in B,G,L indicate the region where dermal papillae are absent or markedly reduced. Note the elongated apical piece of primary papillae in D,F,H (artificially colored for clarity). (A) *O. eisenii*. (B) *O. peruvianus*. (C) *O. globulatus* **sp. nov**. (D) *O. megapiculatus* **sp. nov**. (E) *O. onorei* **sp. nov**. (G) *O. sachaensis* **sp. nov**. (H) *O. similaris* **sp. nov**. (I) *O. specialis* **sp. nov**. Note the modified bean-shaped papilla. (J) *O. vallartensis* **sp. nov**. (K) *O. variabilis* **sp. nov**. (L) *O. yarapensis* **sp. nov**. Abbreviations: BE, bean-shaped papilla. See also Figure 5, S18 and S21.

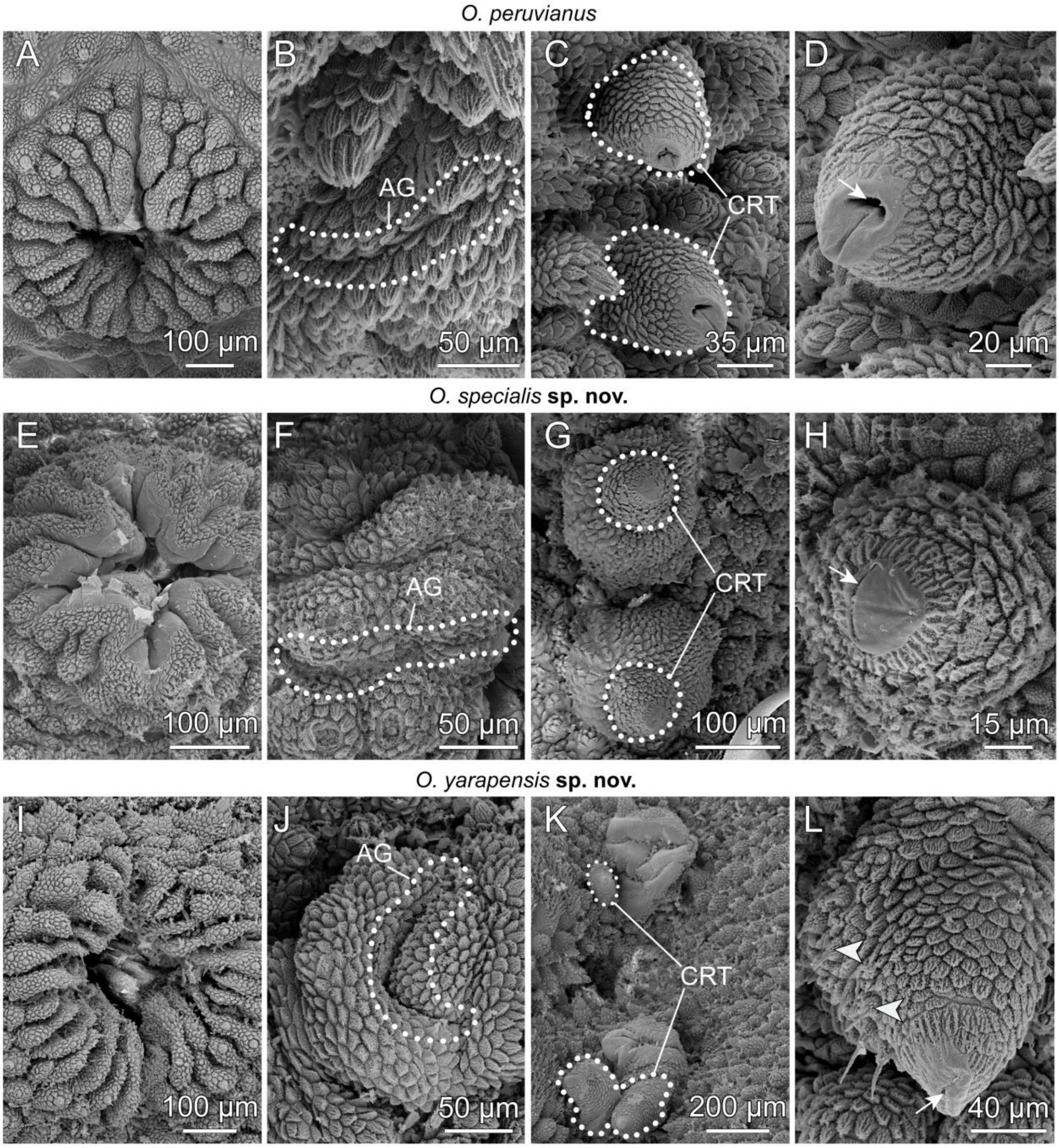

Figure S20. Male reproductive structures in the species examined, Related to Results. Scanning electron micrographs of *O. peruvianus* (A–D), *O. specialis* **sp. nov.** (E–H) and *O. yarapensis* **sp. nov.** (I–L). (A,E,I) Genital opening. (B,F,J) Detail of a single anal gland opening (dotted line). (C,G,K) Crural tubercles on the two pregenital leg pairs. (D,H,L) Detail of a single crural tubercle. Arrows point to the crural gland opening. Arrowheads indicate sensory bristles on the crural tubercles of *O. yarapensis* **sp. nov.** (L). Abbreviations: AG, anal gland; CRT, crural tubercle.

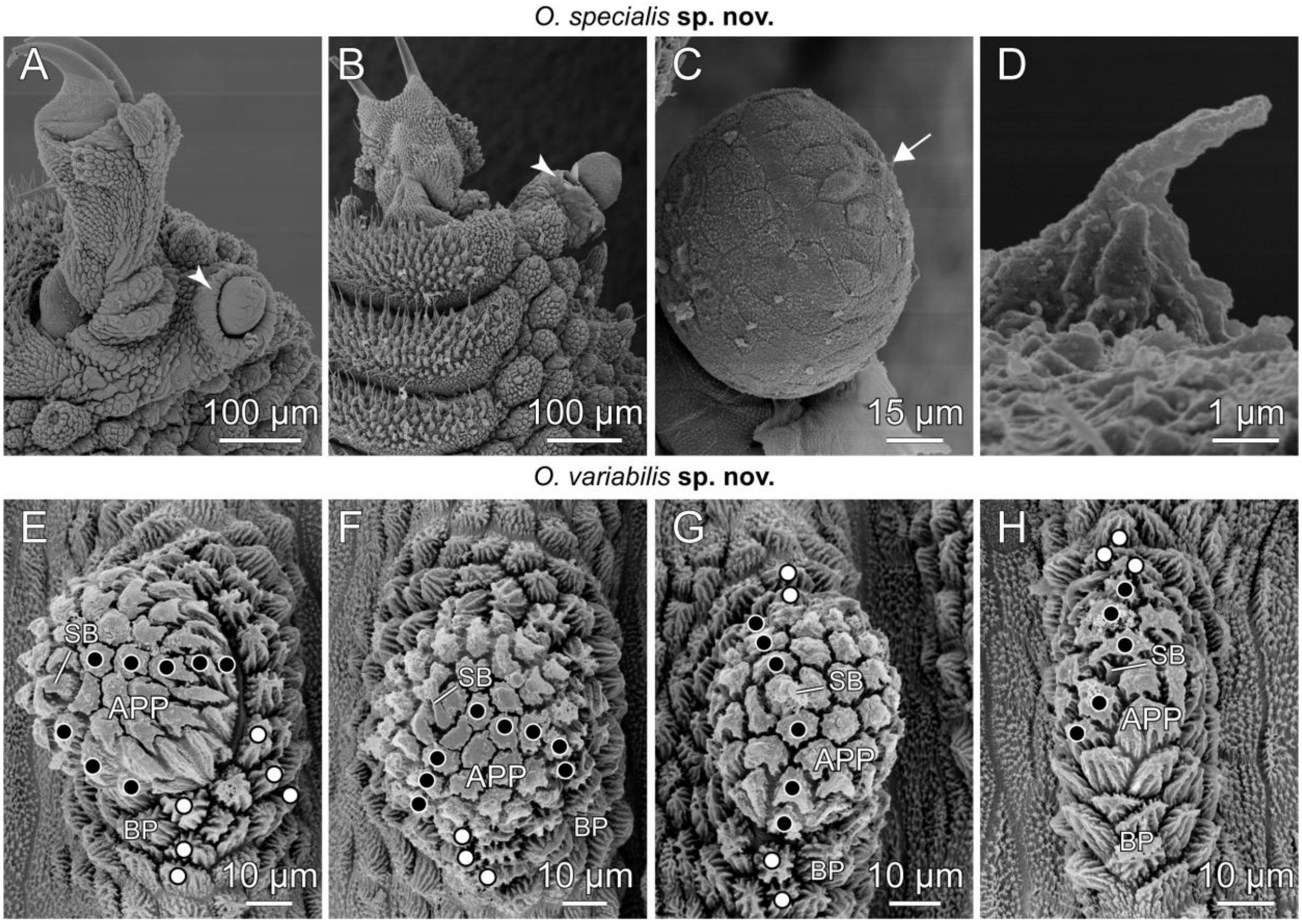

Figure S21. Remarkable diagnostic features identified in this study, Related to Results. Scanning electron micrographs. (A–D) Modified bean-shaped papilla (Arrowhead) in *O. specialis* **sp. nov**. in retracted (A) and everted (B) condition. (C,D) Spherical apex of the modified bean-shaped papilla (C) bearing a sensory bristle (arrow in C; D). (E–H) Morphological variation of dorsal primary papillae in *O. variabilis* **sp. nov**. Black dots mark scale ranks on apical piece, white dots correspond to scales ranks on basal piece. (E) Largest papilla with globular, asymmetrical apical piece. (F) Large papilla with semi-oval, asymmetrical apical piece. (G) Mid-sized papillae with round, symmetrical apical piece. (H) Small papillae with inconspicuous, asymmetrical apical piece. Abbreviations: APP, apical piece; BP, basal piece; SB, sensory bristle. See also Figure 5, S18 and S19.

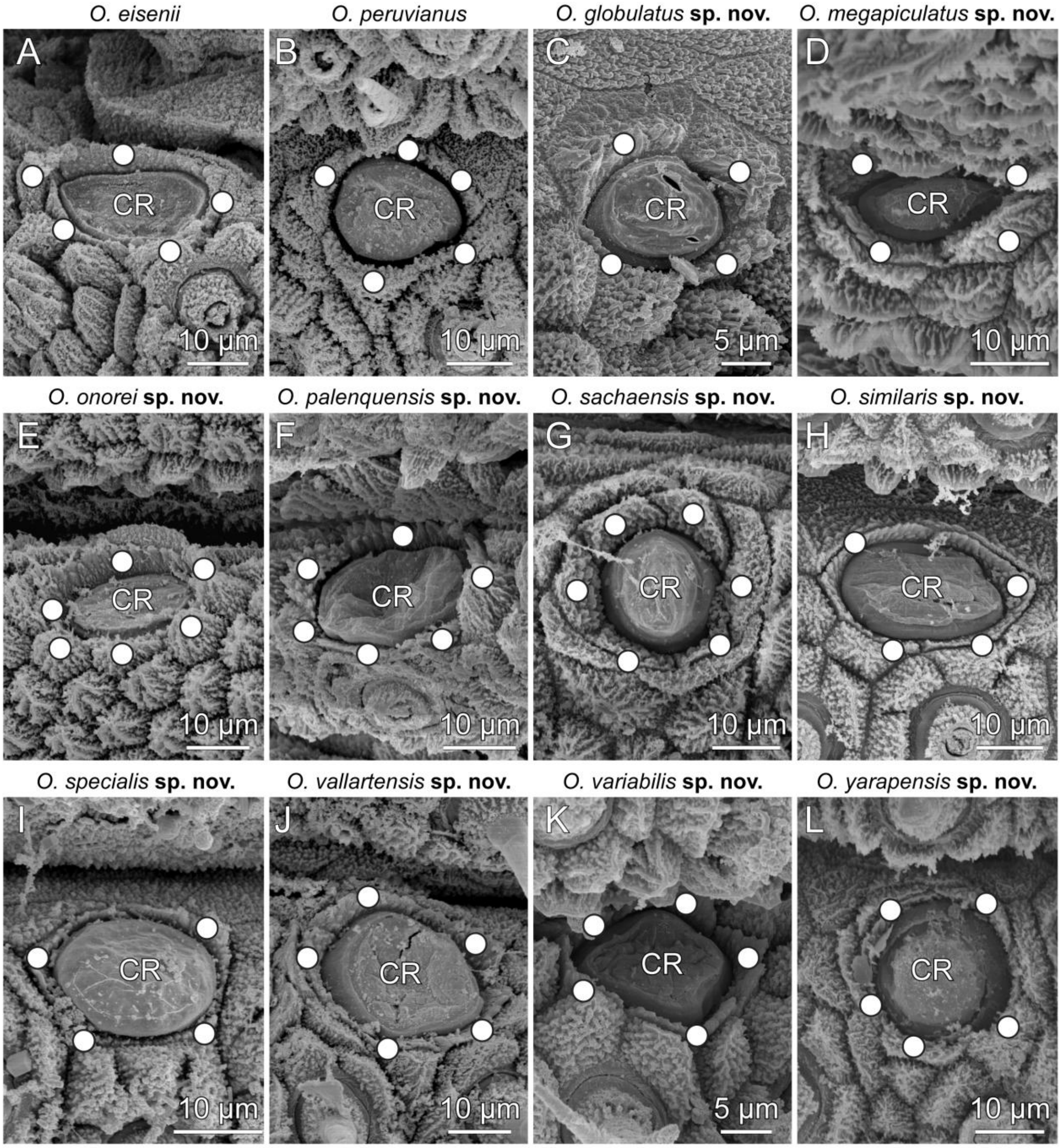

Figure S22. Chemoreceptor of the antennal tip in the species examined, Related to Results. Scanning electron micrographs. White dots indicate scales surrounding the chemoreceptor. (A) *O. eisenii*. (B) *O. peruvianus*. (C) *O. globulatus* **sp. nov**. (D) *O. megapiculatus* **sp. nov**. (E) *O. onorei* **sp. nov**. (F) *O. palenquensis* **sp. nov**. (G) *O. sachaensis* **sp. nov**. (H) *O. similaris* **sp. nov**. (I) *O. specialis* **sp. nov**. (J) *O. vallartensis* **sp. nov**. (K) *O. variabilis* **sp. nov**. (L) *O. yarapensis* **sp. nov**. Abbreviation: CR, chemoreceptor.

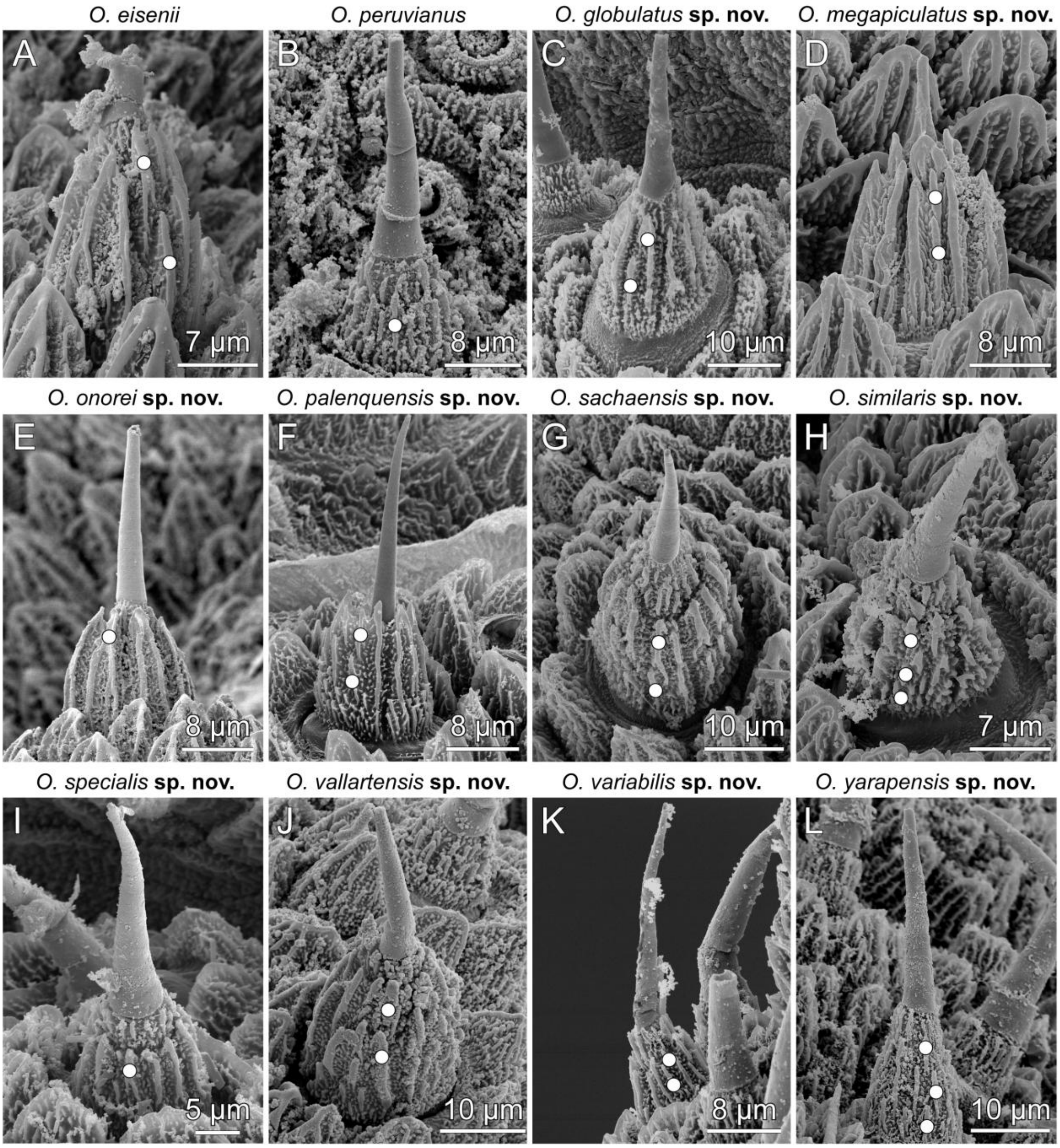

Figure S23. Type I antennal sensillae in the species examined, Related to Results. Scanning electron micrographs. White dots indicate scale ranks on the sensilla base. (A) *O. eisenii*. (B) *O. peruvianus*. (C) *O. globulatus* **sp. nov**. (D) *O. megapiculatus* **sp. nov**. (E) *O. onorei* **sp. nov**. (F) *O. palenquensis* **sp. nov**. (G) *O. sachaensis* **sp. nov**. (H) *O. similaris* **sp. nov**. (I) *O. specialis* **sp. nov**. (J) *O. vallartensis* **sp. nov**. (K) *O. variabilis* **sp. nov**. (L) *O. yarapensis* **sp. nov**.

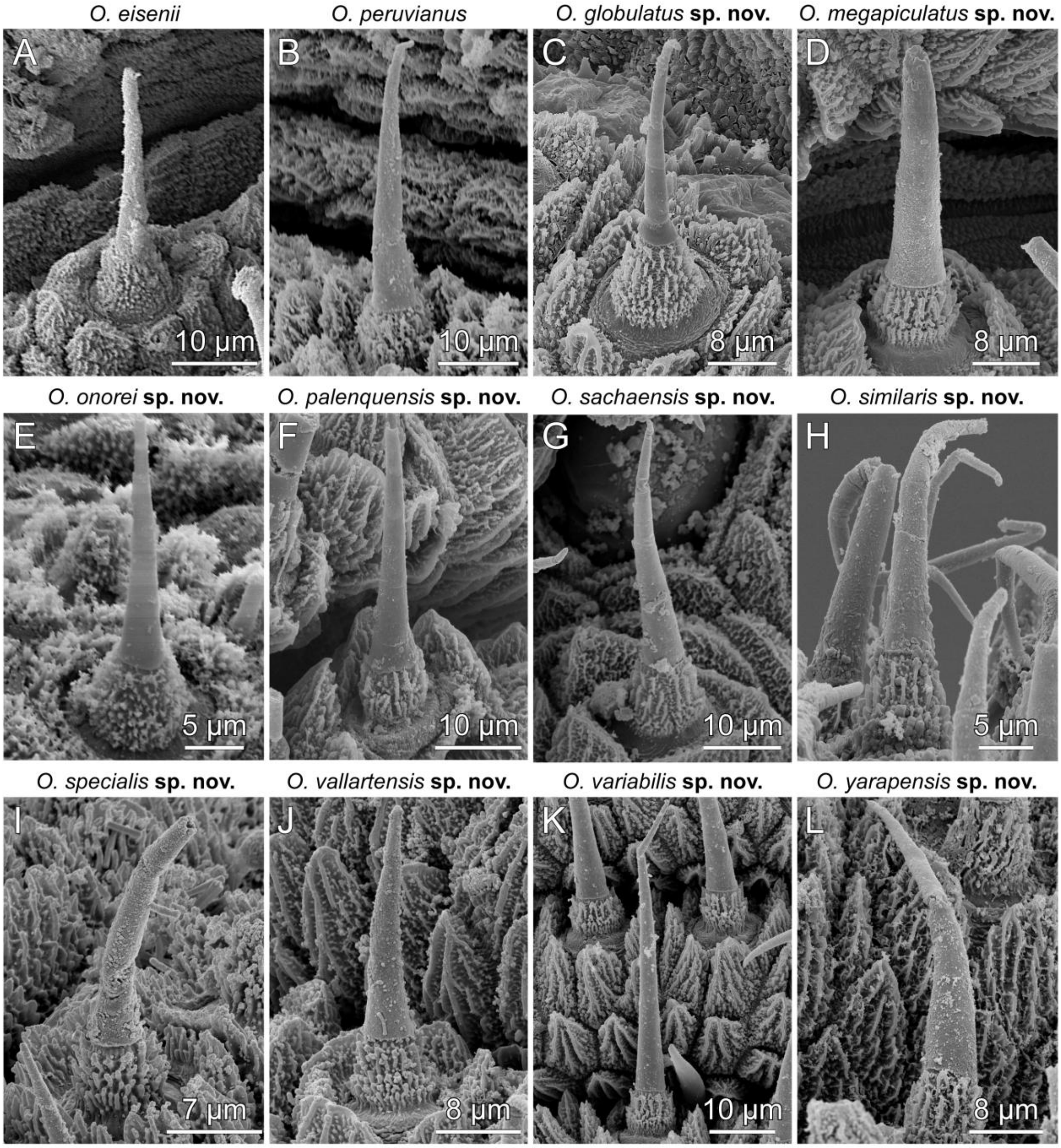

Figure S24. Type II antennal sensillae in the species examined, Related to Results. Scanning electron micrographs. (A) *O. eisenii*. (B) *O. peruvianus*. (C) *O. globulatus* **sp. nov**. (D) *O. megapiculatus* **sp. nov**. (E) *O. onorei* **sp. nov**. (F) *O. palenquensis* **sp. nov**. (G) *O. sachaensis* **sp. nov**. (H) *O. similaris* **sp. nov**. (I) *O. specialis* **sp. nov**. (J) *O. vallartensis* **sp. nov**. (K) *O. variabilis* **sp. nov**. (L) *O. yarapensis* **sp. nov**.

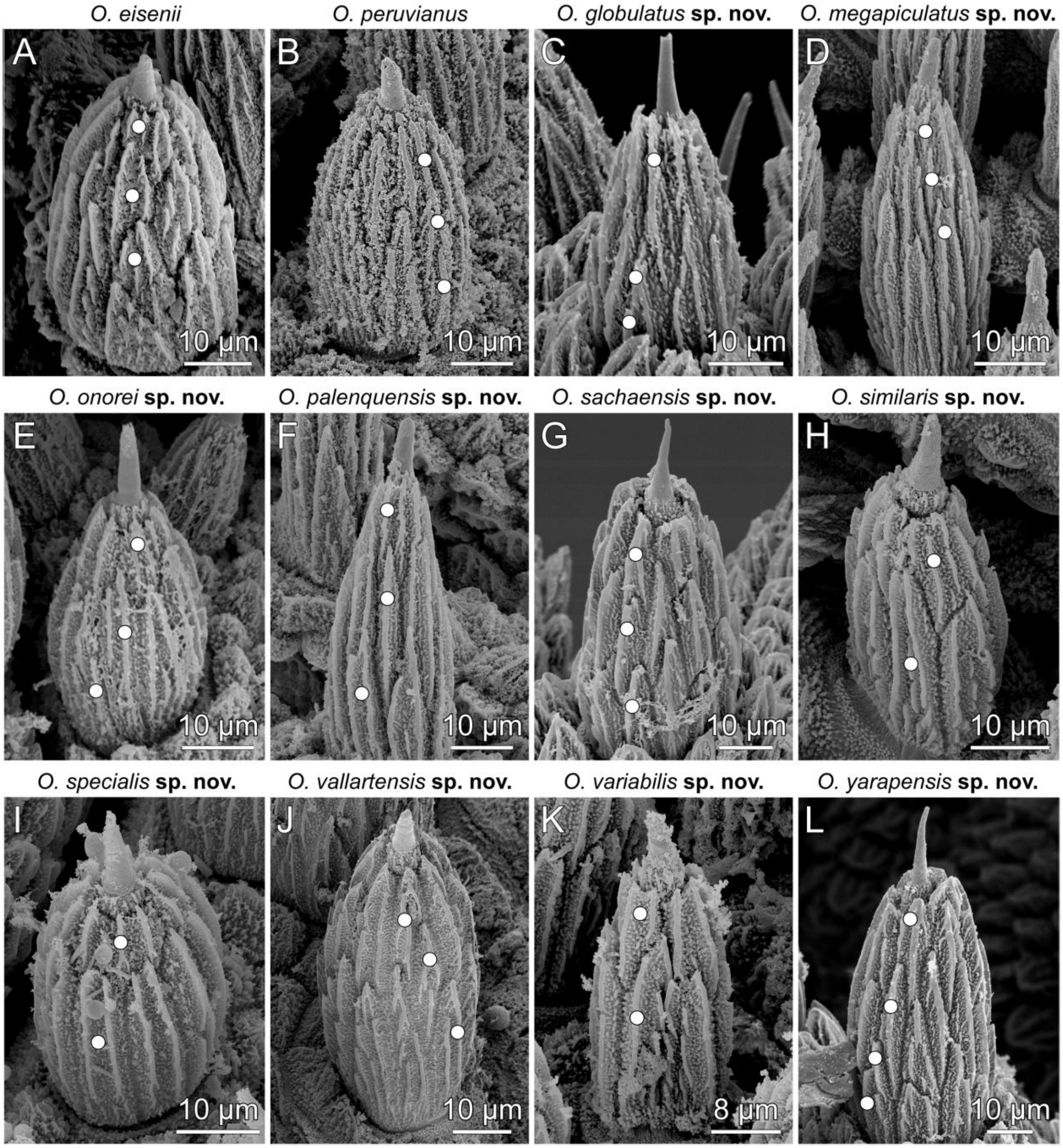

Figure S25. Spindle-shaped sensillae in the species examined, Related to Results. Scanning electron micrographs. White dots mark the scale ranks on spindle-shaped sensilla base. (A) *O. eisenii*. (B) *O. peruvianus*. (C) *O. globulatus* **sp. nov**. (D) *O. megapiculatus* **sp. nov**. (E) *O. onorei* **sp. nov**. (F) *O. palenquensis* **sp. nov**. (G) *O. sachaensis* **sp. nov**. (H) *O. similaris* **sp. nov**. (I) *O. specialis* **sp. nov**. (J) *O. vallartensis* **sp. nov**. (K) *O. variabilis* **sp. nov**. (L) *O. yarapensis* **sp. nov**.

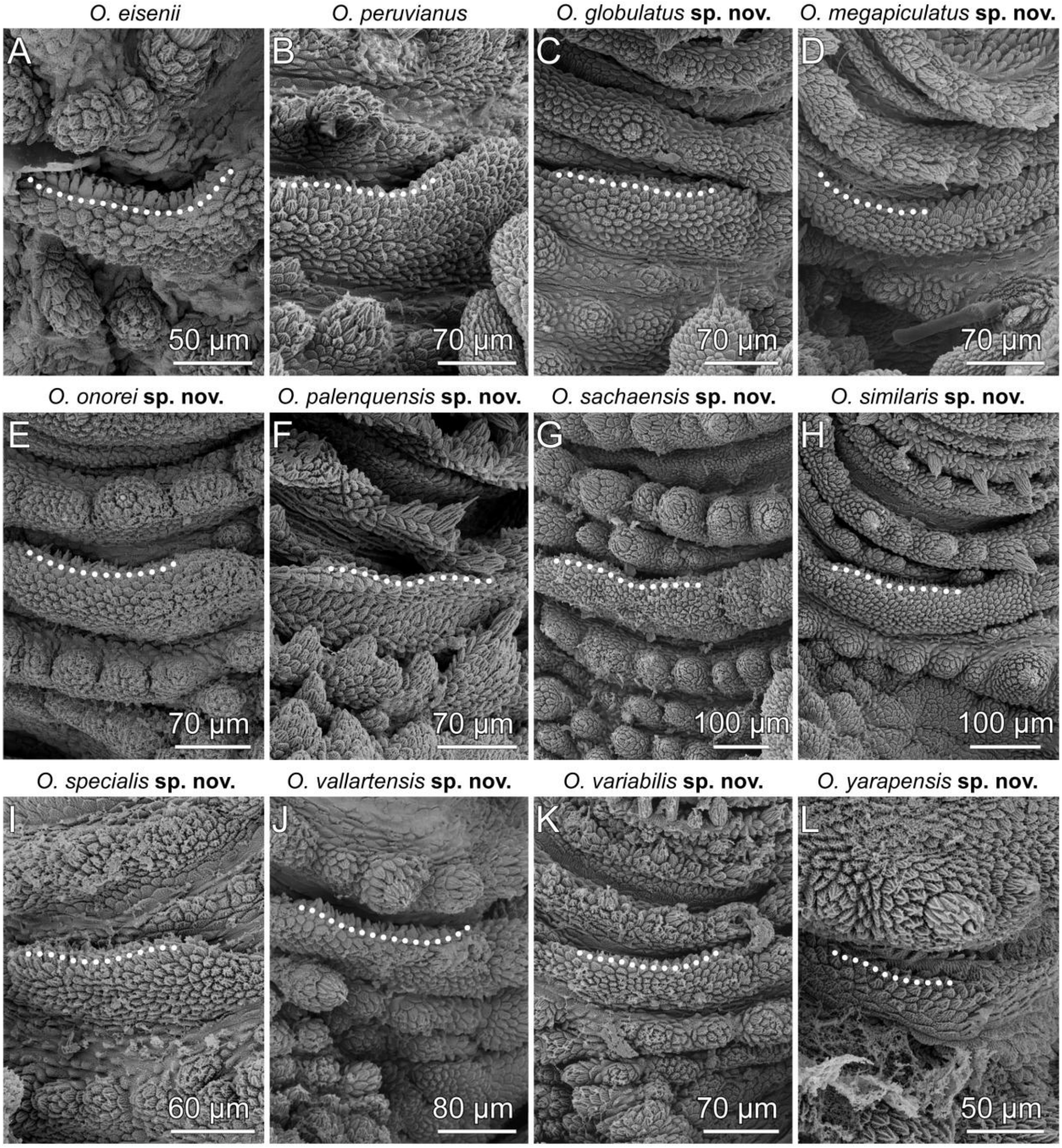

Figure S26. Frontal organs in the species examined, Related to Results. Scanning electron micrographs. Dotted lines mark the delimitation between outer and inner surface of the frontal organ. (A) *O. eisenii*. (B) *O. peruvianus*. (C) *O. globulatus* **sp. nov**. (D) *O. megapiculatus* **sp. nov**. (E) *O. onorei* **sp. nov**. (F) *O. palenquensis* **sp. nov**. (G) *O. sachaensis* **sp. nov**. (H) *O. similaris* **sp. nov**. (I) *O. specialis* **sp. nov**. (J) *O. vallartensis* **sp. nov**. (K) *O. variabilis* **sp. nov**. (L) *O. yarapensis* **sp. nov**.

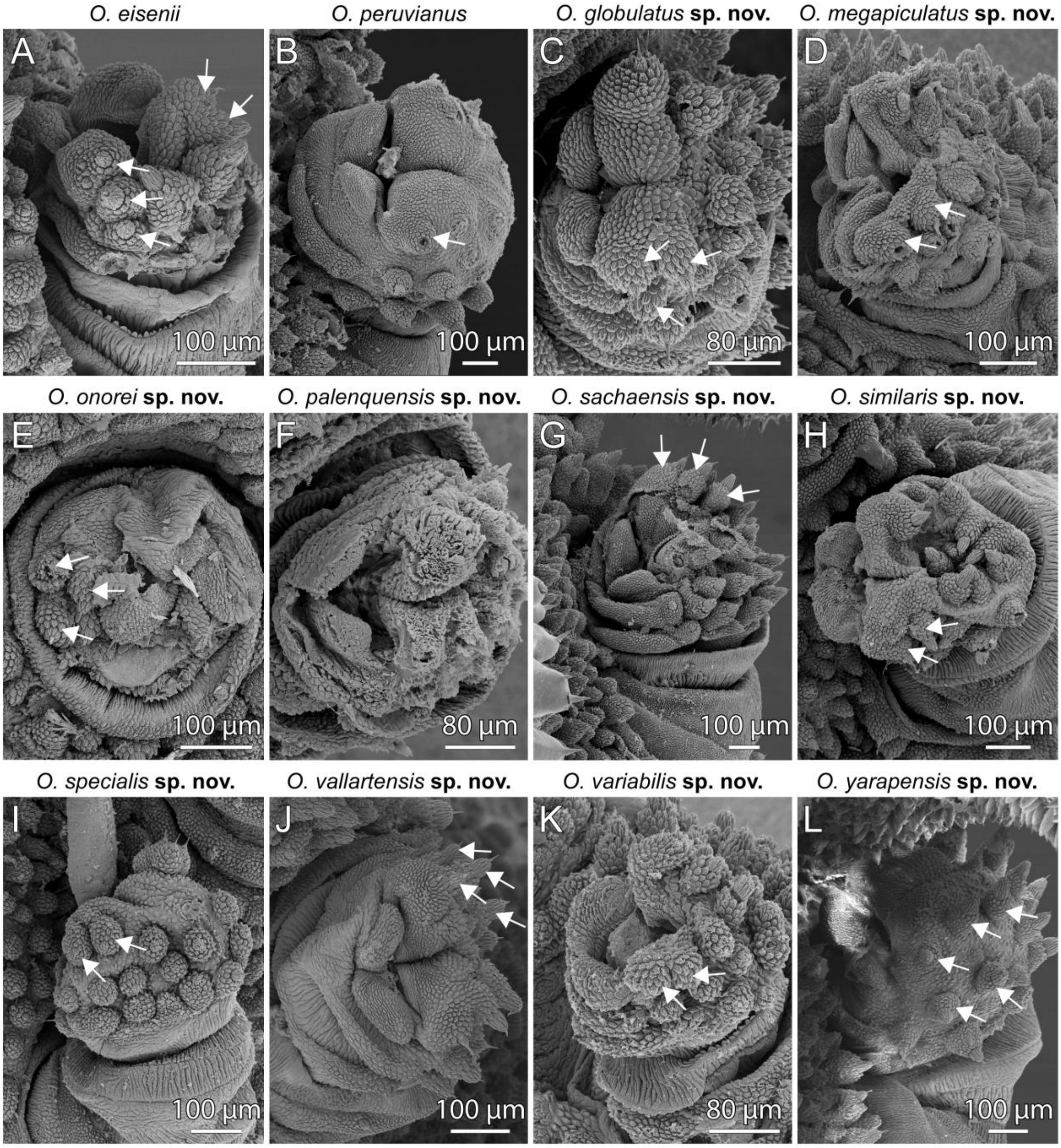

Figure S27. Slime papillae in the species examined, Related to Results. Scanning electron micrographs. Arrows point to dermal papillae on the slime papillae. (A) *O. eisenii*. (B) *O. peruvianus*. (C) *O. globulatus* **sp. nov**. (D) *O. megapiculatus* **sp. nov**. (E) *O. onorei* **sp. nov**. (F) *O. palenquensis* **sp. nov**. (G) *O. sachaensis* **sp. nov**. (H) *O. similaris* **sp. nov**. (I) *O. specialis* **sp. nov**. (J) *O. vallartensis* **sp. nov**. (K) *O. variabilis* **sp. nov**. (L) *O. yarapensis* **sp. nov**.

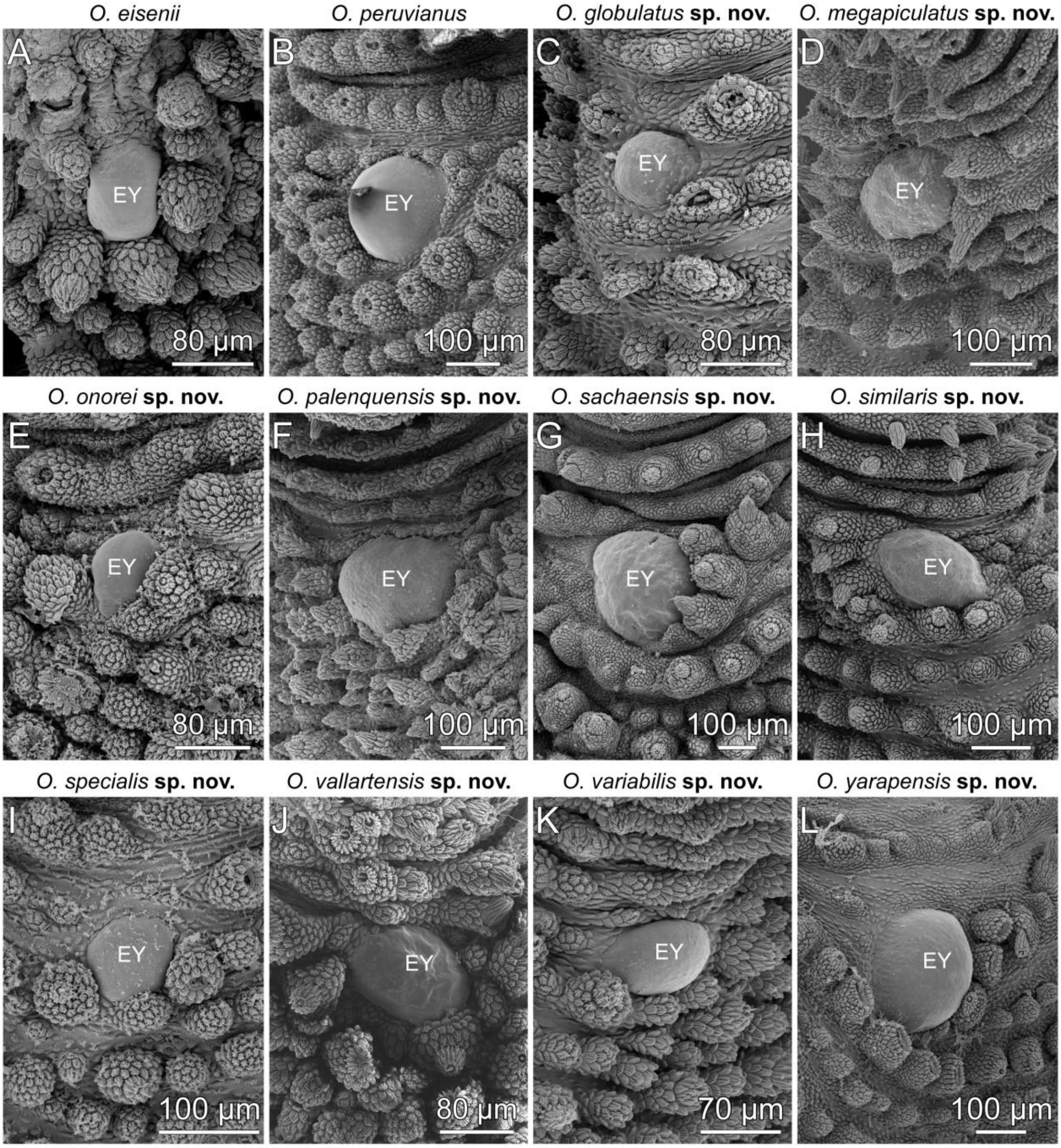

Figure S28. The eyes in the species examined, Related to Results. Scanning electron micrographs. (A) *O. eisenii*. (B) *O. peruvianus*. (C) *O. globulatus* **sp. nov**. (D) *O. megapiculatus* **sp. nov**. (E) *O. onorei* **sp. nov**. (F) *O. palenquensis* **sp. nov**. (G) *O. sachaensis* **sp. nov**. (H) *O. similaris* **sp. nov**. (I) *O. specialis* **sp. nov**. (J) *O. vallartensis* **sp. nov**. (K) *O. variabilis* **sp. nov**. (L) *O. yarapensis* **sp. nov**. Abbreviation: EY, eye.

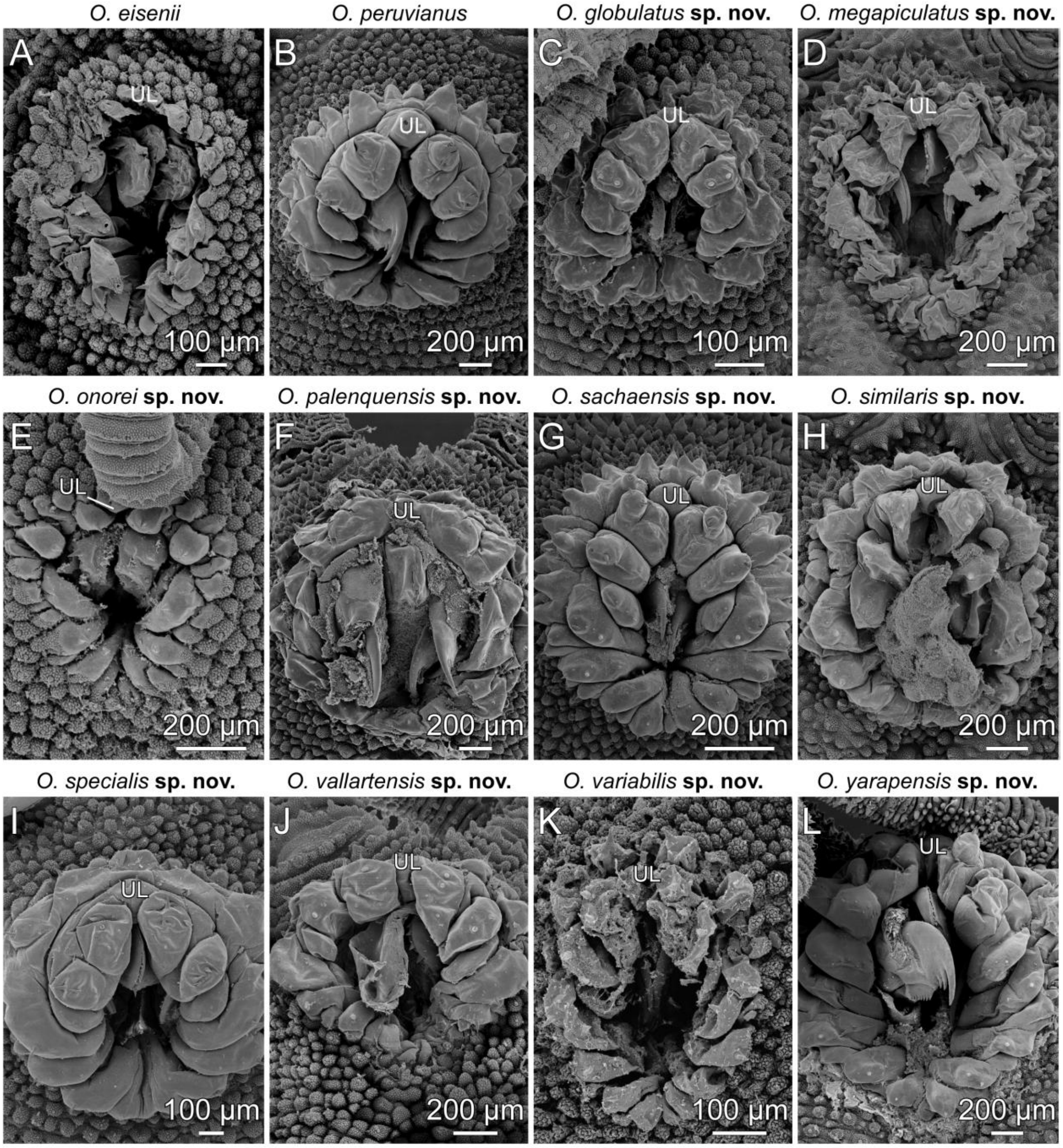

Figure S29. The mouth in the species examined, Related to Results. Scanning electron micrographs. (A) *O. eisenii*. (B) *O. peruvianus*. (C) *O. globulatus* **sp. nov**. (D) *O. megapiculatus* **sp. nov**. (E) *O. onorei* **sp. nov**. (F) *O. palenquensis* **sp. nov**. (G) *O. sachaensis* **sp. nov**. (H) *O. similaris* **sp. nov**. (I) *O. specialis* **sp. nov**. (J) *O. vallartensis* **sp. nov**. (K) *O. variabilis* **sp. nov**. (L) *O. yarapensis* **sp. nov**. Abbreviation: ULl, unpaired anterior-most lip.

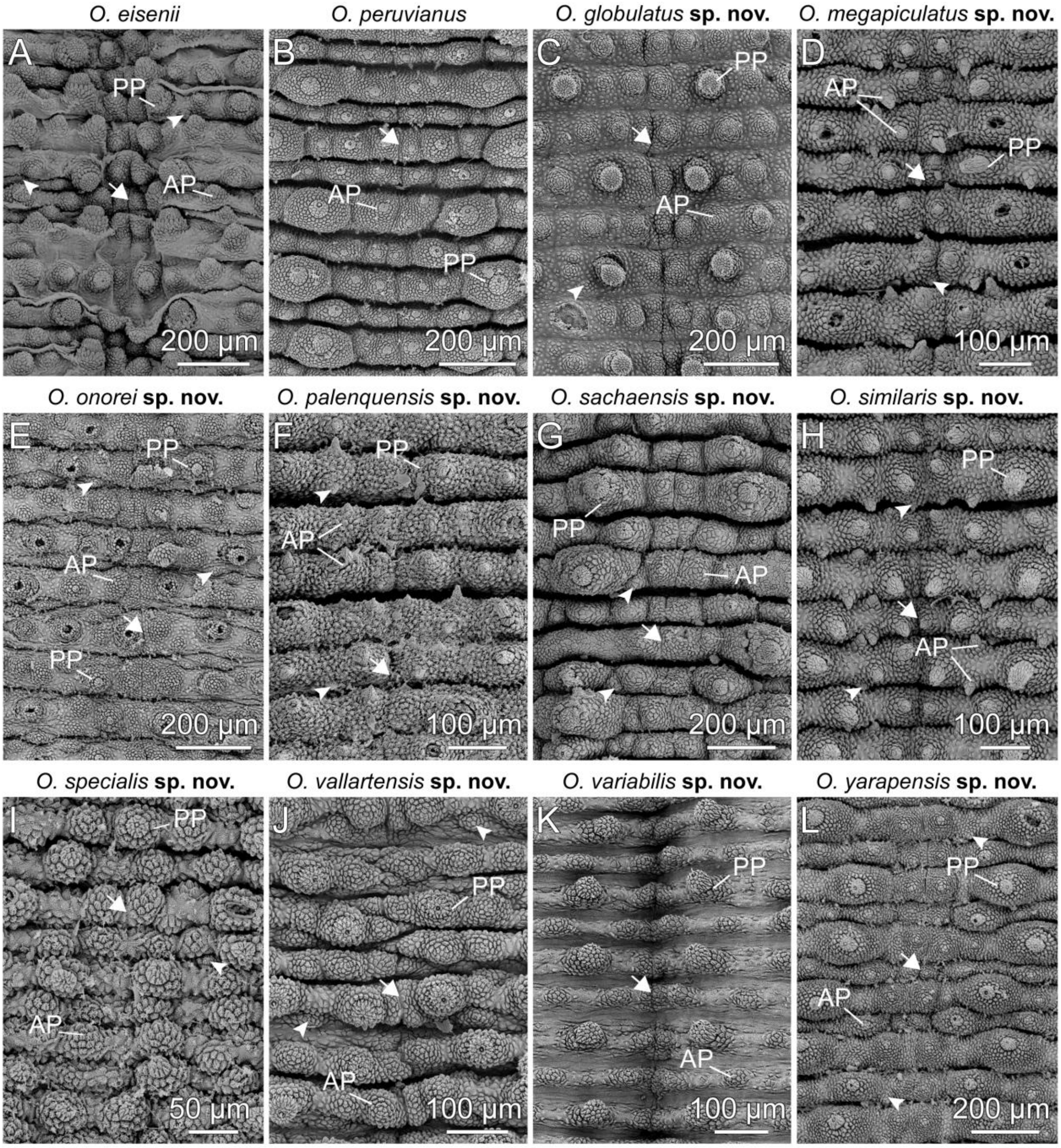

Figure S30. Dorsal integument in the species examined, Related to Results. Scanning electron micrographs. Arrows indicate the dorsomedian furrow; arrowheads point to peripheral accessory papillae. (A) *O. eisenii*. (B) *O. peruvianus*. (C) *O. globulatus* **sp. nov**. (D) *O. megapiculatus* **sp. nov**. (E) *O. onorei* **sp. nov**. (F) *O. palenquensis* **sp. nov**. (G) *O. sachaensis* **sp. nov**. (H) *O. similaris* **sp. nov**. (I) *O. specialis* **sp. nov**. (J) *O. vallartensis* **sp. nov**. (K) *O. variabilis* **sp. nov**. (L) *O. yarapensis* **sp. nov**. Abbreviations: AP, accessory papilla; PP, primary papilla.

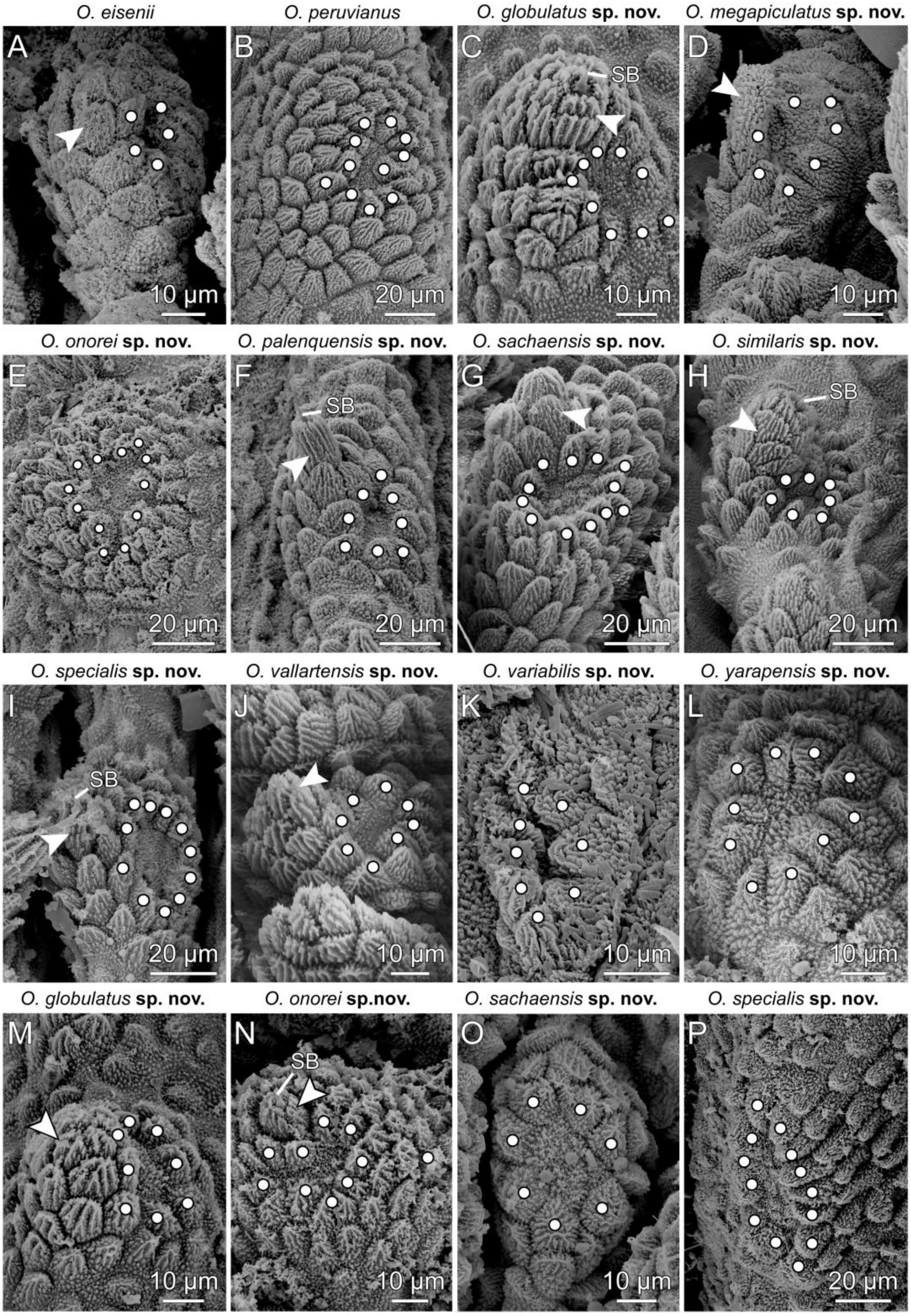

Figure S31. Type I crater-shaped papillae in the species examined, Related to Results. Scanning electron micrographs. White dots indicate the number of scales in the apical collar. Arrowheads point to apical pieces. (A) *O. eisenii*. (B) *O. peruvianus*. (C) *O. globulatus* **sp. nov**. (D) *O. megapiculatus* **sp. nov**. (E) *O. onorei* **sp. nov**. (F) *O. palenquensis* **sp. nov**. (G) *O. sachaensis* **sp. nov**. (H) *O. similaris* **sp. nov**. (I) *O. specialis* **sp. nov**. (J) *O. vallartensis* **sp. nov**. (K) *O. variabilis* **sp. nov**. (L) *O. yarapensis* **sp. nov**. (M–P) Morphological variations of type I crater-shaped papillae in *O. globulatus* **sp. nov.** (M), *O. onorei* **sp. nov.** (N), *O. sachaensis* **sp. nov.** (O), and *O. specialis* **sp. nov.** (P). Abbreviation: SB, sensory bristle.

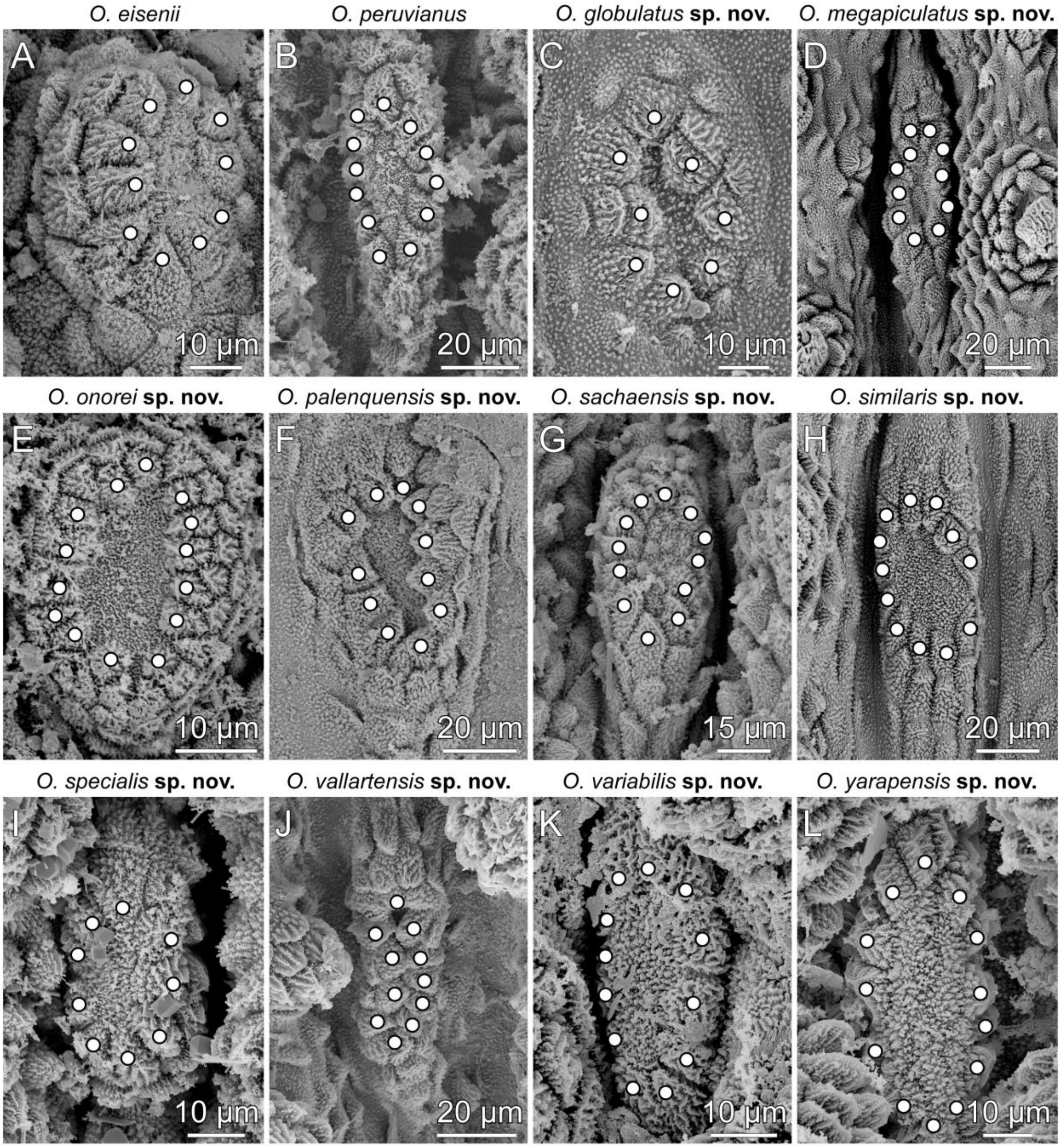

Figure S32. Type II crater-shaped papillae in the species examined, Related to Results. Scanning electron micrographs. White dots mark the number of scales in the apical collar. (A) *O. eisenii*. (B) *O. peruvianus*. (C) *O. globulatus* **sp. nov**. (D) *O. megapiculatus* **sp. nov**. (E) *O. onorei* **sp. nov**. (F) *O. palenquensis* **sp. nov**. (G) *O. sachaensis* **sp. nov**. (H) *O. similaris* **sp. nov**. (I) *O. specialis* **sp. nov**. (J) *O. vallartensis* **sp. nov**. (K) *O. variabilis* **sp. nov**. (L) *O. yarapensis* **sp. nov**.

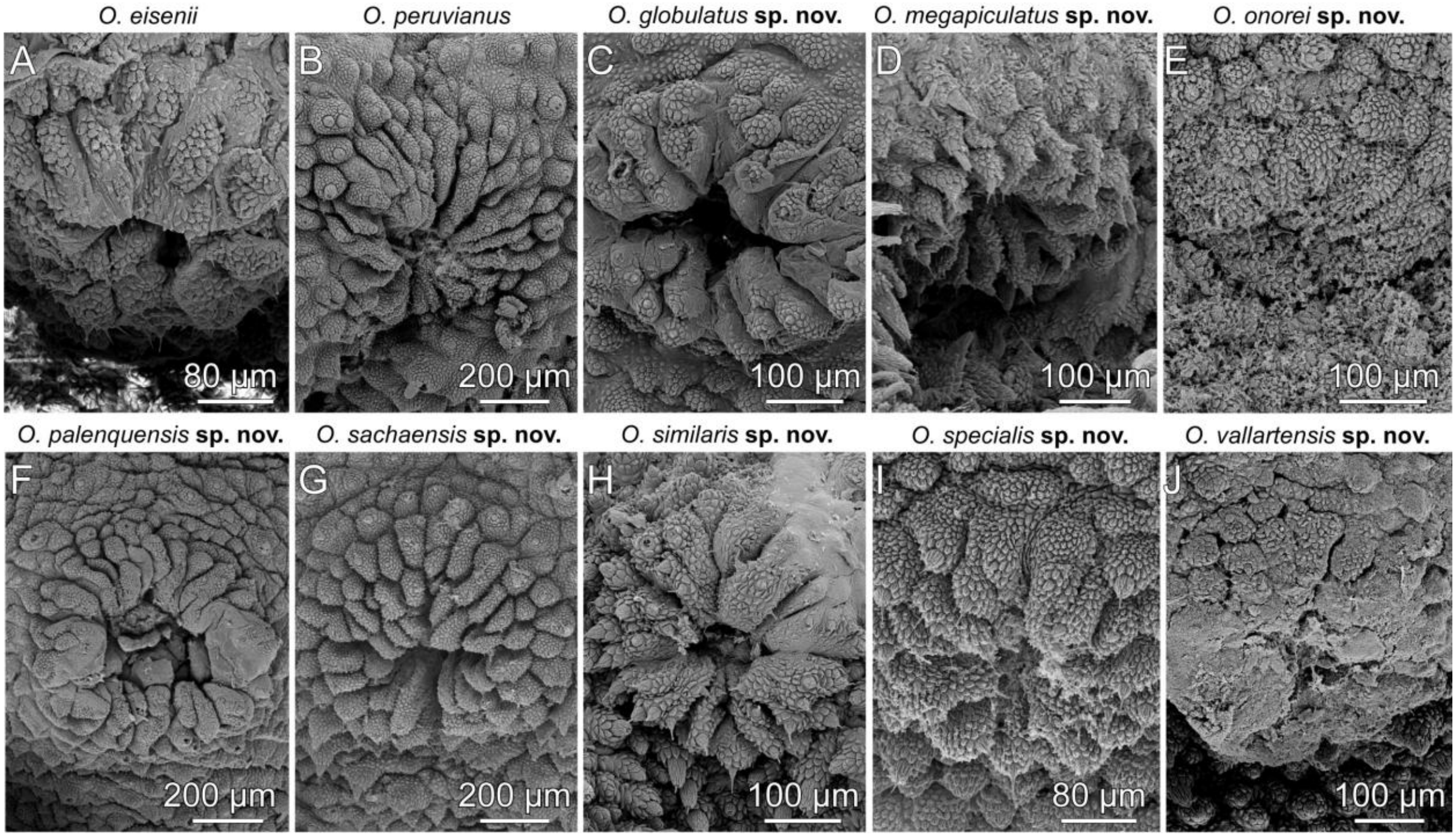

Figure S33. Female genital pad in the species examined, Related to Results. Scanning electron micrographs. (A) *O. eisenii*. (B) *O. peruvianus*. (C) *O. globulatus* **sp. nov**. (D) *O. megapiculatus* **sp. nov**. (E) *O. onorei* **sp. nov**. (F) *O. palenquensis* **sp. nov**. (G) *O. sachaensis* **sp. nov**. (H) *O. similaris* **sp. nov**. (I) *O. specialis* **sp. nov**. (J) *O. vallartensis* **sp. nov**.

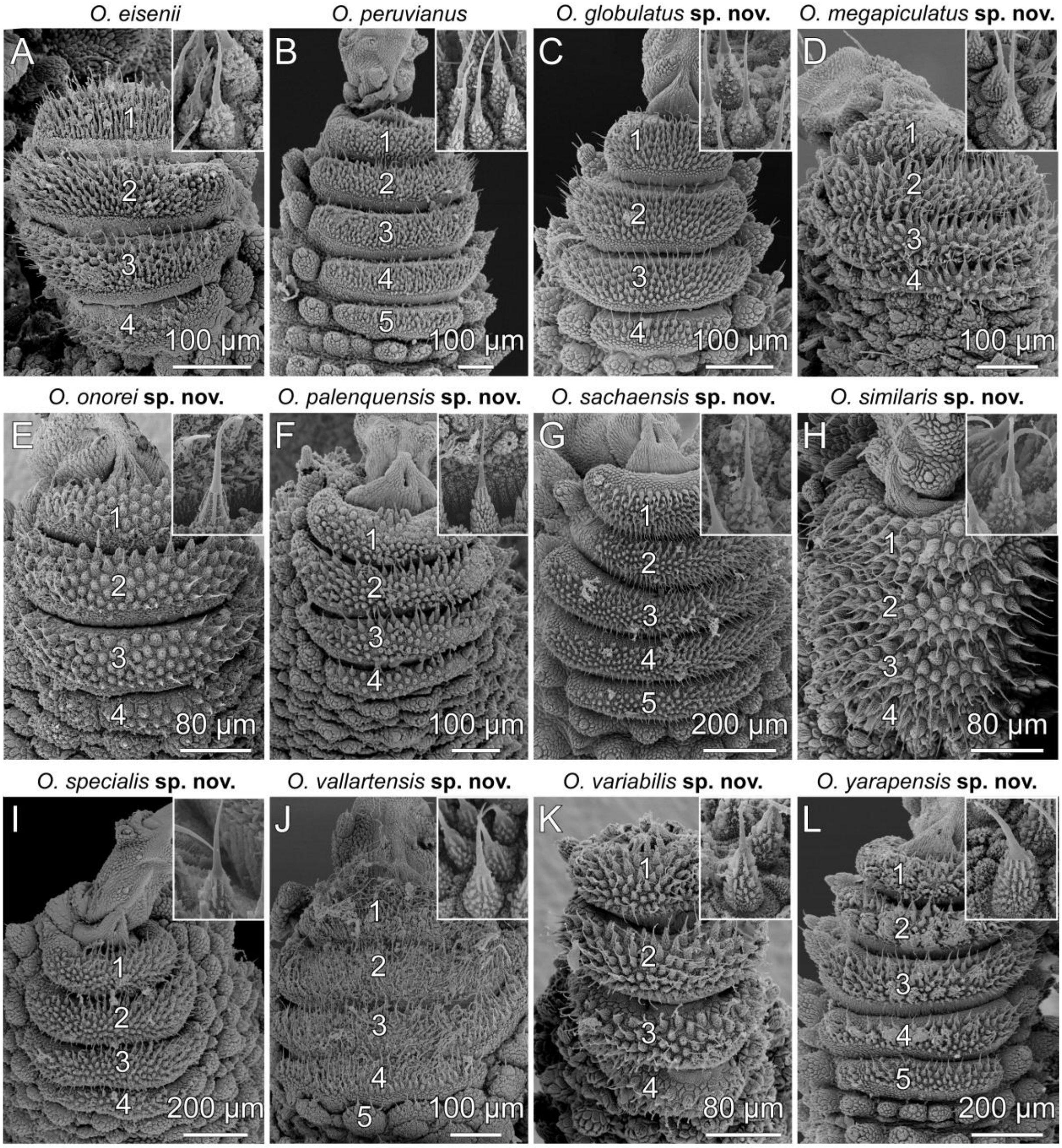

Figure S34. **Spinous pads in the species examined, Related to Results.** Scanning electron micrographs. Spinous pads are numbered in the proximal direction. Insets show details of a bristle from the spinous pad. (A) *O. eisenii*. (B) *O. peruvianus*. (C) *O. globulatus* **sp. nov**. (D) *O. megapiculatus* **sp. nov**. (E) *O. onorei* **sp. nov**. (F) *O. palenquensis* **sp. nov**. (G) *O. sachaensis* **sp. nov**. (H) *O. similaris* **sp. nov**. (I) *O. specialis* **sp. nov**. (J) *O. vallartensis* **sp. nov**. (K) *O. variabilis* **sp. nov**. (L) *O. yarapensis* **sp. nov**.

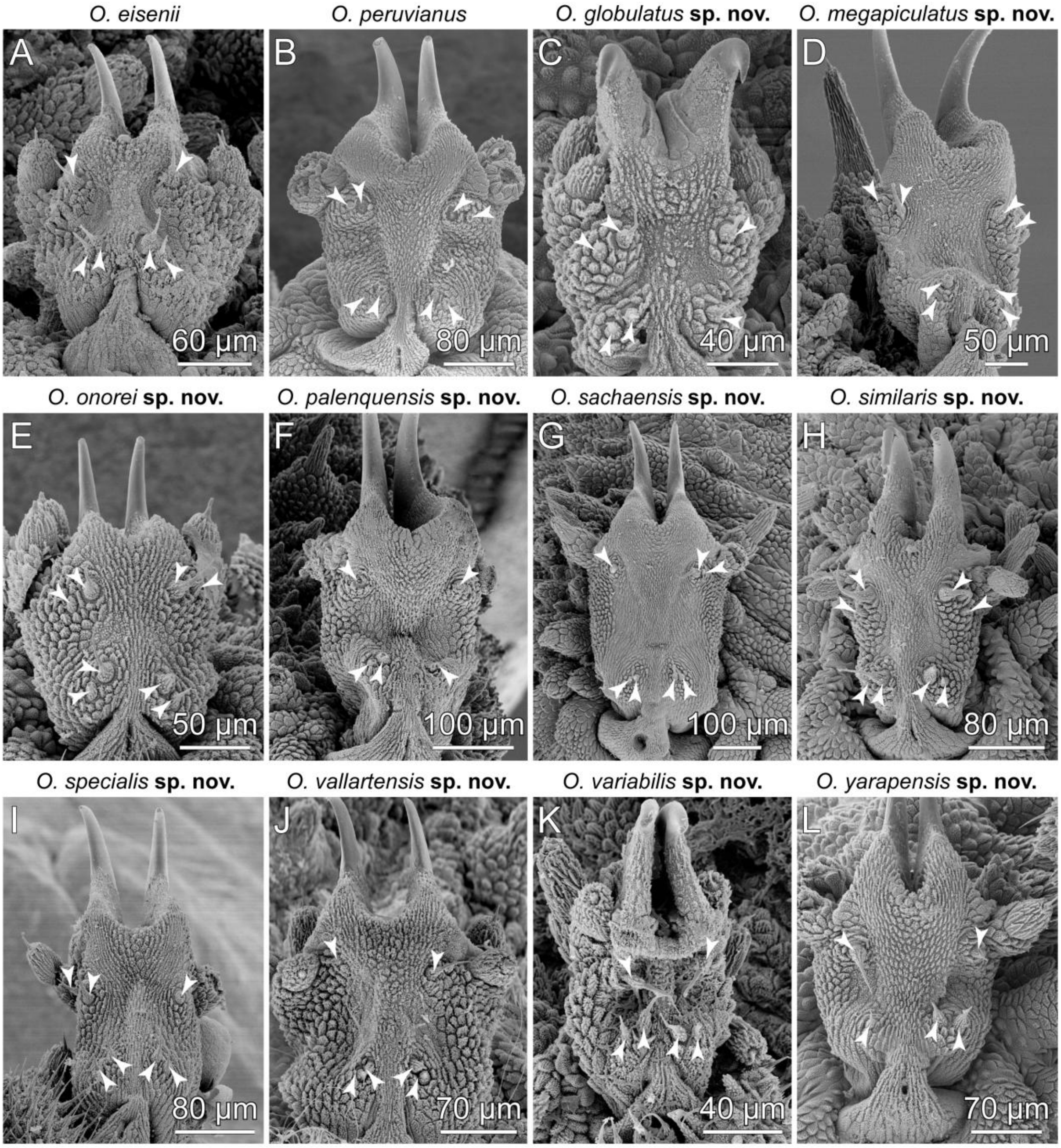

Figure S35. Foot and setiform ridges pads in the species examined, Related to Results. Scanning electron micrographs. Arrowheads point to bristles on proximal and distal setiform ridges. (A) *O. eisenii*. (B) *O. peruvianus*. (C) *O. globulatus* **sp. nov**. (D) *O. megapiculatus* **sp. nov**. (E) *O. onorei* **sp. nov**. (F) *O. palenquensis* **sp. nov**. (G) *O. sachaensis* **sp. nov**. (H) *O. similaris* **sp. nov**. (I) *O. specialis* **sp. nov**. (J) *O. vallartensis* **sp. nov**. (K) *O. variabilis* **sp. nov**. (L) *O. yarapensis* **sp. nov**.

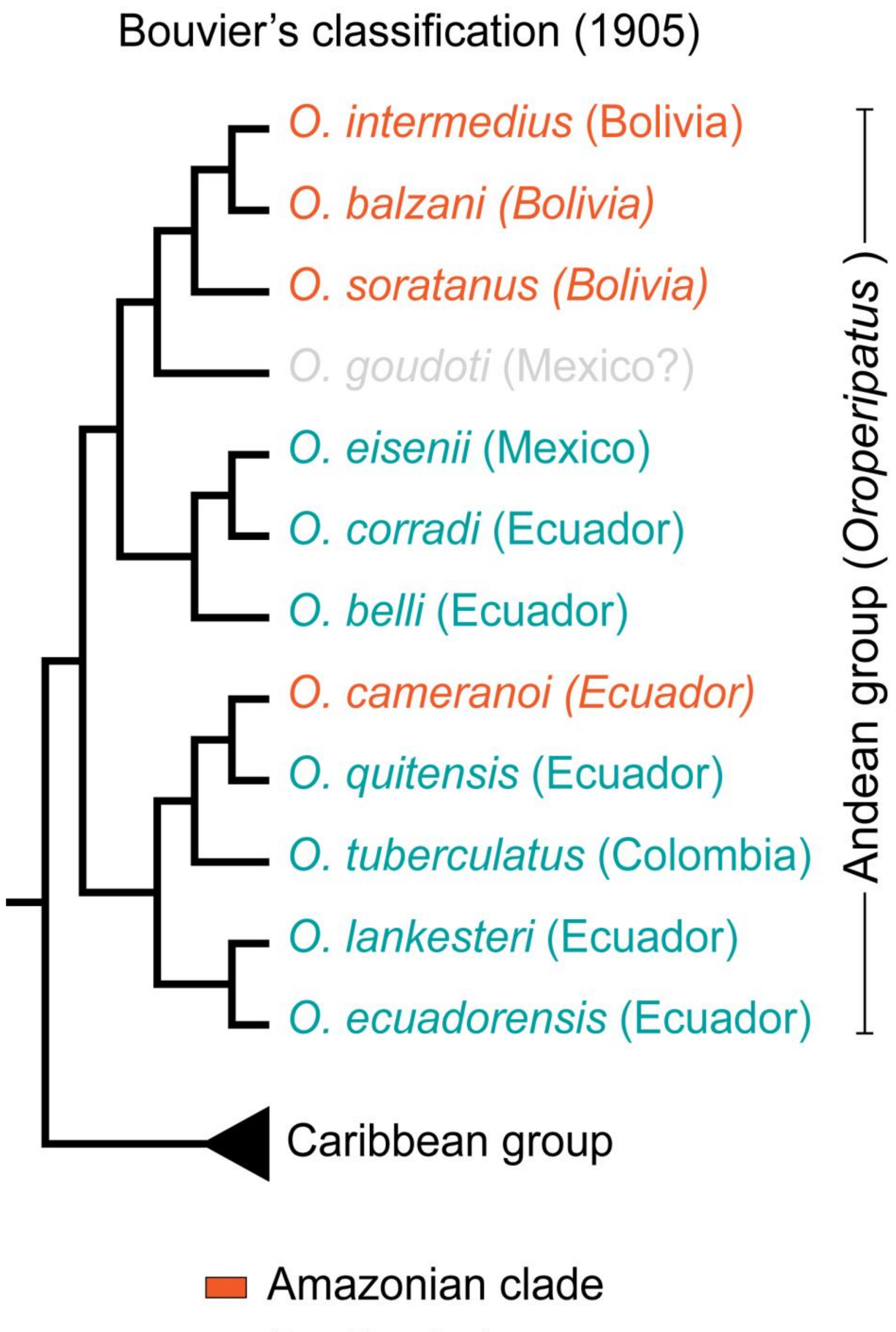

Figure S36. Morphology-based grouping outlined by Bouvier[5] within *Oroperipatus*, Related to Discussion. Colours indicate the putative phylogenetic position of species within the Amazonian (orange) and Pacific (blue) subclades of *Oroperipatus* (this study), as inferred from their geographic distributions. Note that *O. goudoti* (grey) is a *nomen dubium* and its occurrence in Mexico is questionable[1].

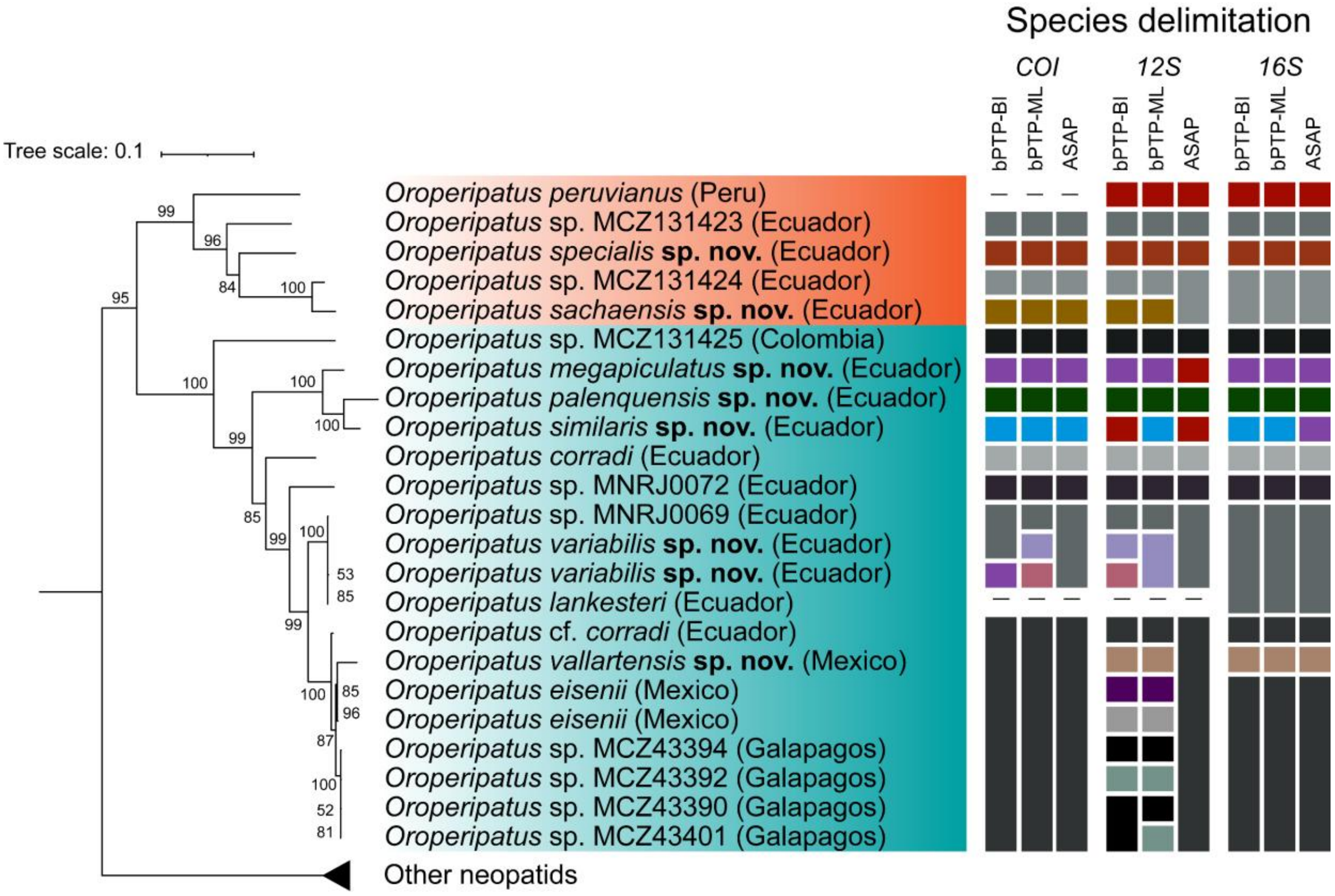

Figure S37. Single locus species delimitation analysis within *Oroperipatus*, Related to Results. Bayesian (bPTP-BI) and maximum likelihood (bPTP-ML) bPTP and ASAP species delimitation across three loci (*12S rRNA*, *16S rRNA*, *COI*) and mapped onto the maximum likelihood topology inferred from concatenated nucleotide sequence data. Numbers indicate bootstrap support values. Amazonian (orange) and Pacific (blue) subclades are highlighted. Colours correspond to individual species.

SUPPLEMENTAL TABLES

Table S1. Data of examined specimens, Related to STAR Methods.

Species	Locality	Date	Collector	# of legs	Sex	Length (mm)	Width (mm)	Height (mm)	Collection ID	Status
O. eisenii	Guadalajara, Colonia del Sur, Nicaragua 2036, Mexico	V.2007	M.E. González Hernandez	28	F	33,1	0,32	0,2	[1]ZMH-ARA-A00320101	Topotype
O. peruvianus	Tabaconas Namballe, San Ignacio, Cajamarca,Perú	2009	Maik Dobiey	35	F	53,1	0,57	0,43	[1]ZMH-ARA-A0032094	Topotype
O. peruvianus	Tabaconas Namballe, San Ignacio, Cajamarca,Perú	2009	Maik Dobiey	35	F	36,2	0,54	0,42	[1]ZMH-ARA-A0032095	Topotype
O. peruvianus	Tabaconas Namballe, San Ignacio, Cajamarca,Perú	2009	Maik Dobiey	35	F	39,1	0,74	0,35	[1]ZMH-ARA-A0032096	Topotype
O. peruvianus	Tabaconas Namballe, San Ignacio, Cajamarca,Perú	2009	Maik Dobiey	29	M	31,5	0,52	0,34	[1]ZMH-ARA-A0032097	Topotype
O. globulatus **sp. nov.**	Mazán Valley, Cuenca, Azuay, Ecuador	1986	V. M. St. J. Read	24	F	40,2	0,38	0,31	[2]QCAZI–282080	Holotype
O. globulatus **sp. nov.**	Mazán Valley, Cuenca, Azuay, Ecuador	1986	V. M. St. J. Read	24	F	39,3	0,4	0,31	[1]ZMH-ARA-A00320100	Paratype
O. megapiculatus **sp. nov.**	Los Ríos, Santo Domingo, Ecuador	V.2022	A. Tartara, F. Villagomez	27	F	29,7	0,55	0,26	[2]QCAZI–282081	Holotype
O. onorei **sp. nov.**	Aluriquín, Pichincha, Ecuador	V.1985	G. Onore	27	F	29,6	0,33	0,25	[2]QCAZI–282082	Holotype
O. onorei **sp. nov.**	Aluriquín, Pichincha, Ecuador	V.1985	G. Onore	29	F	45,5	0,67	0,34	[1]ZMH-ARA-A0032093	Paratype
O. palenquensis **sp. nov.**	Río Palenque, Los Ríos, Santo Domingo, Ecuador	VII.1977	Ken Miyata	28	F	31,3	0,46	0,33	[2]QCAZI–282083	Holotype
O. palenquensis **sp. nov.**	Río Palenque, Los Ríos, Santo Domingo, Ecuador	VII.1977	Ken Miyata	27	F	19,5	0,18	0,18	[1]ZMH-ARA-A0032090	Paratype
O. palenquensis **sp. nov.**	Río Palenque, Los Ríos, Santo Domingo, Ecuador	VII.1977	Ken Miyata	27	F	13,8	0,18	0,12	[1]ZMH-ARA-A0032091	Paratype
O. palenquensis **sp. nov.**	Río Palenque, Los Ríos, Santo Domingo, Ecuador	1980	R. Castello	27	F	27,6	0,3	0,15	[1]ZMH-ARA-A0032092	Paratype
O. sachaensis **sp. nov.**	Joya de las Sachas, Napo, Ecuador	VI.1983	J. Boos	37	F	59,8	0,57	0,44	[2]QCAZI–282084	Holotype
O. similaris **sp. nov.**	Los Ríos, Santo Domingo, Ecuador	VI.2022	A. Tartara, F. Villagomez	27	F	28,4	0,54	0,36	[2]QCAZI–282085	Holotype
O. specialis **sp. nov.**	San Rafael, Napo, Ecuador	I.1985	G. Onore	24	M	12,3	0,3	0,2	[2]QCAZI–282086	Holotype
O. specialis **sp. nov.**	San Rafael, Napo, Ecuador	I.1985	G. Onore	26	F	13,3	0,32	0,27	[1]ZMH-ARA-A0032088	Paratype
O. specialis **sp. nov.**	San Rafael, Napo, Ecuador	I.1985	G. Onore	26	F	23,6	0,47	0,24	[1]ZMH-ARA-A0032089	Paratype
O. vallartensis **sp. nov.**	Puerto Vallarta, Jalisco, Mexico	VII.2012	R. García de Quevedo	27	F	32,3	0,36	0,34	[3]CNAC/Ony00086	Holotype
O. vallartensis **sp. nov.**	Puerto Vallarta, Jalisco, Mexico	V.2013	F. Cupul-Magaña	27	F	25,1	0,25	0,21	[1]ZMH-ARA-A0032098	Paratype
O. vallartensis **sp. nov.**	Puerto Vallarta, Jalisco, Mexico	VI.2013	P. Medina	28	F	58	0,42	0,35	[1]ZMH-ARA-A0032099	Paratype
O. variabilis **sp. nov.**	Los Ríos, Santo Domingo, Ecuador	IV.2022	A. Tartara, F. Villagomez	26	F	16,1	0,3	0,2	[2]QCAZI–282087	Holotype
O. variabilis **sp. nov.**	Los Ríos, Santo Domingo, Ecuador	VI.2022	A. Tartara, F. Villagomez	26	F	0,99	0,13	0,11	[1]ZMH-ARA-A00320102	Paratype
O. variabilis **sp. nov.**	Los Ríos, Santo Domingo, Ecuador	VI.2022	A. Tartara, F. Villagomez	26	F	0,9	0,16	0,13	[1]ZMH-ARA-A00320103	Paratype
O. yarapensis **sp. nov.**	Low Yarapa River, Loreto, Perú	XI.1993	W. Lamar	37	M	40,9	0,58	0,33	[4]MZUCR76	Holotype

[1]Aracnology collection of the Museum of Nature Hamburg, Leibniz Institute for the Analysis of Biodiversity Change, Hamburg, Germany. [2]Museo de Zoología de la Universidad Católica de Ecuador, Quito, Ecuador. [3]Onychophora collection associated to the Colección Nacional de Ácaros, Instituto de Biología Universidad Nacional Autónoma de México, Mexico City, Mexico. [4]Museo de Zoología de la Universidad de Costa Rica, San José, Costa Rica.

Table S2. Molecular data used for phylogenetic analyses with corresponding GenBank accession numbers, Related to STAR Methods.

Species	Voucher	Origin	*COI*	*12S rRNA*	*16S rRNA*	*18S rRNA*
**Peripatopsidae**						
Kumbadjena occidentalis	MCZ131369	Australia	KC754653	KC754484	KC754535	MG973616
Leucopatus anophthalmus	MCZ131360	Tasmania	KC754691	KC754517	KC754572	MG973639
Metaperipatus blainvillei	MCZ131335	Chile	KC754658	KC754489	KC754540	MG973649
Occiperipatoides gilesii	MCZ131370	Australia	KC754660	KC754491	KC754542	MG973614
Ooperipatellus cryptus	MCZ131362	Tasmania	HQ655618	KC754492	KC754544	MG973628
Peripatoides suteri	MCZ131358	New Zealand	KC754683	KC754511	KC754564	MG973637
Peripatopsis lawrencei	MCZ131348	South Africa	KC754687	KC754514	KC754568	MG973647
Peripatopsis moseleyi	MCZ131346	South Africa	KC754688	KC754515	KC754569	KC754595
Tasmanipatus barretti	MCZ131363	Tasmania	KC754692	KC754518	KC754573	MG973640
**Peripatidae**						
Eoperipatus cf. *horsti*	MCZ131341	Thailand	KC754636	KC754471	KC754519	MG973605
Epiperipatus acacioi	MNRJ0044	Brazil	HQ655618	HQ404920	MG973517	MG973554
Epiperipatus beckeri	MNRJ0045	Brazil	MN905627	MN639356	MN544103	MN705438
Epiperipatus biolleyi	MZUSP0012	Costa Rica	MN905667	MN639389	MN544142	MG973570
Epiperipatus edwardsii	GF180312HC00306	French Guiana	HG531958	HG531961	HG531962	HG531959
Epiperipatus edwardsii	IZ–141306	French Guiana	MN905666	MN933780	HG531962	MG973542
Epiperipatus hyperbolicus	MNRJ0105	Brazil	MN905654	MN639380	MN544130	MN705448
Epiperipatus lucerna	MNRJ0102	Brazil	MN905653	MN639379	MN544129	MN705447
Epiperipatus marajoara	MZUSP0022	Brazil	MN905681	MN639404	MN544157	MN705462
Epiperipatus marajoara	MZUSP0023	Brazil	MN905682	MN639405	MN544158	MN705463
Epiperipatus paurognostus	MNRJ0045	Brazil	MH107346	MG973696	MG973516	MG973553
Epiperipatus sp.	UNACHI	Panama	MH107365	MG973685	MG973491	MG973578
Epiperipatus sp.	MCZ131428	Colombia	MH107352	MG973706	MG973525	MG973590
Epiperipatus sp.	MCZ131430	Colombia	MH107354	MG973708	MG973527	MG973592
Epiperipatus sp.	MZUSP0111	Panama	MH107359	MG973683	MG973489	MG973576
Epiperipatus sp.	MZUSP0112	Panama	MH107360	MG973684	MG973490	MG973577
Epiperipatus sp.	MZUSP0073	Colombia	MN905702	MN639428	MN544180	MN705471
Epiperipatus titanicus	MNRJ0053	Brazil	MN905652	MN639378	MN544128	MN705446
Epiperipatus vagans	MZUSP0101	Panama	MH107349	MG973663	MG973482	MG973544
Epiperipatus vagans	MZUSP0113	Panama	MH107348	MG973664	MG973483	MG973545
Epiperipatus vagans	MZUSP0114	Panama	MH107350	MG973665	MG973484	MG973547
Epiperipatus vagans	MZUSP0115	Panama	MH107347	MG973666	MG973485	MG973546
Macroperipatus valerioi	MCZ130841	Costa Rica	MH107341	MG973681	MG973496	MG973558
Macroperipatus valerioi	MCZ130842	Costa Rica	MH107342	MG973682	MG973497	MG973569
Mesoperipatus tholloni	MCZ131381	Gabon	KC754645	KC754478	KC754528	KC754576
Mongeperipatus solorzanoi	MZUSP0013	Costa Rica	KM095130	MN639393	MN544147	MN705454
Oroperipatus cf. *corradi*	MNRJ0073	Ecuador	MN905630	MN639358	MN544106	MN705440
Oroperipatus corradi	MNRJ0071	Ecuador	MN905629	MN639357	MN544105	MN705439
Oroperipatus eisenii	MCZ71297	Mexico	MH107369	MG973712	MG973531	MG973599
Oroperipatus eisenii	ZMH-ARA-A00320101	Mexico	PX876296	PX746775	PX746765	PX880754
Oroperipatus lankesteri	BMNH(E)1004870	Ecuador	–	–	PX972671	–
Oroperipatus megapiculatus **sp. nov.**	QCAZI–282081	Ecuador	PX876303	PX746784	PX746774	PX880762
Oroperipatus palenquensis **sp. nov.**	QCAZI–282083	Ecuador	PX876300	PX746779	PX746769	PX880758
Oroperipatus peruvianus	ZMH-ARA-A0032096	Peru	–	PX746780	PX746770	PX880759
Oroperipatus sachaensis **sp. nov.**	QCAZI–282084	Ecuador	PX876298	PX746777	PX746767	PX880756
Oroperipatus similaris **sp. nov.**	QCAZI–282085	Ecuador	PX876302	PX746783	PX746773	PX880761
Oroperipatus sp.	MCZ131423	Ecuador	KC754637	KC754472	KC754520	MG973596
Oroperipatus sp.	MCZ131425	Colombia	KC754641	KC754475	KC754524	MG973598
Oroperipatus sp.	MNRJ0072	Ecuador	MN905636	MN639362	MN544111	MN705442
Oroperipatus sp.	MNRJ0069	Ecuador	MN933398	MG973711	MN933786	MG973604
Oroperipatus sp.	MCZ131424	Ecuador	KC754643	KC754477	KC754526	MG973597
Oroperipatus sp.	MCZ43390	Galapagos	MH107370	MG973713	KM102162	MG973600
Oroperipatus sp.	MCZ43392	Galapagos	MH107371	MG973714	MG973533	MG973602
Oroperipatus sp.	MCZ43394	Galapagos	MH107372	MG973716	MG973532	MG973603
Oroperipatus sp.	MCZ43401	Galapagos	MH107373	MG973715	KM102163	MG973601
Oroperipatus specialis **sp. nov.**	QCAZI–282086	Ecuador	PX876297	PX746776	PX746766	PX880755
Oroperipatus vallartensis **sp. nov.**	CNAC/Ony00086	Mexico	PX876299	PX746778	PX746768	PX880757
Oroperipatus variabilis **sp. nov.**	QCAZI–282087	Ecuador	PX876301	PX746781	PX746772	PX880760
Oroperipatus variabilis **sp. nov.**	ZMH-ARA-A00320103	Ecuador	PX956827	PX972669	PX972670	PX957499
Peripatidae	MZUSP0106	Panama	MH107364	MG973686	MG973493	MG973573
Peripatidae	MZUSP0108	Panama	MH107361	MG973687	MG973495	MG973575
Peripatidae	MCZ32028	Venezuela	MH107366	MG973676	MG973524	MG973581
Peripatus basiliensis	MCZ131422	Dominican Republic	KC754646	MG973700	MG973471	MG973563
Peripatus bouvieri	ICN–ONY–27	Colombia	MN905677	MN639400	MN544153	MN705458
Peripatus bouvieri	ICN–ONY–28	Colombia	MN905678	MN639401	MN544154	MN705459
Peripatus bouvieri	ICN–ONY–13	Colombia	MN905679	MN639402	MN544155	MN705460
Peripatus sp.	MCZ46445	Guyana	MH107337	MG973703	MG973470	MG973583
Principapillatus hitoyensis	MCZ131339	Costa Rica	KC754642	KC754476	KC754525	MG973556
Principapillatus hitoyensis	MCZ131340	Costa Rica	MH107340	MG973680	MG973488	MG973555

Table S3. Gene partition length on different datasets used for phylogenetic analyses, Related to Results.

**Genes**	**Without *O. lankesteri***			**With *O. lankesteri***	
	**Dataset 1**	**Dataset 2**	**Dataset 3**	**Dataset 4**	**Dataset 5**
12S rRNA	304			304	
16S rRNA	336			336	
18S rRNA	1588			1588	
COI (all nucleotides)	635	–	–	635	–
COI (1st and 2nd codon positions)	–	424	–	–	424
COI (translated to amino acids)	–	–	242	–	–
Total	2863	2652	2470	2863	2652
